# The gut microbiome organ

**DOI:** 10.1002/imt2.70144

**Published:** 2026-08-21

**Authors:** Yang Bi, Weibin Song, Maria Glymenaki, Jie Hu, Xiru Li, Heng Huang, Yousong Peng, Shuaishuai Ni, Ornuma Haonon, Zhu Liu, Krai Daowtak, Xiaotao Shen, Huadong Peng, Jutarop Phetcharaburanin, Huiru Tang, Yulan Wang, Jia V. Li, Mizu Jiang, Nick Powell, Victor W. Zhong, Elaine Holmes, Yunlei Xianyu, Zhigang Liu

**Affiliations:** ^1^ Children's Hospital, Zhejiang University School of Medicine, National Clinical Research Center for Children and Adolescents' Health and Diseases Hangzhou China; ^2^ Luoyang Central Hospital Affiliated to Zhengzhou University Luoyang China; ^3^ Department of Metabolism Digestion and Reproduction, Faculty of Medicine, Imperial College London London UK; ^4^ European Food Safety Authority, Nutrition and Food Innovation Unit, Novel Foods Team Parma Italy; ^5^ Bioinformatics Center, College of Biology, Hunan Provincial Key Laboratory of Medical Virology, Hunan Research Center of the Basic Discipline for Cell Signaling Hunan University Changsha China; ^6^ Cancer Institute, Longhua Hospital Shanghai University of Traditional Chinese Medicine Shanghai China; ^7^ Cellular and Molecular Immunology Research Unit, Faculty of Allied Health Sciences Naresuan University Phitsanulok Thailand; ^8^ Lee Kong Chian School of Medicine Nanyang Technological University Singapore Singapore; ^9^ School of Chemistry, Chemical Engineering and Biotechnology Nanyang Technological University Singapore Singapore; ^10^ Singapore Phenome Center Nanyang Technological University Singapore Singapore; ^11^ UQ Biosustainability Hub, Australian Institute for Bioengineering and Nanotechnology The University of Queensland Brisbane Australia; ^12^ ARC Center of Excellence in Synthetic Biology The University of Queensland Brisbane Australia; ^13^ Food and Beverage Accelerator (FaBA) The University of Queensland Brisbane Australia; ^14^ The National Phenome Institute, Office of the President, Khon Kaen University Khon Kaen Thailand; ^15^ Department of Systems Biosciences and Computational Medicine, Faculty of Medicine Khon Kaen University Khon Kaen Thailand; ^16^ State Key Laboratory of Genetic Engineering, School of Life Sciences, Metabonomics and Systems Biology Laboratory at Shanghai International Center for Molecular Phenomics, Zhongshan Hospital, Zhangjiang Fudan International Innovation Center Fudan University Shanghai China; ^17^ Human Phenome Institute, Fudan University Shanghai China; ^18^ Department of Epidemiology and Biostatistics, School of Public Health Shanghai Jiao Tong University School of Medicine Shanghai China; ^19^ School of Medicine, The Hong Kong University of Science and Technology Clear Water Bay Kowloon Hong Kong China; ^20^ College of Biosystems Engineering and Food Science, Zhejiang Key Laboratory of Agro‐food Resources and High-value Utilization Zhejiang University Hangzhou China; ^21^ Department of Clinical Laboratory Sir Run Run Shaw Hospital, Zhejiang University School of Medicine Hangzhou China

**Keywords:** gut microbiome organ, gut microbiota, host–microbiome interactions, microbial metabolites, metabolic–immune–neuroendocrine integration, multi‐omics, precision microbiome medicine

## Abstract

The human gut microbiome is increasingly viewed as an active regulator of host physiology, extending beyond earlier taxonomy‐centered descriptions of a complex microbial community. Accumulating evidence supports an organ‐like conceptual framework in which the gut microbiome exhibits spatially structured organization, extensive metabolic capacity, and continuous bidirectional communication with host systems. Through the production of bioactive metabolites with endocrine‐like, immunomodulatory, and neuromodulatory properties, the microbiome contributes to metabolic, immune, and neuroendocrine regulation, thereby influencing systemic homeostasis and disease susceptibility. Recent advances in multi‐omics, spatial biology, and computational modeling are moving the field from taxonomic association toward functional interpretation, mechanistic insight, and causal inference. These approaches are beginning to reveal microbiome‐derived functional modules and host–microbe signaling networks that are shaped by host genetics, diet, medications, feeding patterns, circadian rhythms, and environmental exposures. In this review, we synthesize current mechanistic and translational evidence to conceptualize the gut microbiome as an organ‐like functional system, delineate its structural and functional organization, and propose a framework for mapping, modeling, and therapeutically targeting microbiome‐derived circuits to support precision medicine in metabolic, inflammatory, and selected gut–brain axis‐related disorders.

## Highlights

- The gut microbiome can be conceptualized as an organ‐like functional system with spatial organization, metabolic capacity, and systemic regulatory potential.
- Microbial metabolites can function as endocrine‐like mediators linking metabolic, immune, and neuroendocrine pathways.
- Multi‐omics and spatial approaches are beginning to reveal functional microbiome modules and host–microbe signaling networks relevant to health and disease.
- Targeting microbiome function, rather than individual taxa alone, provides a rational framework for mechanistic investigation, causal inference, and precision therapeutics.

## Graphical abstract

The gut microbiome can be conceptualized as a distributed organ‐like functional system with spatially structured organization, broad biochemical capacity, and continuous bidirectional communication with the host. By transforming dietary, host‐derived, and environmental substrates into bioactive metabolites with endocrine‐like, immunomodulatory, and neuromodulatory activities, the microbiome contributes to metabolic, immune, and neuroendocrine pathways that shape systemic homeostasis and disease susceptibility. Emerging spatial, multi‐omics, and computational approaches are enabling systems‐level mapping of microbiome architecture, functional modules, and host–microbe signaling networks, moving the field beyond descriptive taxonomy toward mechanistic insight and causal inference. This organ‐level framework provides a conceptual and translational basis for developing precision microbiome medicine through the identification of targetable pathways, functional circuits, and personalized therapeutic strategies.

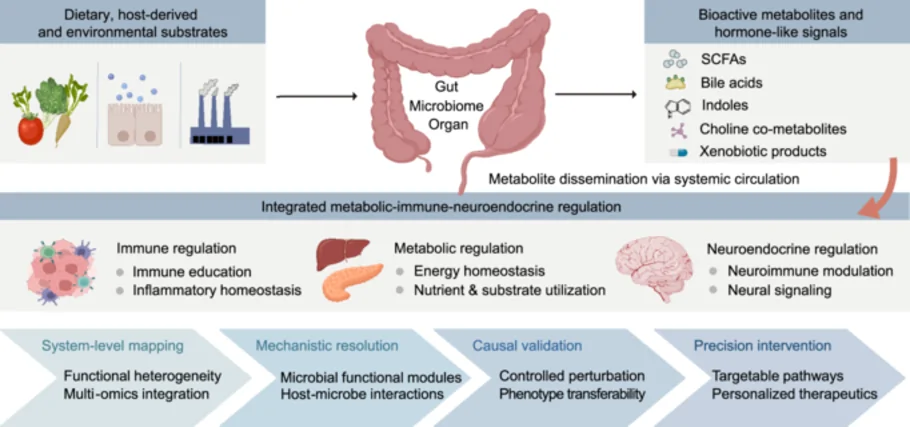

## INTRODUCTION

The human gut microbiome has moved beyond its earlier taxonomy‐centered descriptions of a compositionally defined microbial community and is now increasingly understood as a functionally integrated component of host biology. Large‐scale metagenomic and multi‐omic studies have shown that the microbiome encodes extensive biochemical capacity, displays partly conserved functional potential despite taxonomic variability, and participates in the regulation of metabolic, immune, and epithelial homeostasis [1, 2, 3, 4, 5]. These observations have revived and refined earlier concepts of the gut flora as a “forgotten organ” and the microbiome as a microbial, virtual, or endocrine‐like organ [6, 7, 8, 9]. In this context, the gut microbiome should not be regarded as an organ in the classical anatomical sense, but as a distributed, spatially organized, and metabolically active biological system whose physiological functions emerge from coordinated host–microbe and microbe–microbe interactions.

Building on this literature, we use the term “gut microbiome organ” as a heuristic and operational framework rather than as a strict anatomical definition. From this systems‐level perspective, the gut microbiome can be viewed as a spatially organized, functionally interconnected, and communicative entity that performs integrated physiological tasks and modulates local and distal host processes through molecular signaling networks. It occupies structured ecological niches along the longitudinal and transverse axes of the gastrointestinal tract, ranging from segment‐specific luminal communities to mucus‐associated and epithelial‐adjacent microbial populations, particularly in specific physiological or pathological contexts [10, 11]. It also encodes a large metabolic repertoire that complements host biochemical capacity, including the fermentation of otherwise indigestible carbohydrates, transformation of bile acids, metabolism of amino acids, and processing of dietary, host‐derived, and xenobiotic substrates [2, 4, 5]. Through these activities, the microbiome generates bioactive metabolites, some of which exert endocrine‐like or neuromodulatory effects, including short‐chain fatty acids, indole derivatives, secondary bile acids, microbial trimethylamine and host‐derived trimethylamine *N*‐oxide, and other neuroactive or neuromodulatory metabolites, which signal through host receptors and pathways to influence epithelial integrity, immune development, energy balance, and neuroendocrine function [4, 5, 9].

This organ‐level framework also helps clarify why taxonomic descriptions alone are insufficient to explain microbiome function. The physiological effects of the microbiome arise not from individual taxa in isolation, but from coordinated microbial networks, spatially constrained ecological niches, and functional modules that interact dynamically with host pathways. A given microbial community may therefore have different biological consequences depending on its spatial location, substrate availability, host immune state, developmental stage, and environmental exposures. Conversely, different taxonomic configurations may converge on similar functional outputs if they preserve shared metabolic or signaling capacities. This functional redundancy and context‐dependence support a shift from compositional profiling toward mechanistic analysis of microbial activities, metabolites, and host‐response circuits.

Recent advances in spatial biology, longitudinal multi‐omics, and computational modeling have further strengthened this perspective. The Integrative Human Microbiome Project and disease‐focused multi‐omic studies have demonstrated that microbial genes, transcripts, proteins, metabolites, and host responses can be integrated to characterize dynamic host–microbiome activity across health and disease states [12, 13]. More recent spatial host–microbiome technologies have enabled direct mapping of bacterial localization, host transcriptional responses, and, increasingly, microbial functional or transcriptional states within intact tissue microenvironments, revealing that microbiome function is inseparable from ecological architecture [14, 15, 16]. Together, these advances are moving the field from static association studies toward systems‐level models of microbiome‐derived functional circuits.

In this review, we examine the gut microbiome within this organ‐level framework. We first define the structural and ecological architecture of the gut microbiome organ, emphasizing spatial organization, niche specialization, and life‐course dynamics. We then discuss its functional modules and metabolic capacities, focusing on how microbial transformations generate bioactive signals that regulate host physiology. We next examine the microbiome as a metabolic, immune and neuroendocrine interface, and consider how disruption of these circuits contributes to disease pathogenesis. Finally, we address translational challenges and future opportunities for mapping, modeling, and therapeutically targeting microbiome organ function in precision medicine.

## THE GUT MICROBIOME AS A STRUCTURED AND DYNAMIC ORGAN

### Overview

**FIGURE 1:**
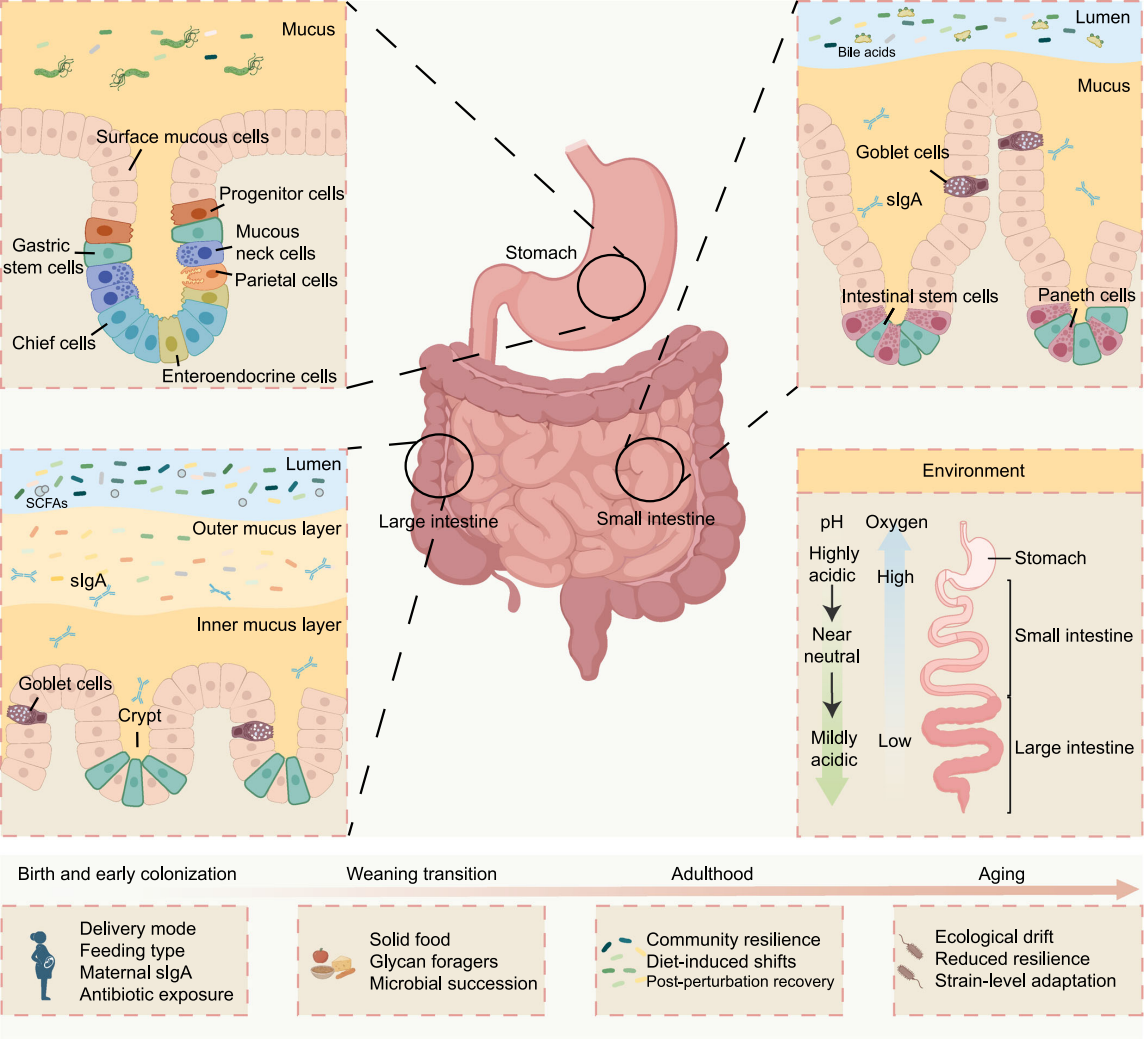
Spatial organization and host regulation of the gut microbiome organ. The gut microbiome functions as an organ‐like system structured along both longitudinal and transverse axes of the gastrointestinal tract. Regional physicochemical gradients shape site‐specific microbial communities and metabolite profiles, whereas mucus, epithelial niches, and IgA‐mediated immune filtering contribute to spatial segregation and host control. Across the lifespan, the microbiome is assembled through early‐life colonization and weaning‐associated succession, remains relatively resilient in adulthood, and undergoes ecological drift and strain‐level adaptation during aging. sIgA, secretory immunoglobulin A; SCFAs, short‐chain fatty acids.

The gut microbiome exhibits a multiscale spatial architecture organized across macroscopic and microscopic scales that is central to many of its roles in host physiology, metabolism, and immunity [11]. Its structure is characterized by marked spatial heterogeneity, with distinct microbial communities occupying specialized niches along the longitudinal axis of the gastrointestinal tract, particularly from the small intestine through the colon, as well as across the transverse axis from the lumen to the mucosal surface and crypt‐adjacent or epithelial‐associated microenvironments (Figure 1) [11]. This biogeography is actively shaped by physicochemical gradients of oxygen, pH, nutrients, bile acids, antimicrobial peptides, mucus properties, and flow rate, which act as ecological filters selecting region‐specific microbial guilds [17].

At the center of this organization is the mucosal interface, a dynamic frontier where host and microbe interact continuously. The mucus layer functions simultaneously as a protective barrier and a nutrient‐rich habitat, with host‐derived glycans providing a renewable substrate that sustains specialized microbial populations [18]. Microbes contribute to this architecture through adhesion, biofilm‐like organization in selected niches, and cell–cell communication, processes that can provide structural scaffolds and enable cooperative metabolic networks [19]. Microbiome organization is further refined by host factors, including genetic predispositions and immunological pressures, with host genes involved in innate and adaptive immunity and metabolism associated with variation in microbial diversity, and epithelial metabolism helping to shape regional microbial environments [20, 21].

The assembly and maturation of this organ‐like microbial system begin in early life and continue across the life course, followed by succession during weaning and subsequent remodeling through adulthood and aging, combining stability with age‐associated drift [22]. Understanding this structured and dynamic nature is essential for deciphering the microbiome's contribution to health and its dysregulation in disease. Disruption of spatial organization represents an important mechanistic dimension of microbiome dysbiosis, whereby breakdown of ecological compartmentalization may promote functional and immunological imbalance.

The roots of the concept of the microbiome as an organ lie in earlier literature considering the human as a supraorganism or host–microbial metabolic system with “extensive coordination of metabolic and physiological responses” [23] or the microbiome as part of the human genetic and metabolic landscape with implications for normal physiology and disease susceptibility [24]. Several reviews and perspectives have referred to the gut microbiome as the neglected or forgotten (endocrine) organ [6, 9], while other reviews have extended this organ‐like concept to the pathophysiology of specific pathologies such as uremia [25], Alzheimer's disease [26], or COVID‐19 [27]. Here, we develop the concept further, exploring the structural architecture and biochemical organization of the gut microbiome as an organ‐like functional system.

### Structural architecture of the gut microbiome organ

#### Macro‐ and micro‐scale biogeography of the gut microbiota

The gut microbiome exhibits a highly structured spatial organization forming distinct communities at macroscopic and microscopic scales [11]. A defining feature is regional heterogeneity along the longitudinal axis of the gastrointestinal tract, where each segment harbors microbial populations adapted to local physiological conditions [28]. The small intestine, characterized by rapid nutrient flux and immune surveillance, differs markedly from the fermentative environment of the large intestine [29]. Consequently, fecal samples predominantly capture luminal communities from the distal gut and may inadequately represent proximal or mucosa‐associated regions, emphasizing the importance of site‐specific sampling [28]. This regionalization is embedded in the intestinal tissue landscape yet remains responsive to microbiota‐ and inflammation‐related perturbations [30]. Host transcription factors such as GATA4 contribute to this spatial patterning by regulating bacterial colonization and tissue immunity in the proximal small intestine, in part through retinol metabolism and luminal IgA [31]. These host‐driven factors collectively shape the local biochemical and immunological niche, thereby helping to constrain microbial colonization and function and contributing to the establishment of region‐specific microbial communities.

Spatial organization of the microbiome also extends across the transverse axis, generating distinct microhabitats from lumen to epithelium [11]. Luminal and mucosa‐associated communities are compositionally distinct [32]. Mucosa‐associated communities, including the virome, can differ from luminal or stool‐derived populations [32, 33], while intestinal crypts can serve as protected reservoirs for selected commensals, such as *Bacteroides fragilis*, in specific experimental contexts, whose commensal colonization factors enable persistent niche occupation and resilience following perturbation [34]. Advances in spatial host–microbiome profiling, including spatial host–microbiome sequencing approaches, have enabled high‐resolution mapping of mucosal niches and revealed spatially resolved bacterial localization patterns together with host transcriptional responses associated with these environments [35].

This biogeography is governed by ecological filters imposed by host physiology [17]. Gradients in oxygen tension, nutrient availability, and redox potential create selective pressures shaping community composition [36]. Colonic epithelial metabolism is central to this process: oxygen consumption by colonocytes maintains epithelial hypoxia that favors obligate anaerobes involved in fiber fermentation, whereas disruption promotes expansion of facultative anaerobes [21]. Host metabolites such as bile acids further contribute to regionalization through antimicrobial effects and immune modulation; microbial bile acid transformation can prime localized interferon responses that influence pathogen susceptibility along the gut [37]. Collectively, these host‐derived gradients establish the ecological framework underlying microbial spatial organization [36].

#### The mucosal interface: A dynamic frontier for host–microbe symbiosis

The intestinal mucosal interface orchestrates host–microbe symbiosis through the mucus layer, a viscoelastic gel composed primarily of glycosylated mucin proteins that serves a dual protective and ecological role [18]. In the colon, mucus forms a dense, largely bacteria‐excluding inner layer adjacent to the epithelium and a looser outer layer that provides habitat and nutrients for mucus‐associated microbiota [18, 38]. Antimicrobial peptides retained within mucus help create a localized antibacterial zone that preserves spatial separation while permitting commensal colonization in the lumen [39]. Disruption of mucus integrity can increase microbial access to epithelial surfaces and is associated with inflammatory contexts such as ulcerative colitis (UC) and enhanced infection susceptibility [18, 40].

Mucin glycoproteins are decorated with diverse O‐glycans that constitute a continuously renewable nutrient supply shaping microbial community composition [41]. Host regulation of glycosylation provides a mechanism to support commensals during stress; for example, infection‐induced epithelial fucosylation via IL‐23/IL‐22 signaling supplies fucose to resident microbes, modulating metabolism and reducing pathogen virulence gene expression [42]. IL‐22‐dependent epithelial fucosylation and related glycosylation programs also contribute to colonization resistance against pathogens such as *Clostridioides difficile* by fostering commensals that compete for shared metabolic niches [43].

Microbial adaptation to this glycan landscape involves specialized enzymatic machinery for mucin degradation. Symbionts, including *Bacteroides thetaiotaomicron* and *Akkermansia muciniphila*, devote substantial genomic capacity to sensing and catabolizing host glycans through dedicated glycan‐utilization systems and carbohydrate‐active enzymes such as glycoside hydrolases and sulfatases [41, 44, 45]. A single sulfatase in *Bacteroides thetaiotaomicron* is critical for the utilization of sulfated O‐glycans in the distal colon [41]. *Akkermansia muciniphila* employs sialidases and fucosidases that liberate sugars subsequently cross‐fed to other microbes, including butyrate‐producing *Clostridia* [46]. Some *Ruminococcus gnavus* strains possess unique intramolecular trans‐sialidases that support adaptation to the mucus niche [47]. Glycan foraging can therefore represent a key fitness trait supporting intestinal persistence and, in some contexts, transmission between hosts or across generations [45].

Importantly, this spatial organization is highly susceptible to pathological remodeling under disease conditions. In inflammatory bowel disease (IBD), disruption of the inner mucus layer permits direct bacterial encroachment onto the epithelium, leading to loss of spatial segregation and sustained immune activation [18, 40]. This process is accompanied by impaired mucin production, altered glycosylation patterns, and altered abundance or activity of mucus‐associated and mucin‐degrading taxa, including potential pathobionts, collectively destabilizing the mucosal niche. In parallel, inflammation‐driven changes in epithelial metabolism disrupt oxygen gradients, increasing epithelial oxygenation and favoring the expansion of facultative anaerobes such as Enterobacteriaceae [48]. These shifts represent disruption of the anaerobic niche structure that normally constrains microbial composition. Similar, though less acute, spatial remodeling has been described in diet‐induced metabolic contexts, in which high‐fat or low‐fiber diets may reduce mucus thickness, alter microbial localization, and promote low‐grade barrier dysfunction [18, 40]. Together, these findings indicate that disease‐associated dysbiosis is not solely compositional but can also involve a breakdown of the spatial architecture that underpins host–microbiome homeostasis.

#### Microbial self‐organization and interspecies interactions

Gut microbial communities are actively structured through self‐organization processes, including adhesion, aggregation, and biofilm‐like organization in selected niches [49, 50, 51]. Biofilms are spatially organized polymicrobial assemblies embedded in extracellular matrices that adhere to host surfaces or dietary particles, creating microenvironments with distinct physiological properties [50]. This lifestyle enhances resistance to environmental stressors and facilitates interspecies interactions [51]. Pathogenic biofilms contribute to disease processes, as observed in enterococcal infections and colorectal cancer‐associated biofilm communities [52]. The opportunistic pathogen *Clostridioides difficile* has been shown experimentally to form enhanced biofilms within intestinal mucus through interaction with *Fusobacterium nucleatum* [53]. Commensals also rely on adhesion strategies; *Bacteroides fragilis* uses conserved colonization factor genes to occupy crypt niches that provide protection from perturbations [34].

Metabolic cooperation and cross‐feeding networks further stabilize these structured communities. Breakdown of complex carbohydrates often requires coordinated enzymatic activity among species, producing syntrophic relationships that enhance ecosystem efficiency [54]. For example, *Bacteroides ovatus* degrades inulin at a metabolic cost, generating substrates that benefit *Bacteroides vulgatus*, which reciprocally supports *Bacteroides ovatus* fitness [19]. Mucin degradation by *Akkermansia muciniphila* releases sialic acid and fucose that nourish mucus‐associated butyrate‐producing *Clostridia* and promote butyrate production [46]. Cross‐feeding interactions extend to butyrate‐producing *Clostridiales*, including *Roseburia* and *Eubacterium* species, some of which can metabolize human milk oligosaccharides and mucin‐derived glycans through overlapping glycan‐foraging pathways [55]. *Bifidobacteria* similarly participate in extensive glycan sharing, underscoring metabolic cooperation as a cornerstone of microbial ecology [56].

Coordination of collective behaviors is mediated by quorum sensing, a chemical communication system enabling bacteria to regulate gene expression in response to population density [57]. Quorum sensing can regulate biofilm formation, virulence‐associated traits, and host‐associated colonization behaviors [58, 59]. In gut environments, signaling pathways such as those described in *Serratia* can promote biofilm‐like aggregation and adaptation to host niches [60]. Production of quorum sensing molecules such as 3,5‐dimethylpyrazin‐2‐ol (DPO) by diverse commensals suggests that chemical signaling is a potentially widespread feature shaping community dynamics and host–microbe interactions [61]. Understanding this communication network remains essential for elucidating mechanisms of microbial self‐organization and stability within the gut ecosystem [57].

#### Host‐mediated sculpting of microbiome architecture

The architecture of the gut microbiome, while shaped by microbial self‐organization, is also strongly shaped by host determinants. This high degree of ecological and functional plasticity allows microbial communities to dynamically respond to environmental and host‐derived perturbations. Microbial community remodeling can occur rapidly in response to changes in nutrient availability, inflammation, or physicochemical gradients, leading to shifts in both composition and metabolic output within specific niches. In parallel, horizontal gene transfer provides a mechanism for rapid functional adaptation that may occur without requiring major taxonomic turnover, enabling resident microbes to acquire accessory genetic elements with potential functional consequences [62]. These processes can partly decouple functional capacity from community structure, such that similar taxonomic profiles may exhibit distinct metabolic behaviors across microenvironments.

Host genetics provides one layer of control over microbial composition and structure, as demonstrated by candidate gene studies and quantitative genetic analyses linking host variation to microbiota profiles [20]. For example, deletion of the Notch pathway regulator *Pofut1* alters intestinal lineage differentiation, resulting in mucus hypersecretion, modified mucus‐associated communities, and chronic inflammation [63]. Multi‐cohort analyses in genetically diverse models have further identified host loci associated with microbial taxa abundance, including a locus containing *St6galnac1* linked to *Paraprevotella* levels [64]. Similar host–microbiota genetic interactions have been described in livestock, indicating that a subset of microbes is associated with host genetic variation to influence complex traits such as feed efficiency [65]. These findings highlight that host genetics can contribute to selective pressures that shape microbial assembly alongside environmental factors.

The innate immune system exerts continuous ecological pressure on microbial communities, shaping habitat structure and spatial distribution [17]. Epithelial metabolism is central to this process; colonocyte oxygen consumption maintains epithelial hypoxia that favors obligate anaerobes while restricting the expansion of facultative anaerobes, including potential pathobionts [21]. Spatially organized innate immune signaling is at least partly developmentally programmed and contributes to regional immune environments even in the absence of microbial exposure [66]. For instance, GATA4 regulates regional immunity through control of retinol metabolism and IgA production in the small intestine [31]. Antimicrobial peptides such as Regenerating islet‐derived protein 3 gamma (REG3γ) accumulate within mucus, maintaining spatial segregation between microbes and the epithelium without sterilizing the lumen [39, 67]. Innate lymphoid cells, particularly ILC3s, reinforce this structural control by producing IL‐22. IL‐22 signaling further modifies epithelial glycosylation and reshapes nutrient availability, thereby helping to limit *Clostridioides difficile* expansion [43]. Together, innate mechanisms function as habitat filters that maintain microbiome biogeography.

Adaptive immunity provides an additional, selective layer of architectural control through secretory IgA [68]. As the predominant mucosal antibody, IgA coats a variable but substantial fraction of the microbiota and contributes to immune homeostasis [69]. IgA responses arise via T cell‐dependent and T cell‐independent pathways, generating repertoires ranging from polyreactive to highly specific antibodies targeting bacteria and fungi, including fungal morphotypes associated with pathogenic or invasive states [70, 71]. IgA‐producing B cells are selected within gut‐associated lymphoid tissues under regulation by T follicular mechanisms, processes influenced by microbial antigens and metabolites [72, 73]. Techniques such as IgA‐SEQ have identified IgA‐coated taxa in both health and IBD, with disease‐associated enrichment of selected IgA‐coated taxa in IBD cohorts, illustrating the role of IgA targeting in host‐microbe equilibrium [74].

IgA coating exerts multifaceted effects on microbial fitness and localization [75]. Binding can restrict bacterial motility and, in broader mucosal contexts, contribute to mucus‐associated containment and reduce epithelial access, thereby reinforcing spatial segregation [75, 76]. Metagenomic analyses indicate that IgA association can correlate with reduced bacterial replication rates, suggesting population‐level regulation [77]. However, IgA can also modulate microbial physiology; monoclonal IgA studies show epitope‐specific effects on metabolism and bacteriophage susceptibility [76]. In certain contexts, IgA coating can enhance symbiosis by inducing or selecting for gene expression patterns that facilitate niche colonization and interspecies cooperation within mucus environments [78]. Thus, IgA acts not as a purely exclusionary mechanism but as a nuanced regulator of microbial behavior, community structure, and spatial organization [75, 79].

#### Temporal dynamics and the assembly of the microbiome organ

The gut microbiome assembles through a dynamic life‐course trajectory that is most clearly initiated around birth and evolves across developmental stages. Early‐life colonization establishes founder communities shaped by maternal and environmental factors, including delivery mode, feeding practices, and antibiotic exposure [80]. Early microbial antigens can influence neonatal immune development, including shaping B‐1 cell repertoires, while perturbations during this window can have lasting consequences [81]. Prenatal antibiotic exposure, for example, has been shown in mouse models to reduce breast milk IgA, induce offspring dysbiosis, and increase neonatal susceptibility to bacterial infection [82]. Developmental immune cell populations, including early‐life macrophage populations, are also influenced by the nascent microbiota, contributing to early immune architecture [83].

Weaning represents another critical developmental window marked by dietary transition and ecological restructuring of the microbiome [84]. The transition to solid food and associated changes in available carbohydrates create new metabolic niches, including niches for microbes capable of degrading dietary and host‐derived glycans. Early infant communities are often enriched for milk‐oligosaccharide‐utilizing taxa and may have more limited mucin‐degrading capacity than later communities, whereas taxa such as *Akkermansia muciniphila* and glycan‐utilizing *Clostridiales* may gain a competitive advantage during this transition [55, 85]. This succession contributes to immune maturation and the establishment of adult‐like microbial networks.

In adulthood, the microbiome typically reaches a relatively stable yet resilient state, although recovery from disturbances varies with perturbation type, host context, and baseline community structure [36]. Stability is reinforced by host–microbe interactions and ecological priority effects that limit invasion by new taxa [36, 86]. Nevertheless, long‐term dietary patterns can induce partly reversible compositional and functional shifts, exemplified by Western diet‐associated remodeling [87]. Aging is associated with compositional drift and may be accompanied by strain‐level adaptation or pathoadaptive evolution in resident microbes such as *Escherichia coli*, reflecting adaptation to altered host environments [88, 89]. Differences between frail and healthy elderly individuals further support the continuing relationship between microbiome structure and host health across the lifespan [90].

### Section summary

The gut microbiome operates as a structured and dynamic organ‐like system whose architecture underlies its symbiotic relationship with the host. Microbial communities are partitioned along longitudinal and transverse axes into specialized niches, including lumen, mucus, and crypt habitats [11]. This spatial organization is shaped by host‐derived ecological filters, including physicochemical gradients and epithelial metabolism, which favor specific microbial groups [11, 21]. The mucosal interface functions as both barrier and nutritional niche, with host glycans shaping microbial composition and activity [41]. Microbial self‐organization through adhesion, biofilm formation, and metabolic cooperation, contributes to structural and functional community organization [11, 19]. Host determinants, ranging from genetic variation to innate and adaptive immune mechanisms such as IgA, continuously shape and refine this architecture [20]. Microbiome assembly follows a life‐course trajectory beginning in infancy, being reshaped during weaning, and gradually remodeled through adulthood and aging [85].

A critical unresolved question is how disruption of spatial organization causally contributes to disease pathogenesis. Emerging evidence indicates that loss of compartmentalization, characterized by microbial encroachment, breakdown of mucus barriers, and altered physicochemical gradients, is an important feature that may link dysbiosis to host inflammation and metabolic dysfunction [18, 40]. In this framework, spatial remodeling may represent a mechanistic intermediate between environmental perturbations such as diet, antibiotics, and inflammation and downstream immune and metabolic phenotypes. Rather than being solely compositional, disease‐associated dysbiosis may therefore reflect, at least in part, a failure of the ecological and structural constraints that normally maintain host–microbiome homeostasis. Defining these causal relationships will require integrating spatial multi‐omics with controlled perturbation models to distinguish primary architectural defects from secondary consequences of disease.

Translating architectural insights into therapeutic strategies also presents significant challenges. Rational manipulation of spatial organization through dietary interventions, targeted probiotics, or refined microbiota transplantation remains limited by an incomplete understanding of niche‐specific colonization and stability. Strategies that reinforce mucus integrity, restore anaerobic microenvironments, disrupt pathogenic biofilms, or promote spatially resolved engraftment of beneficial microbes may become important translational priorities.

Future research will require high‐resolution mapping of microbiome architecture using integrated spatial multi‐omics approaches that combine microbial profiling with single‐cell immune analysis across intestinal regions [91]. Development of in vitro systems that recapitulate intestinal heterogeneity, including mucus‐associated and luminal microbiome compartments, will facilitate mechanistic studies [92]. Ultimately, a systems‐level understanding of microbiome assembly, stability, and spatial organization could support precision interventions designed not only to alter microbial composition but also to restore the structural foundations of host‐microbiome symbiosis.

## FUNCTIONAL MODULES OF THE MICROBIOME ORGAN

### Overview

**FIGURE 2:**
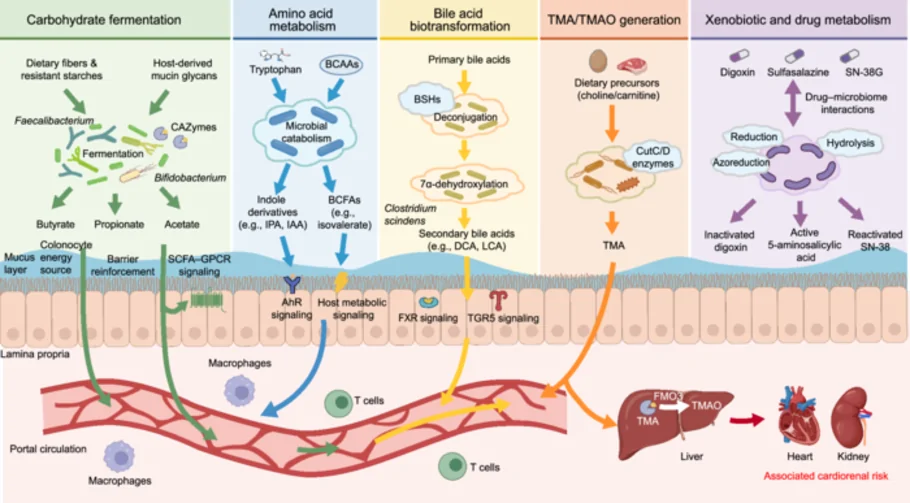
Functional metabolic modules of the gut microbiome organ. The gut microbiome operates as an integrated organ‐like metabolic system composed of distinct but interconnected functional modules. These include carbohydrate fermentation, amino acid metabolism, bile acid biotransformation, TMA–TMAO generation, and xenobiotic and drug metabolism. Each module processes dietary, host‐derived, or environmental substrates through specialized microbial enzymes or microbial consortia, generating bioactive metabolites that interact with epithelial barriers, immune cells, host receptors, hepatic metabolism, and systemic circulation. Together, these pathways regulate epithelial energy supply, barrier integrity, immune and metabolic signaling, cardiovascular and kidney disease‐related risk pathways, and drug activation, inactivation, or reactivation in selected contexts. AhR, aryl hydrocarbon receptor; BCAAs, branched‐chain amino acids; BCFAs, branched‐chain fatty acids; BSHs, bile salt hydrolases; CAZymes, carbohydrate‐active enzymes; DCA, deoxycholic acid; FMO3, flavin‐containing monooxygenase 3; FXR, farnesoid X receptor; GPCRs, G protein‐coupled receptors; IAA, indole‐3‐acetic acid; IAld, indole‐3‐carboxaldehyde; IPA, indole‐3‐propionic acid; LCA, lithocholic acid; PXR, pregnane X receptor; SCFAs, short‐chain fatty acids; SN‐38G, SN‐38 glucuronide; TGR5, Takeda G protein‐coupled receptor 5; TMA, trimethylamine; TMAO, trimethylamine N‐oxide.

The gut microbiome functions as a dynamic biochemical processing hub, engaging in a complex metabolic crosstalk with the host that influences nutrient harvesting, xenobiotic metabolism, and immune regulation [93]. The immense genetic and enzymatic diversity of the gut microbiota enables it to perform chemical transformations that complement or are distinct from host metabolism, effectively extending the host's metabolic capabilities [94, 95]. To deconstruct this complexity, it is useful to conceptualize the microbiome's metabolic output through the framework of functional modules (Figure 2). Each module can be defined by its characteristic substrate inputs, the specific microbial processes that act upon them, the resulting metabolic outputs, and the downstream physiological effects on the host [96]. This modular approach provides a structured way to understand how specific microbial activities are coupled to host metabolic phenotypes and disease‐related states [97].

Importantly, this functional framework extends beyond bacterial communities. Although bacteria constitute the dominant and most extensively studied component of the gut microbiome, non‐bacterial microorganisms also contribute to its functional organization. The gut mycobiome can influence immune responses and has been implicated in inflammatory conditions, although its causal contribution remains less well defined than that of bacterial communities [98]. The virome, particularly bacteriophages, can regulate bacterial population dynamics and horizontal gene transfer, thereby shaping community structure and functional capacity [99]. Archaea, including methanogens, participate in metabolic processes such as hydrogen utilization and may influence fermentation efficiency and intestinal metabolic fluxes, with potential implications for host energy harvest [100]. These non‐bacterial components interact with bacterial communities and host systems, forming a multi‐kingdom ecosystem that contributes to the overall function and stability of the microbiome organ.

### Conceptualizing microbial metabolism as functional modules

A functional module represents an operational unit of metabolic activity within the microbiome organ, characterized by four key components: substrate inputs, microbial processes, metabolic outputs, and host effects. Substrate inputs can be exogenous, such as dietary components (e.g., fiber, amino acids), orally administered drugs, and environmental xenobiotics, or endogenous, such as host‐derived bile acids and mucus glycans [101, 102]. Microbial processes encompass the vast array of enzymatic reactions, including fermentation, hydrolysis, deconjugation, and reduction, carried out by specific members or consortia within the gut microbial community [94, 103]. These processes generate metabolic outputs, including a diverse suite of small molecules such as short‐chain fatty acids (SCFAs), secondary bile acids, and indole derivatives, which can act locally in the gut or be absorbed into systemic circulation [104]. Finally, these metabolites exert host effects by serving as energy substrates, acting as signaling molecules through interactions with host receptors (e.g., G‐protein‐coupled receptors, nuclear receptors, and the aryl hydrocarbon receptor (AhR)), or influencing host gene expression through epigenetic mechanisms, thereby integrating microbial activity with host physiological networks [105, 106, 107].

Viewing the microbiome through the lens of modularity provides a useful framework for dissecting the complex and often personalized nature of host–microbiome metabolic integration. This approach allows researchers to move beyond simple compositional surveys to a more functional understanding, linking the genetic potential of the microbiota (the metagenome) to its biochemical activity and subsequent impact on host phenotype [97, 108]. For instance, inter‐individual variations in drug response can be explained by differences in the presence or activity of specific microbial enzymes that metabolize a drug, a concept central to the emerging field of pharmacomicrobiomics [103, 109]. By identifying which microorganisms and genes constitute a particular functional module, such as the inactivation of the cardiac drug digoxin or the metabolism of the Parkinson's disease drug levodopa, it becomes possible to improve prediction of an individual's metabolic phenotype based on their microbiome composition and functional gene content [109, 110, 111]. This modular view helps represent the microbiome as a system of interconnected, functionally defined units whose activities can be modeled, predicted, and potentially manipulated for therapeutic benefit [96, 112].

### The carbohydrate fermentation module: Fueling the host and fortifying the gut barrier

The carbohydrate fermentation module is one of the most fundamental and impactful components of host–microbiome co‐metabolism. Its primary inputs are complex carbohydrates that escape digestion in the upper gastrointestinal tract, including dietary fibers such as inulin, galacto‐oligosaccharides (GOS), arabinoxylans, resistant starches [113, 114, 115], and host‐derived glycans such as mucin glycans. These substrates serve as a major energy source for a large proportion of the colonic microbiota, fueling an active anaerobic ecosystem [116]. The ability of gut microbes to utilize these polysaccharides is a testament to their extensive carbohydrate‐active enzymatic repertoire, which exceeds that of the human host [116].

The central microbial processes in this module are driven by a diverse array of carbohydrate‐active enzymes (CAZymes), including glycoside hydrolases, polysaccharide lyases, carbohydrate esterases, and associated binding modules, which cleave complex glycans, including fibers and starches, to release simpler sugars [117]. These liberated monosaccharides and oligosaccharides then enter bacterial carbohydrate fermentation and cross‐feeding pathways [118]. The outcome of this intensive metabolic activity is the production of a range of metabolites, most notably the SCFAs, primarily acetate, propionate, and butyrate, which represent a significant flux of carbon from the diet to the host [119]. The production of these SCFAs is a hallmark of saccharolytic fermentation. By lowering colonic pH, SCFAs can influence microbial community structure and may help limit the expansion of potentially pathogenic microorganisms [120].

The metabolic outputs of carbohydrate fermentation, particularly SCFAs, serve dual roles as both a vital energy source for the host and as critical signaling molecules [119, 121]. Butyrate is widely recognized as the preferred fuel for colonocytes, providing a major proportion of their energy requirements under physiological conditions and thereby directly supporting the health and integrity of the intestinal epithelium [121]. However, the energetic contributions of SCFAs extend beyond the colon. Butyrate and propionate can stimulate intestinal gluconeogenesis (IGN), a process that beneficially impacts systemic glucose and energy homeostasis [102]. Propionate acts as a direct substrate for IGN and also activates its gene expression through a gut‐brain neural circuit involving the fatty acid receptor FFAR3, while butyrate stimulates IGN gene expression via a cAMP‐dependent mechanism [102]. SCFAs are also absorbed from the gut lumen and can enter the portal/systemic circulation, where they are partly metabolized by the liver, with broader systemic effects on peripheral tissues, influencing lipid, carbohydrate, and protein metabolism and hepatic mitochondrial function [119, 122]. Beyond their role as fuel, SCFAs are potent signaling molecules that fortify the gut barrier. They can activate G‐protein‐coupled receptors, such as GPR43/FFAR2 and GPR109A/HCAR2, on intestinal epithelial and immune cells [123]. This signaling helps maintain epithelial barrier integrity, regulate epithelial cell proliferation and differentiation, and modulate local immune responses, including through GPR43‐ and GPR109A‐associated regulation of inflammasome pathways in specific contexts [123, 124]. Through these coordinated energetic and signaling functions, the carbohydrate fermentation module is indispensable for fueling host cellular processes and maintaining the critical barrier function of the gut.

The gut microbiome can influence epigenetic modifications in the host, affecting gene expression and noncoding RNA activity, which in turn may influence microbial composition and function, forming an epigenome–microbiome axis [125, 126]. SCFAs, particularly butyrate and propionate, can inhibit histone deacetylases (HDACs), influence DNA methylation‐related pathways, and provide acetyl‐CoA or related substrates that support histone acetylation [125, 126, 127]. These combined actions can promote a more permissive chromatin state, facilitating transcription of genes involved in immune function, glucose metabolism, and insulin sensitivity [126, 128].

Consistent with this, supplementation of germ‐free mice with SCFAs recapitulates chromatin remodeling, including histone acetylation and methylation patterns, as well as gene expression profiles observed in conventionally colonized animals [129]. In addition, SCFAs influence other histone modifications, such as crotonylation, partly through inhibition of HDAC activity [130]. Collectively, these findings support a mechanistic link between SCFAs, chromatin modification states, and transcriptional regulation.

### The amino acid processing module: Generating bioactive signals for host‐microbe communication

While carbohydrate fermentation is the dominant metabolic activity in the colon, the processing of amino acids by the gut microbiota constitutes another important functional module that generates bioactive molecules involved in host–microbe communication. The primary substrates for this module are dietary amino acids that escape absorption in the small intestine, as well as endogenous proteins from shed epithelial cells and mucus [116, 131]. Aromatic amino acids, particularly tryptophan, serve as key precursors for some of the most well‐characterized microbially derived signaling molecules [132]. Other substrates include branched‐chain amino acids (BCAAs), which are also subject to microbial modulation with significant consequences for host metabolism [133]. The balance between proteolytic and saccharolytic fermentation is a critical feature of the gut ecosystem, with shifts toward protein catabolism often associated with the production of potentially detrimental compounds alongside beneficial signaling molecules [116, 134].

Microbial metabolism of amino acids involves a diverse set of enzymatic reactions, including tryptophanase activity, decarboxylation, transamination, deamination, and reductive pathways [135, 136, 137]. Tryptophan, for example, can be metabolized by gut bacteria along several pathways, producing a variety of indole derivatives [106]. Tryptophanase activity leads to the production of indoles, whereas other microbial pathways can generate compounds such as indole‐3‐propionic acid (IPA), indole‐3‐acetic acid (IAA), and indole‐3‐carboxaldehyde (IAld) [135, 136, 137, 138]. The relative activity of these pathways can be influenced by host factors and microbial community composition, leading to distinct metabolic signatures associated with health or disease [136].

A major signaling axis originating from this module is the interaction between microbial tryptophan metabolites and the host's AhR, a ligand‐activated transcription factor expressed in various cell types, including immune and epithelial cells [139]. Indole derivatives such as IAA and IAld/indole‐3‐carboxaldehyde can act as AhR ligands, whereas IPA and other indole derivatives may signal through AhR‐dependent and AhR‐independent pathways [132, 139]. Upon receptor engagement, these microbial metabolites can trigger AhR‐mediated regulation of host gene expression, thereby influencing immune homeostasis and barrier function within and beyond the intestine [106]. For instance, AhR activation in astrocytes by tryptophan metabolites has been shown to limit central nervous system inflammation [132]. Within the gut, microbial indole–AhR signaling contributes to intestinal barrier function and mucosal immune regulation; separately, IPA has been shown to improve epithelial integrity and reduce inflammation in radiation‐induced injury models [135]. Tryptophan‐derived metabolites can also regulate gene expression through epigenetic mechanisms. For example, IPA can upregulate lysine demethylase 6B (KDM6B) to reduce H3K27me3 histone methylation at target promoters, enhancing transcription of mitochondrial regulatory genes [128]. This was associated with alleviation of gut inflammation, increased mitochondrial bioenergetics, and improved osteoblast function in an animal model of obesity‐induced osteopenia [140].

The gut microbiota can modulate systemic BCAA homeostasis, and alterations in microbial BCAA metabolism have been linked to metabolic and aging‐related phenotypes [132, 140]. For instance, excess circulating BCAAs, which may partly reflect altered microbial BCAA metabolism, can contribute to diabetic cardiomyopathy by altering signaling pathways in the liver and heart [133]. Interestingly, recent evidence indicates that BCAAs can indirectly influence histone propionylation by altering intracellular levels of propionyl‐CoA. Elevated propionyl‐CoA was associated with changes in cardiac gene expression [141]. In particular, extracellular matrix (ECM) genes and cluster‐of‐differentiation (CD) markers expressed in infiltrating immune cells were upregulated and linked to pressure overload–induced cardiac remodeling, providing a potential mechanistic basis for the role of BCAAs in cardiomyopathy. Specifically, microbial modulation of BCAA levels can influence key host metabolic signaling pathways, including the mechanistic target of rapamycin (mTOR) and fibroblast growth factor 21 (FGF21), which are central regulators of growth, metabolism, and insulin sensitivity [133, 142]. These findings suggest that the amino acid processing module generates bioactive signals that can integrate microbial metabolism with systemic host physiology and immune function. Moreover, BCAA administration has been reported to modulate epigenetic regulatory enzymes, mainly HDACs and DNA methyltransferases (DNMTs), in brain tissue in an animal model of maple syrup urine disease, highlighting a possible extra‐intestinal epigenetic effect [143].

### The bile acid biotransformation module: A key hub for microbe‐nuclear receptor crosstalk

The biotransformation of bile acids represents a unique functional module where the major substrates are host‐derived, creating an intimate metabolic circuit between the liver and the gut microbiota. The inputs for this module are primary bile acids, principally cholic acid (CA) and chenodeoxycholic acid (CDCA), which are synthesized from cholesterol in the host liver, conjugated to taurine or glycine, and secreted into the intestine to aid in lipid digestion [144]. While the majority of conjugated primary bile acids are reabsorbed in the ileum and returned to the liver via the enterohepatic circulation, a significant portion escapes into the colon, where they become substrates for extensive microbial modification [144].

In the colon, gut bacteria deploy a series of enzymatic reactions to remodel the structure of these host‐derived molecules. The major microbial processes include deconjugation, dehydroxylation, oxidation, epimerization, and other modifications. Deconjugation, catalyzed by microbial bile salt hydrolases (BSHs), removes the taurine or glycine moiety, a critical gateway step for subsequent transformations [145, 146]. Following deconjugation, a restricted subset of anaerobic bacteria, mainly within *Clostridia*, can perform 7α‐dehydroxylation, which converts primary bile acids into secondary bile acids [147, 148]. For example, CA is converted to deoxycholic acid (DCA), and CDCA is transformed into lithocholic acid (LCA) [147, 149]. This microbial activity substantially alters the composition and physicochemical properties of the bile acid pool, generating a diverse array of signaling molecules with potent biological activities [105, 144].

The metabolic outputs of this module, the secondary bile acids, are potent signaling molecules that function as ligands for several host nuclear receptors and G‐protein‐coupled receptors, thereby orchestrating a complex network of microbe‐host crosstalk. Secondary bile acids such as DCA and LCA can engage several bile acid‐sensitive receptors, including TGR5/GPBAR1, VDR, PXR, and, depending on bile acid species and concentration, FXR [105]. Activation of these receptors in intestinal epithelial cells (IECs), hepatocytes, cholangiocytes, enteroendocrine cells, and immune cells can trigger downstream signaling cascades that regulate a wide range of host physiological processes [105]. For instance, activation of ileal FXR induces FGF19/FGF15‐dependent feedback loops that suppress hepatic bile acid synthesis, while TGR5 activation can influence gut motility and GLP‐1 secretion, impacting glucose homeostasis [105].

In addition to receptor‐mediated signaling, the functional impact of bile acids is critically modulated by their conjugation state. Host‐mediated glycine and taurine conjugation enhance bile acid solubility and facilitate enterohepatic circulation, whereas microbial BSHs deconjugate these species, altering their reabsorption, antimicrobial activity, and receptor affinity. Furthermore, host sulfation, particularly of hydrophobic secondary bile acids such as LCA, serves as an important detoxification pathway that promotes excretion and limits cytotoxicity. These transformations also intersect with xenobiotic metabolism, as bile acids can compete with, or regulate, drug‐metabolizing enzymes and transporters, thereby influencing drug bioavailability and toxicity [150, 151].

Through this integrated network of biotransformation and signaling, the bile acid module functions as a critical hub in which microbial activity not only generates bioactive ligands but also modulates their chemical form, systemic distribution, and interaction with host metabolic and detoxification pathways. Consequently, this module contributes to broad regulation of host lipid, glucose, and energy homeostasis, as well as inflammation, immune responses, and drug metabolism [150, 151].

### The choline catabolism module: A model of obligate host–microbe co‐metabolism

The choline catabolism module provides a classic example of an obligate metabolic pathway that spans the microbial and host kingdoms, producing systemic co‐metabolites with implications for host health, particularly cardiometabolic and renal disease. The primary substrates for this module are trimethylamine (TMA)‐containing nutrients derived from the diet, including choline, phosphatidylcholine (lecithin), and l‐carnitine, which are abundant in foods such as red meat, eggs, and fish [152, 153, 154, 155]. These compounds can be used by gut microbes that possess specific TMA‐generating enzymatic machinery required to initiate their catabolism [154].

The central microbial process in this module is the cleavage of the C–N bond in choline and related compounds by a class of enzymes known as TMA‐lyases, such as CutC, leading to the production of volatile TMA as a metabolic output [156, 157]. This conversion is generally considered a gut microbiota‐dependent function, as the host does not encode the microbial enzymes required to generate TMA from these dietary precursors [154, 158]. The resulting TMA is a small, volatile molecule that is readily absorbed from the intestinal lumen into the portal circulation, from where it is transported to the liver [154, 159].

The trimethylamine *N*‐oxide (TMAO) axis represents a sequential co‐metabolic pathway that bridges microbial and host biochemistry [154, 160]. Microbial metabolism of dietary substrates such as choline, phosphatidylcholine, and carnitine generates TMA, which is subsequently absorbed and oxidized in the liver by flavin‐containing monooxygenase 3 (FMO3) to form TMAO [153, 154]. TMAO is a stable, water‐soluble molecule that circulates systemically and is primarily excreted via the kidneys, making circulating concentrations sensitive to renal function [155, 161].

Elevated circulating levels of TMAO have been associated with an increased risk of adverse health outcomes, including atherosclerotic cardiovascular disease (CVD), thrombosis, chronic kidney disease (CKD), and mortality [161, 162]. Mechanistic studies, mainly in experimental models, suggest that TMAO may influence cholesterol metabolism, platelet reactivity, and vascular inflammation, supporting a potential contributory role in cardiometabolic disease. However, the interpretation of these associations remains complex and context‐dependent. Notably, TMAO is abundant in marine fish, yet fish consumption is consistently associated with reduced cardiovascular risk, indicating that TMAO is unlikely to function as a uniformly deleterious metabolite. In addition, circulating TMAO levels are strongly influenced by renal function, dietary patterns, and microbiome composition, raising the possibility of confounding and reverse causation, particularly in CKD.

Emerging evidence further suggests that TMAO should not be interpreted solely as a harmful metabolite: beyond its association with cardiometabolic and neurological disease, it has been described in marine and other non‐mammalian systems as an osmolyte with protein‐stabilizing, chaperone‐like properties, and its biological effects may differ across metabolic and disease contexts. Collectively, these observations support a more nuanced interpretation of the TMA–TMAO axis, in which TMAO serves not only as a potential effector molecule but also as a context‐dependent metabolite reflecting interactions among diet, microbiome composition, renal function, and host physiology [154, 163].

### The xenobiotic and drug metabolism module: Modulating host pharmacology and toxicology

**FIGURE 3:**
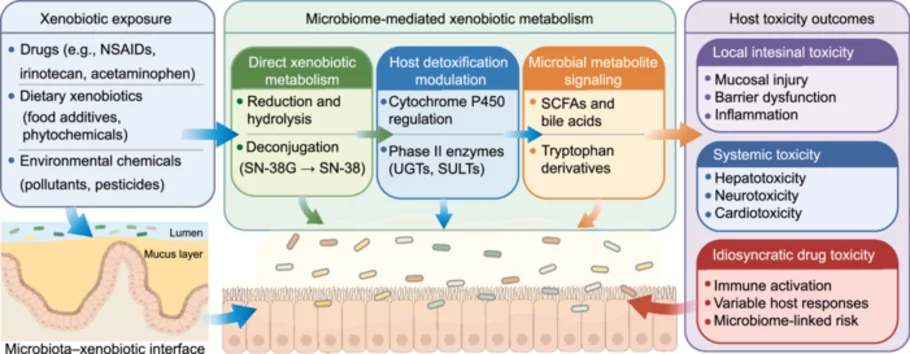
Microbiome‐mediated xenobiotic transformation and host drug‐response variability. The gut microbiome transforms xenobiotics through direct microbial enzymatic activities and indirect modulation of host metabolic and immune pathways, thereby influencing drug exposure, efficacy, and toxicity. Microbial biotransformation can generate detoxified, activated, inactivated, or reactivated metabolites in selected contexts, whereas microbiome‐dependent regulation of host enzymes, transporters, barrier function, and immune responses further shapes susceptibility to adverse effects. Together, these interactions contribute to inter‐individual variability in drug response and selected toxicity phenotypes. AhR, aryl hydrocarbon receptor; CYPs, cytochrome P450 enzymes; FXR, farnesoid X receptor; GPCRs, G protein‐coupled receptors; NSAIDs, non‐steroidal anti‐inflammatory drugs; PXR, pregnane X receptor; SCFAs, short‐chain fatty acids; SULTs, sulfotransferases; TGR5, Takeda G protein‐coupled receptor 5; UGTs, UDP‐glucuronosyltransferases.

The gut microbiota is an important interface between the host and xenobiotic exposure and functions as a metabolically active component of the gut ecosystem that can influence the pharmacology and toxicology of a wide range of compounds (Figure 3) [95]. The substrates for this module are highly diverse, encompassing orally administered drugs, dietary compounds such as polyphenols and phytoestrogens, and environmental chemicals including contaminants and food additives [101, 164, 165]. The extensive enzymatic capacity of the gut microbiome enables a broad spectrum of chemical transformations, contributing to variability in drug response and toxicity across individuals [103, 109, 166].

The direct processing of xenobiotics by microbiota involves enzymatic reactions that often differ from those of the host liver, including reduction, hydrolysis, and deglycosylation [94, 167]. These transformations can have clinically relevant consequences for the host. Variability in microbiome composition contributes to heterogeneous drug responses by modulating both drug metabolism and downstream host pathways. For example, microbial enzymes can activate drugs, as seen in the cleavage of the azo bond in sulfasalazine to release its active anti‐inflammatory component, 5‐aminosalicylic acid [168]. Conversely, microbial metabolism can result in drug inactivation or reduced bioavailability; a well‐characterized case is the reduction of the cardiac glycoside digoxin by *Eggerthella lenta*, which compromises therapeutic efficacy [110, 169]. Microbial activity can also lead to toxification. A clinically relevant example is the reactivation of the glucuronidated irinotecan metabolite SN‐38G to SN‐38 by microbial β‐glucuronidases, contributing to irinotecan‐induced gastrointestinal toxicity [170]. Similarly, microbial esterases have been reported to hydrolyze aspirin into salicylic acid in experimental settings, which may contribute to intestinal injury [171]. Mapping these metabolic activities to specific microbial genes and taxa remains a major objective for improving predictions of microbiome‐dependent variation in drug responses [103].

Beyond direct chemical transformation, the gut microbiome indirectly modulates host drug metabolism by regulating the expression and activity of host metabolic machinery. This regulation is mediated in part by microbial metabolites that act as signaling molecules [172]. These metabolites can reach host tissues through gut–liver communication and influence the expression of drug‐metabolizing enzymes and transporters in the liver and intestine [173]. This process is often mediated through nuclear receptors such as the pregnane X receptor (PXR) and the constitutive androstane receptor (CAR), which regulate xenobiotic response pathways [174]. Microbial metabolites, including secondary bile acids, indole derivatives, and SCFAs, can modulate PXR‐, CAR‐, and other nuclear receptor‐associated pathways, thereby altering the expression of phase I enzymes such as cytochrome P450s, phase II enzymes, and transporters including P‐glycoprotein (ABCB1/P‐gp) [175, 176]. For example, microbiome‐dependent regulation of ABCB1 has been implicated in altered pharmacokinetics of drugs such as tacrolimus [176]. This gut–liver signaling axis highlights how microbial activity can systemically influence host drug disposition [175].

Pharmacological agents can also reshape the composition and function of the gut microbiome, forming a reciprocal interaction central to pharmacomicrobiomics. For example, metformin has been reported to alter specific microbial taxa, including reducing *Bacteroides fragilis* in some cohorts, thereby contributing to its therapeutic effects through modulation of bile acid metabolism and intestinal FXR signaling [177]. Similarly, aspirin can alter microbial community structure and influence intestinal homeostasis through gut microbiota–bile acid signaling and effects on epithelial and stem cell dynamics [178]. Gut microbes may also modulate aspirin bioavailability and chemopreventive efficacy, whereas aspirin‐associated microbial changes have been proposed to contribute to anti‐atherosclerotic effects [179, 180]. Statins have also been associated with shifts in gut microbial composition, including enrichment of taxa such as *Limosilactobacillus reuteri* in some studies, which may contribute to immunomodulatory effects [181].

Together, these findings indicate that drug–microbiome interactions operate as a dynamic system in which microbial metabolism influences drug efficacy while pharmacological agents reshape microbial ecology. This interplay adds substantial complexity to predicting therapeutic outcomes and underscores the importance of incorporating microbiome context into pharmacological and clinical decision‐making.

### Section summary

In summary, conceptualizing the gut microbiome as a constellation of functional metabolic modules provides a useful framework for understanding how microbial biochemical activities are systematically coupled to host physiology. Across modules including carbohydrate fermentation, amino acid processing, bile acid biotransformation, choline catabolism, and xenobiotic metabolism, the microbiome can be viewed as an integrated biochemical interface that transforms dietary, endogenous, and environmental substrates into a diverse repertoire of bioactive metabolites. These metabolites engage host receptors, signaling pathways, and metabolic networks, thereby contributing to the regulation of energy homeostasis, immune regulation, pharmacological responses, and disease susceptibility. Importantly, the modular perspective highlights both the specificity and interconnectedness of microbial functions, emphasizing that host–microbiome interactions emerge from coordinated metabolic networks rather than isolated pathways. Future advances integrating multi‐omics profiling, causal mechanistic studies, and predictive modeling will be essential to map these modules with greater precision, ultimately supporting the development of targeted manipulation of microbiome functions for therapeutic and precision medicine applications.

## THE MICROBIOME ORGAN AS A METABOLIC AND ENDOCRINE‐LIKE SIGNALING SYSTEM

### Overview

**FIGURE 4:**
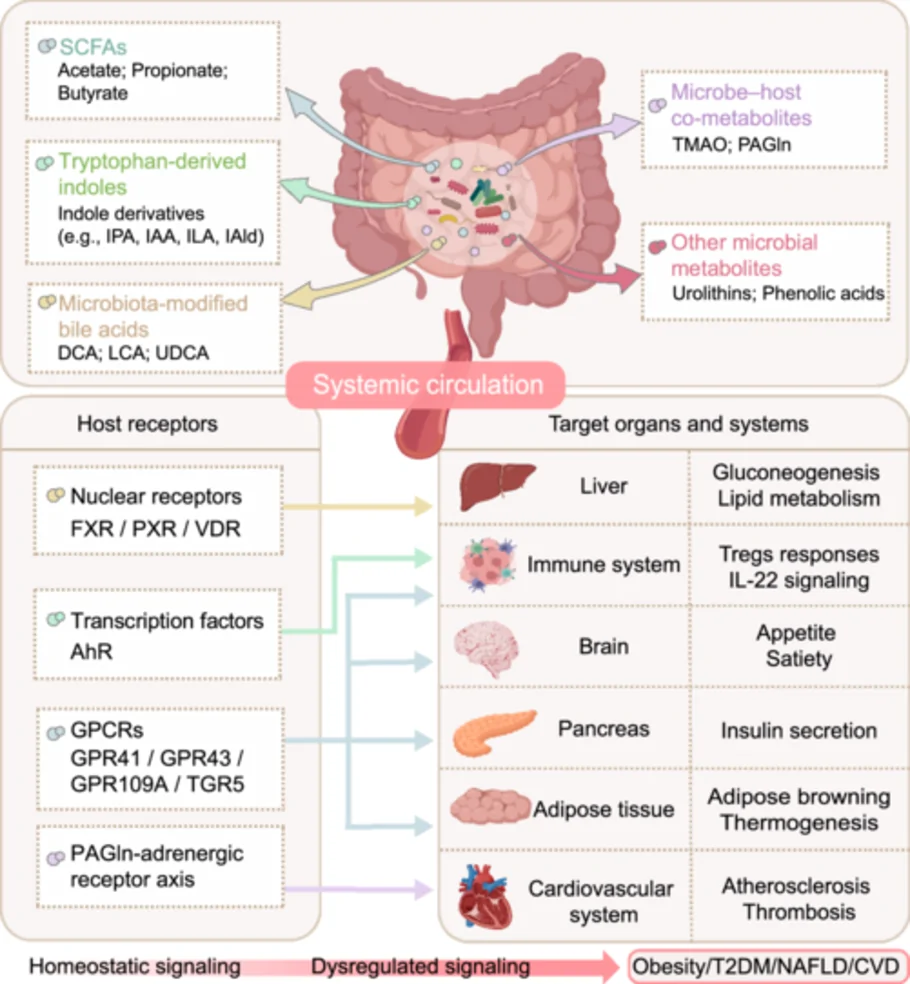
The gut microbiome as an endocrine‐like metabolic signaling system. The gut microbiome functions as an endocrine‐like metabolic signaling system by producing and modifying diverse bioactive metabolites, including SCFAs, tryptophan‐derived indoles, microbiota‐modified bile acids, and host–microbial co‐metabolites. These microbial and co‐metabolic signals enter systemic circulation and engage host receptors and signaling pathways, including GPCRs, nuclear receptors, AhR/PXR‐associated pathways, and PAGln–adrenergic receptor signaling, across distant organs. Through these pathways, the microbiome modulates energy balance, glucose and lipid metabolism, immune homeostasis, appetite and satiety, incretin and insulin‐related signaling, thermogenesis, and cardiovascular function. Dysregulated microbiome‐derived signaling has been associated with, and may contribute to, metabolic and cardiovascular disease‐related phenotypes, including T2DM, MASLD, CVD, and CKD. AhR, aryl hydrocarbon receptor; CKD, chronic kidney disease; CVD, cardiovascular disease; DCA, deoxycholic acid; FXR, farnesoid X receptor; GPCRs, G protein‐coupled receptors; FFAR3, free fatty acid receptor 3; FFAR2, free fatty acid receptor 2; HCAR2, hydroxycarboxylic acid receptor 2; IAA, indole‐3‐acetic acid; IAld, indole‐3‐carboxaldehyde; ILA, indole‐3‐lactic acid; IL‐22, interleukin‐22; IPA, indole‐3‐propionic acid; LCA, lithocholic acid; MASLD, metabolic dysfunction‐associated steatotic liver disease; PAGln, phenylacetylglutamine; PXR, pregnane X receptor; SCFAs, short‐chain fatty acids; T2DM, type 2 diabetes mellitus; TGR5/GPBAR1, Takeda G protein‐coupled receptor 5/G protein‐coupled bile acid receptor 1; TMAO, trimethylamine N‐oxide; Tregs, regulatory T cells; UDCA, ursodeoxycholic acid; VDR, vitamin D receptor.

The gut microbiota is increasingly recognized as a functional, integrated system with endocrine‐like metabolic signaling properties (Figure 4) [9, 182]. This virtual organ communicates extensively with the host, modulating multiple physiological processes through a sophisticated network of signaling molecules [183, 184]. Central to this paradigm is the microbiome's substantial capacity to transform dietary components and host‐derived molecules, such as bile acids, into a diverse array of circulating, receptor‐active metabolites [184]. These bioactive compounds, including SCFAs, tryptophan derivatives, and secondary bile acids, can function as endocrine‐like signaling molecules that interact with host receptors locally and systemically, thereby influencing host metabolism, immunity, and potentially neurobehavioral functions [9, 182, 183, 184, 185]. The crosstalk established through these metabolite–receptor axes contributes to metabolic homeostasis. This section aims to delineate the key microbial metabolites that function as endocrine signals, identify the host receptors and pathways that sense these signals, and explore how this microbiome–endocrine axis systemically regulates energy, glucose, and lipid homeostasis. Furthermore, it will examine how dysregulation of this complex signaling network may contribute to the pathophysiology of prevalent metabolic and liver‐related diseases, including obesity, type 2 diabetes, CVD, and metabolic dysfunction‐associated steatotic liver disease (MASLD; formerly NAFLD) and metabolic dysfunction‐associated steatohepatitis (MASH; formerly NASH), and discuss emerging therapeutic strategies designed to target this critical interface [182, 183, 186].

### Microbial metabolites as endocrine signaling molecules

The gut microbiota produces a vast chemical repertoire of small molecules that enter systemic circulation and act as signaling messengers, thereby exerting endocrine‐like, paracrine, or local effects. Among these, SCFAs, generated from the fermentation of dietary fibers, are important mediators of host physiology [187]. Acetate, the most abundant SCFA in the colon and in the circulation, participates in lipid metabolism and appetite regulation in a context‐dependent manner [188]. Propionate contributes to glucose homeostasis by serving as a substrate and signaling molecule, influencing IGN, hepatic substrate flux, GLP‐1 secretion, and, in some contexts, sympathetic nervous system activity [189]. Butyrate, while serving as a major energy source for colonocytes, exerts systemic effects by influencing the function and differentiation of immune cells, including effects on regulatory T cells (Tregs) and, in selected experimental immune contexts, T follicular helper cell subsets, such as Tfh13 cells, thereby shaping both mucosal and systemic immunity [190].

Microbial metabolism of dietary tryptophan further expands this signaling capacity [185]. Indole derivatives, including IAA and ILA, can act as AhR ligands, whereas IPA may signal through AhR‐dependent, PXR‐dependent, antioxidant, and other pathways, linking microbial metabolism to host immune and barrier functions [188, 191]. In parallel, shifts in host tryptophan metabolism toward the kynurenine pathway are linked to inflammation and immune‐metabolic regulation, whereas bacterial tryptamine can influence gut secretion and transit through epithelial or neuronal 5‐HT4 receptor signaling [192, 193].

Another critical endocrine‐like function of the gut microbiota is the biotransformation of primary bile acids, synthesized in the liver, into secondary or microbially modified bile acids such as DCA, LCA, and, in some contexts, ursodeoxycholic acid (UDCA) [193]. These modified bile acids act as signaling molecules that regulate bile acid synthesis and enterohepatic circulation through the gut–liver FXR–FGF19/FGF15 axis, while bile acid signaling more broadly modulates systemic lipid and glucose homeostasis through nuclear and membrane‐bound receptors, including FXR and TGR5 [194, 195]. This microbial modification of the bile acid pool contributes to metabolic control [195].

Furthermore, a growing list of other microbial and co‐metabolites exerts systemic endocrine‐like effects. Phenylacetylglutamine (PAGln), a microbial–host co‐metabolite generated from phenylalanine metabolism, can signal through adrenergic receptors and has been linked to adverse cardiovascular events [196]. Urolithins, which are microbiota‐dependent metabolites derived from dietary polyphenols, have been reported to influence gut barrier function and inflammation through AhR‐dependent and AhR‐independent pathways [197]. Together, this diverse array of metabolites supports the view of microbiota as an endocrine‐like signaling system that can influence host health and disease.

### Host receptors and pathways sensing microbial signals

The host expresses a diverse sensory apparatus that detects and responds to chemical signals produced by the gut microbiota, integrating these inputs to modulate systemic physiology. This machinery includes a variety of receptors expressed on the cell surface and within the cell nucleus of enteroendocrine cells, immune cells, and epithelial cells lining the gut [198]. G‐protein‐coupled receptors (GPCRs) serve as key cell surface sensors for a wide range of microbial metabolites. The SCFA receptors GPR41/FFAR3, GPR43/FFAR2, and GPR109A/HCAR2 are important examples, mediating the effects of acetate, propionate, and butyrate on intestinal epithelial and immune‐cell functions [198, 199]. The functional effects can be complex; for instance, GPR43 activation has been linked to the regulation and resolution of inflammatory responses in several disease models [199]. Similarly, the bile acid receptor TGR5/GPBAR1 contributes to GLP‐1 release from intestinal l‐cells and has been implicated, particularly in experimental models, in regulating energy expenditure and thermogenic programs [193]. More recently identified pathways include the sensing of metabolites such as PAGln and tyramine through adrenergic receptor‐related mechanisms, which may link microbial metabolism to cardiovascular function and intestinal epithelial or stem‐cell biology in specific contexts [196, 200].

In addition to surface receptors, microbial metabolites can enter host cells or signal to intracellular receptors to modulate metabolic pathways, primarily nuclear receptors. FXR is a central receptor in the gut–liver axis, where it senses changes in the composition of microbially modified bile acids to regulate their synthesis, transport, and enterohepatic circulation, thereby contributing to the regulation of systemic lipid and glucose metabolism [194]. Inhibition of intestinal FXR signaling, through microbiota‐dependent changes in antagonist bile acids or pharmacological inhibition, has been shown in specific experimental and clinical contexts to improve features of diet‐induced obesity and metabolic dysfunction, highlighting its role as a metabolic checkpoint [201, 202]. Other nuclear receptors, such as the PXR and the VDR, also function as sensors for microbial metabolites, including indole derivatives and secondary bile acids like LCA [203, 204]. Activation of these receptors contributes to the regulation of gut barrier function, inflammation, and xenobiotic metabolism, further integrating microbial signals with host defense and detoxification pathways [135].

A central integrative node is AhR, which detects selected tryptophan‐derived microbial metabolites [188]. Activation of AhR in immune populations, including innate lymphoid cells (ILC3s), promotes interleukin‐22 (IL‐22) production and reinforces epithelial barrier integrity [205]. Beyond the gut, AhR signaling contributes to systemic immune regulation, including effects on regulatory B cells, positioning it as an important mediator of host–microbiota communication in health and disease [206]. Recent experimental evidence further extends AhR‐mediated host–microbiota signaling to the gut–brain axis, where microbiota‐derived indole has been reported to promote adult hippocampal neurogenesis in an AhR‐dependent and ligand‐specific manner, linking microbial tryptophan metabolism to central nervous system function [207].

### Systemic metabolic regulation by the microbiome–endocrine axis

The integrated signaling network formed by microbial metabolites and host receptors exerts substantial but context‐dependent influence over systemic energy homeostasis, balancing energy intake, harvest, and expenditure [208]. In experimental models, the gut microbiome can influence energy balance by enhancing the host's capacity to extract calories from otherwise indigestible dietary polysaccharides, a trait that can be transmissible by microbiota transfer and has been associated with obesity‐related phenotypes [209]. Beyond simple energy extraction, microbial metabolites function as critical endocrine signals that regulate appetite and satiety. SCFAs and other microbial products can stimulate the secretion of gut hormones such as GLP‐1 and peptide YY (PYY) from enteroendocrine l‐cells, which act through gut–brain pathways to promote satiety and regulate food intake [210]. This signaling axis is one mechanism through which diet–microbiota interactions influence feeding behavior [210]. The microbiome–endocrine axis also regulates energy expenditure by activating thermogenesis in brown adipose tissue (BAT) and promoting the browning of white adipose tissue (WAT) [211]. For instance, in mouse models, cold exposure leads to shifts in the microbiota that promote WAT browning and increase energy expenditure, effects that can be transferable via fecal microbiota transplantation (FMT) [212]. Secondary bile acid signaling through TGR5 and SCFA‐related pathways have both been implicated in activating these thermogenic programs, representing a pathway to dissipate excess energy as heat [213].

The regulation of glucose and insulin homeostasis is another cornerstone of the microbiome's metabolic influence [214]. Microbiota‐associated incretin signaling is one mechanism relevant to glucose control, wherein microbial metabolites such as SCFAs and bile acids can stimulate GLP‐1 release and thereby support glucose‐dependent insulin secretion from pancreatic β‐cells [215]. This may be one mechanism by which fermentable fibers and selected probiotics influence insulin secretion and insulin sensitivity [189, 216]. Furthermore, microbial metabolites can directly modulate hepatic glucose metabolism. Propionate, for example, can modulate glucose homeostasis through IGN, gut–brain signaling, incretin pathways, and hepatic substrate metabolism [187]. Collectively, these actions can contribute to improved peripheral and hepatic insulin sensitivity in specific contexts, supporting the gut microbiome as one regulator of glycemic control [217]. The microbiome also engages in extensive crosstalk with lipid metabolism and adiposity. SCFA signaling through GPR43 in adipocytes has been shown to suppress insulin‐mediated fat accumulation, thereby limiting adipose tissue expansion [218]. In the liver, the bile acid–FXR axis is an important regulator of hepatic de novo lipogenesis and cholesterol metabolism, providing a direct link between microbial bile acid modifications and hepatic fat content [194]. Microbial metabolites also modulate adipose tissue inflammation, a key feature of obesity‐associated insulin resistance [219]. Finally, by reinforcing gut barrier function through mechanisms involving IL‐22 signaling, the microbiome–endocrine‐like axis may help restrict the translocation of pro‐inflammatory microbial products such as lipopolysaccharide (LPS) into the circulation, thereby mitigating metabolic endotoxemia and chronic low‐grade inflammation [191].

### Dysregulation of the microbiome–endocrine axis in disease

Disruption of communication within the microbiome–endocrine‐like axis is a feature of metabolic diseases and may contribute to their pathophysiology [183]. In obesity and the metabolic syndrome, the gut microbiota is characterized by a state of disrupted signaling, featuring altered profiles of key metabolites [220]. Obesity has been associated with altered microbiota composition and function, including changes in diversity, energy‐harvest pathways, and metabolite profiles, although simple phylum‐level signatures have not been consistent across human studies [221]. In metabolic disease states, dysbiosis can include reduced abundance or activity of butyrate‐producing bacteria, a feature relevant to gut barrier function and immune regulation [183, 222]. Concurrently, bile acid metabolism is often dysregulated, with altered ratios of FXR agonists and antagonists contributing to impaired lipid and glucose homeostasis and promoting hepatic steatosis [223]. This disruption in chemical signaling, combined with increased gut permeability, may facilitate metabolic endotoxemia and contribute to the chronic low‐grade inflammation associated with insulin resistance and broader metabolic dysfunction [224].

In type 2 diabetes mellitus (T2DM), these disruptions may be more pronounced and may contribute to impaired glucose metabolism [225]. Metagenome‐wide association studies have identified distinct gut microbial signatures in individuals with T2DM, characterized by reduced butyrate‐producing capacity and enrichment of selected opportunistic or pro‐inflammatory taxa [222]. This functional deficit may weaken SCFA‐related metabolic signaling, including pathways linked to GLP‐1 secretion and postprandial glucose control [189, 226]. Furthermore, accumulation of selected microbial or co‐metabolites has been associated with insulin resistance and, in experimental settings, may contribute mechanistically [227]. The gut–liver axis is particularly relevant to the progression of MASLD and MASH [228]. In these conditions, dysfunctional FXR and TGR5 signaling, driven by altered microbial bile acid profiles, promotes hepatic fat accumulation, inflammation, and fibrosis [194, 229]. The compromised gut barrier also allows for increased translocation of microbial products, such as endotoxin and microbially derived ethanol or ethanol‐related metabolites, to the liver, fueling hepatic inflammation and disease progression [186]. The influence of the microbiome–endocrine axis extends beyond metabolic disorders to CVD, representing a potential contributory factor that may interact with traditional cardiovascular risk factors [222]. Emerging evidence connects microbial regulation of SCFAs and bile acids to the control of blood pressure, further solidifying the role of this axis in overall cardiovascular health [230].

### Therapeutic targeting of the microbiome–endocrine axis

The central role of the microbiome–endocrine axis in metabolic health and disease presents multiple potential therapeutic targets. Modulating this axis through dietary interventions is a primary strategy, leveraging the microbiota's capacity to transform nutritional inputs into bioactive signals [231]. Prebiotics, defined as substrates selectively utilized by host microorganisms that confer a health benefit, have been shown to improve metabolic outcomes. For instance, supplementation with inulin or GOS can increase the abundance of selected taxa such as *Bifidobacterium* and *Akkermansia*, enhance SCFA production, improve insulin sensitivity, and protect against diet‐induced obesity [189, 232]. Probiotics, the administration of live beneficial microorganisms, represent another approach. Supplementation with pasteurized *Akkermansia muciniphila* has shown promising metabolic effects, including improved insulin sensitivity and selected inflammatory or metabolic markers, in early human proof‐of‐concept trials involving overweight or obese insulin‐resistant individuals [233]. Functional foods rich in polyphenols, such as cranberry extract, have shown metabolic benefits in animal models, partly associated with changes in taxa such as *Akkermansia* spp. [234].

For more profound dysbiosis, FMT offers an experimental approach to modifying microbial community structure and associated metabolic functions. An early clinical study reported that transplantation of microbiota from lean, healthy donors into individuals with metabolic syndrome transiently improved insulin sensitivity in some recipients, an effect associated with changes in intestinal microbiota, including butyrate‐producing species [235]. However, the efficacy of FMT can be variable, and there are concerns about unintended long‐term consequences, highlighting the need for more refined and standardized microbiome‐based interventions [236]. Pharmacological interventions are also being developed to directly target components of the microbiome–endocrine axis. Metformin, a widely used therapy for T2DM, is now understood to exert some of its glucoregulatory effects partly by altering the gut microbiota, leading to changes in bile acid metabolism, including increased GUDCA in some studies, inhibition of intestinal FXR signaling, and improved glucose homeostasis [177]. In parallel, drug development has increasingly focused on bile–acid receptor signaling as a therapeutic target. FXR agonists and dual FXR/TGR5 ligands have been investigated for MASH and other metabolic diseases, although clinical efficacy, safety, tolerability, and regulatory outcomes have been mixed [237, 238]. A future direction in this field may lie in engineered live biotherapeutic products (LBPs) or synthetic biotic medicines designed to produce therapeutic metabolites, consume harmful metabolites, or modulate defined host–microbe pathways, thereby offering a highly targeted and controlled method for manipulating the microbiome–endocrine axis for therapeutic gain [239].

### Section summary

This section has delineated the gut microbiome's function as a dynamic endocrine‐like signaling system, a concept that helps reframe our understanding of host metabolism. The continuous dialog between the host and its microbial residents is orchestrated through a diverse array of bioactive metabolites, including SCFAs, tryptophan derivatives, and secondary bile acids, which act as endocrine‐like signaling molecules. These microbial signals are sensed by a network of host receptors, including GPCRs such as GPR43/FFAR2 and TGR5/GPBAR1, nuclear receptors such as FXR and PXR, and the pivotal transcription factor AhR. This microbiome–endocrine‐like axis contributes to the regulation of systemic energy balance, glucose and lipid homeostasis, and low‐grade inflammation by modulating appetite, incretin secretion, thermogenesis, and immune cell function. The evidence presented supports a contributory role for dysregulation of this axis in metabolic and cardiometabolic disease phenotypes, including obesity, type 2 diabetes, MASLD, and CVD, where altered metabolite profiles and disrupted signaling contribute to energy imbalance, insulin resistance, and chronic inflammation [183, 240].

Looking to the future, the therapeutic potential of targeting the microbiome–endocrine‐like axis is substantial, but significant challenges remain in translating preclinical findings into effective human therapies. The high degree of inter‐individual variability in gut microbiota composition and function necessitates the development of personalized interventions, moving beyond one‐size‐fits‐all approaches [241]. Future research must focus on elucidating the precise mechanisms linking specific microbial taxa and their metabolic outputs to host physiological responses in human populations. This will require integrating multi‐omics data with well‐controlled clinical intervention studies to establish causality and identify robust biomarkers for predicting therapeutic response [241]. The discovery of novel microbial metabolites and their cognate or responsive host receptors continues to open new avenues for drug development, offering the potential for targeted pharmacological agents that can mimic or enhance the beneficial effects of the microbiota [228]. Ultimately, a deeper understanding of this complex interplay may support innovative strategies, from precision nutrition and next‐generation probiotics (NGPs) to engineered biotherapeutics, designed to restore metabolic homeostasis by recalibrating the crucial conversation between the host and its microbial endocrine organ.

## THE MICROBIOME AS A REGULATOR OF IMMUNE HOMEOSTASIS

### Overview

**FIGURE 5:**
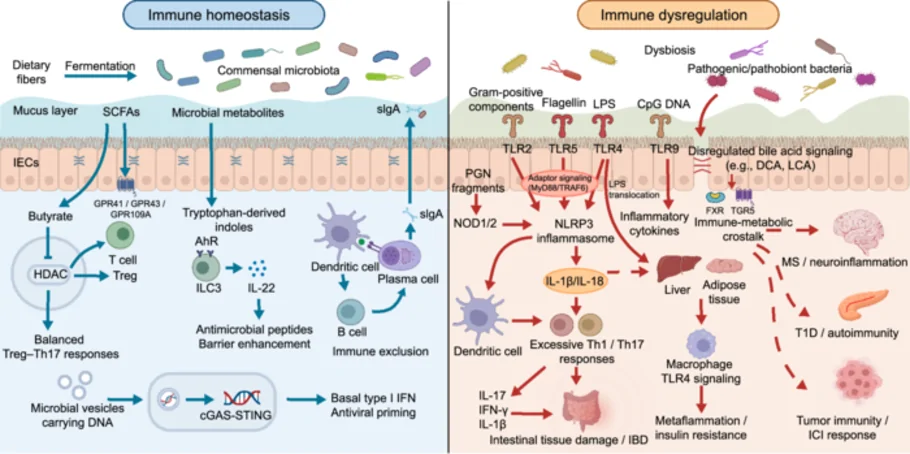
The gut microbiome as a multi‐layer regulator of host immunity. The gut microbiome regulates host immunity through a multi‐layered network involving microbial metabolites, extracellular vesicles, microbial structural components, epithelial barrier signals, and mucosal immune pathways. Microbial metabolites such as SCFAs and tryptophan‐derived indoles support epithelial barrier function, immune tolerance, and balanced Treg–Th17 responses, whereas sIgA contributes to immune exclusion and spatial containment of commensal microbes. Microbial products and vesicles are sensed by host pattern‐recognition receptors and signaling pathways, including TLRs, NOD‐like receptors, and cytosolic DNA‐sensing pathways such as cGAS–STING, with downstream adaptor and signaling modules including MyD88, TRIF, TRAF6, and RIPK2 depending on receptor context. Under dysbiosis, barrier disruption, or pathobiont expansion, excessive PRR activation, inflammasome priming, altered bile acid signaling, and microbial translocation may promote mucosal inflammation, systemic immune activation, metaflammation, autoimmunity‐related phenotypes, and altered tumor immunity or ICI response. AhR, aryl hydrocarbon receptor; cGAS–STING, cyclic GMP‐AMP synthase–stimulator of interferon genes; CpG, cytosine‐phosphate‐guanine DNA motifs; FXR, farnesoid X receptor; HDAC, histone deacetylase; IBD, inflammatory bowel disease; ICI, immune checkpoint inhibitor; IECs, intestinal epithelial cells; IFN, interferon; IL, interleukin; ILC3, group 3 innate lymphoid cell; MS, multiple sclerosis; MyD88, myeloid differentiation primary response 88; NLRP3, NOD‐like receptor pyrin domain‐containing protein 3; NOD, nucleotide‐binding oligomerization domain; PGN, peptidoglycan; PXR, pregnane X receptor; RIPK2, receptor‐interacting serine/threonine‐protein kinase 2; SCFAs, short‐chain fatty acids; LPS, lipopolysaccharide; sIgA, secretory immunoglobulin A; T1D, type 1 diabetes; TGR5, Takeda G protein‐coupled receptor 5; Th, T helper cell; TLR, Toll‐like receptor; TRAF6, tumor necrosis factor receptor‐associated factor 6; Treg, regulatory T cell; TRIF, TIR‐domain‐containing adapter‐inducing interferon‐β; VDR, vitamin D receptor.

The gut microbiota constitutes an integral component of host physiology and engages in continuous, bidirectional crosstalk with the immune system [242]. Rather than representing a passive microbial assemblage, this ecosystem functions as an important modulator of the development, maturation, and effector functions of host immunity (Figure 5) [243]. This symbiotic interaction contributes to the establishment and maintenance of mucosal and systemic immune homeostasis. Microbiota‐derived signals help educate the immune system to discriminate between innocuous commensal antigens and invasive pathogens, thereby promoting tolerance while preserving defensive capacity [244, 245]. Such programming encompasses multiple immune compartments, including T cell lineage differentiation, antibody production, and innate immune activation [244, 246]. Disruption of this finely tuned equilibrium can contribute to immune dysregulation across a broad spectrum of pathologies, ranging from IBD to systemic autoimmune, metabolic, and allergic disorders [243, 246]. The microbiota and host immunity can therefore be conceptualized as an integrated metasystem in which perturbations in one compartment may reshape the other, with implications for host defense and disease susceptibility [247].

### Foundational programming of mucosal and systemic immunity

#### Microbial education of T cell lineages: Balancing pro‐inflammatory T helper 17 (Th17) and Treg responses

The intestinal mucosa serves as a principal inductive site for adaptive immune education, where persistent microbial exposure contributes to the differentiation and specialization of T lymphocytes [248]. Central to this process is the maintenance of equilibrium between pro‐inflammatory effector T cells, essential for pathogen clearance, and immunosuppressive Tregs, required to sustain tolerance toward commensals and dietary antigens. Microbiota‐derived cues promote the development of both Th17 cells, which produce IL‐17 and, in some contexts, IL‐22 involved in mucosal barrier defense, and Foxp3+ Tregs that constrain excessive inflammation [244].

This equilibrium is dynamically regulated. Dendritic cells within the intestinal lamina propria integrate microbial cues through pattern‐recognition receptor‐related signaling; adaptor molecules such as TNF receptor‐associated factor 6 (TRAF6) are important for maintaining the balance between Treg induction and aberrant Th2 responses, thereby preserving mucosal tolerance [249]. Microbial signals also regulate T cell trafficking. For example, modulation of homing receptors such as GPR15 directs Treg migration to the colon, reinforcing local immune restraint [250].

Gut microbiota–dependent signals have been reported in experimental neuroinflammatory models to induce Notch3‐expressing Treg subsets that migrate to the central nervous system and acquire pathogenic Th17‐like features, thereby contributing to neuroinflammation [251]. Moreover, co‐housing experiments indicate that shifts in microbial composition can alter systemic T cell polarization, such as transitions from Th1 to Th17 dominance, altering susceptibility to autoimmune disease [252]. Collectively, these observations underscore the integral role of the microbiota in calibrating the Th17/Treg axis; failure to maintain this balance, together with dysbiosis and barrier dysfunction, contributes to immune‐mediated disorders [253, 254].

#### Shaping humoral defenses: Microbiota‐driven IgA production and compartmentalization

The gut microbiota is a major driver of mucosal humoral immunity, particularly the induction of secretory immunoglobulin A (sIgA), a dominant antibody class at mucosal surfaces [248]. sIgA helps mediate immune exclusion by limiting microbial‐epithelial contact and supporting host–microbe mutualism [255].

IgA induction occurs through complementary T cell‐dependent and T cell‐independent pathways. In Peyer's patches, Tfh cells support germinal center reactions that generate high‐affinity, antigen‐specific IgA. Optimized antibody responses may be supported through enhanced Tfh cell function, which has recently been reported to be promoted by microbial metabolites such as butyrate in experimental settings [256]. Concurrently, T cell‐independent responses contribute to broader, polyreactive, or homeostatic IgA responses through microbial and innate signals, including TLR‐associated pathways [248, 257].

Luminal sIgA shapes microbial ecology by limiting epithelial access, restricting bacterial translocation, and influencing community composition [248, 257]. Systemically, certain commensal Gram‐negative bacteria can elicit protective IgG responses that cross‐react with pathogens such as Salmonella [258]. Maternal IgG and IgA, shaped in part by maternal microbiota, are transferred to offspring and dampen neonatal adaptive responses to newly colonizing microbes, facilitating early‐life tolerance [259]. Notably, IgA‐secreting plasma cells with mucosal imprinting can populate distal sites, including the meninges [260], suggesting the systemic reach of gut‐associated humoral immunity [261].

#### Innate lymphoid cells (ILCs) as first responders at the host‐microbe interface

ILCs are tissue‐resident lymphocytes lacking antigen‐specific receptors but strategically positioned at mucosal barriers to rapidly respond to tissue‐ and microbe‐derived cues [262, 263]. ILC3s are abundant in the intestine and translate microbial and tissue‐derived signals into barrier‐protective outputs.

Upon stimulation by dendritic cell‐derived IL‐23 and related cytokines, ILC3s secrete IL‐22, which acts on IECs to promote Fut2‐dependent epithelial fucosylation, thereby supporting host–commensal resilience during infection or inflammatory stress [42]. Beyond the gut, microbiota‐derived molecules, including butyrate and AhR ligands, have been shown in NOD mouse models to promote IL‐22 secretion by pancreatic ILCs, inducing mouse β‐defensin 14 (mBD14) expression in pancreatic endocrine cells and preventing autoimmune diabetes in NOD mice [263].

Recent experimental evidence indicates that ILC3s can directly sense gut microbiota through stimulator of interferon genes (STING) signaling. At steady state, rather than primarily initiating inflammation, STING activation promotes CCR7‐dependent ILC3 migration to gut‐draining lymph nodes, where they function as unconventional antigen‐presenting cells to induce microbiota‐specific Tregs and establish adaptive tolerance [264]. Thus, ILCs help integrate innate barrier defense with tolerance programming at the host–microbe interface.

### Pattern recognition pathways: Sensing microbes to maintain homeostasis

#### TLRs: Gatekeepers of the gut–immune interface

Toll‐like receptor 4 (TLR4) functions as a key sensor of endotoxin and can link microbial products to metabolic inflammation by recognizing LPS, a major component of Gram‐negative bacteria [265]. In the intestine, epithelial TLR4 signaling is tightly controlled to permit tolerance to commensal‐derived LPS while supporting barrier repair [266]. Dysregulation of this pathway, however, drives systemic inflammation. Diets rich in saturated fats can promote dysbiosis and barrier dysfunction, increasing translocation of LPS into circulation, a process termed metabolic endotoxemia [265]. Chronic low‐grade LPS exposure can activate TLR4 on macrophages in adipose tissue and liver, as well as on endothelial cells [265, 267], inducing cytokine production that disrupts insulin signaling and contributes to insulin resistance and obesity [265, 268].

Aberrant TLR4 signaling also contributes to necrotizing enterocolitis in premature infants [269], gut‐derived endotoxin‐associated hypercoagulability in cirrhosis [267], and TLR4‐dependent aggravation of pancreatitis in dysbiotic states [270]. Thus, TLR4 can translate microbial imbalance into systemic inflammatory signaling and disease‐related phenotypes.

As a flagellin‐sensing receptor involved in shaping microbial composition, TLR5 detects flagellin from motile bacteria [271]. During neonatal life, high epithelial TLR5 expression shapes microbial colonization by inducing antimicrobial proteins such as REG3γ, thereby selecting for specific bacterial communities [272, 273].

In specific mouse models, TLR5‐deficient mice develop dysbiosis‐associated metabolic syndrome, including hyperphagia, adiposity, and insulin resistance [274]. This phenotype is transferable to germ‐free mice through fecal transplantation, supporting a causal contribution of the microbiota in this model [274]. TLR5 signaling also supports barrier integrity and has been linked in experimental studies to hepatic apolipoprotein A1 production, HDL levels, and feeding behavior via neuropod cell signaling in the gut–brain axis [273, 275, 276].

TLR2 contributes to the calibration of host–microbe interactions by recognizing bacterial lipoproteins/lipopeptides and related microbial pathogen‐associated molecular patterns (PAMPs) [277]. Beyond its canonical role in pathogen sensing, TLR2 can mediate protective host–microbe interactions, including epithelial barrier reinforcement and, in selected tumor models, enhancement of anti‐tumor immunity through recognition of commensal species such as *Akkermansia muciniphila* and *Alistipes finegoldii* [278, 279]. In addition, TLR2 signaling contributes to microbiota‐dependent enteric neurogenesis, further highlighting its role in coordinating mucosal and neural homeostasis [280].

In parallel, TLR9 participates in host–microbe immune interactions by detecting unmethylated CpG motifs in microbial DNA [281]. Homeostatic TLR9 signaling helps restrain immune overactivation; in experimental models, its deficiency can promote dysbiosis, inflammatory responses, and obesity‐related metabolic dysfunction [282]. TLR9 signaling also helps sustain E4BP4 expression in microbiota‐ and IL‐15‐dependent innate‐like B cell progenitors [283]. Together, TLR2 and TLR9 provide complementary microbial sensing that sustains immune equilibrium. Emerging evidence suggests that TLR2 and TLR9 may also intersect with immunometabolic regulation in mucosal and immune‐cell contexts. By linking microbial detection with cellular metabolic programs, these receptors may contribute to local immune‐cell metabolic regulation and mucosal homeostasis. This integration of microbial sensing with host metabolic pathways may help maintain a balanced immune environment at mucosal surfaces.

#### Cytosolic sensors in immune regulation

Among cytosolic sensors, NOD‐like receptors (NLRs) link microbial recognition to inflammatory signaling and autophagy. These receptors detect cytosolic microbial products and danger signals [284]. NOD1 and NOD2 recognize peptidoglycan fragments and activate NF‐κB and MAPK pathways to induce cytokine and antimicrobial responses [285].

NLR signaling, particularly pathways involving NOD2, is closely linked to autophagy. Risk variants in NOD2 and ATG16L1 predispose to Crohn's disease, and NOD2‐, ATG16L1‐, and IRGM‐related pathways cooperate in xenophagy of intracellular bacteria [286]. Defects can impair microbial clearance, promote dysbiosis, and sustain persistent inflammation [287]. Inflammasome‐forming NLRs such as NLRP3 and NLRP6 activate caspase‐1 to mature IL‐1β and IL‐18 [288]. These pathways integrate microbial sensing with autophagy and pyroptosis to maintain intestinal homeostasis [288].

The cGAS‐STING pathway represents another cytosolic sensing mechanism, linking microbial DNA detection to systemic immune priming and mucosal tolerance. It senses cytosolic DNA and induces type I interferon (IFN‐I) responses [289]. Gut bacteria can release membrane vesicles containing DNA that may enter the circulation, where they activate cGAS to produce cGAMP, subsequently triggering STING‐dependent IFN‐I production [289]. This process establishes a baseline “tonic” antiviral state, which is diminished when antibiotics reduce the gut microbiota [289].

Interestingly, STING activation in intestinal ILC3s drives their migration to lymph nodes and promotes the generation of microbiota‐specific Tregs, thereby supporting mucosal immune tolerance [264]. Together, these findings indicate that the cGAS–STING pathway plays context‐dependent roles, balancing antiviral defense with the maintenance of immune tolerance.

#### MyD88 and TRAF6: Integrators of microbial signals

MyD88 serves as the canonical adaptor for most TLRs, except TLR3, and for IL‐1 receptor family members [290, 291]. While frequently associated with pro‐inflammatory signaling, MyD88‐dependent pathways are also essential for microbiota‐driven immune homeostasis. Notably, in non‐obese diabetic mice, MyD88 deficiency can protect against autoimmune diabetes in a microbiota‐dependent manner, underscoring its role in host‐microbe crosstalk beyond inflammation [290].

TRAF6, an E3 ubiquitin ligase acting downstream of multiple innate and cytokine receptors, can exhibit context‐dependent tolerogenic roles [285]. Dendritic cell‐specific deletion of TRAF6 leads to reduced intestinal regulatory T cells and the development of Th2‐skewed enteritis, a phenotype divergent from that observed with MyD88 deficiency [249]. In IECs, TRAF6 contributes to barrier maintenance via pathways that are independent of canonical Toll‐like receptor signaling mediated by the adaptor proteins MyD88 and TIR‐domain‐containing adapter‐inducing interferon‐β (TRIF) [292]. Collectively, these adaptor molecules function as context‐dependent integrators of microbial cues rather than simple pro‐inflammatory switches.

### The chemical language of the microbiome: Metabolites as immune regulators

#### SCFAs

SCFAs, including acetate, propionate, and butyrate, are microbial metabolites produced through fermentation of dietary fiber by gut bacteria [293]. SCFAs exert multiple local and systemic effects, supporting gut barrier integrity, modulating immune responses, and contributing to metabolic homeostasis [230, 294]. They can influence immune function by promoting regulatory T cell differentiation, enhancing anti‐inflammatory cytokine production, and suppressing excessive inflammatory responses in the gut and periphery [293, 294]. SCFAs can also impact cardiovascular function, including context‐dependent effects on blood pressure through receptors such as GPR41 and GPR43 [230]. Collectively, SCFAs serve as key mediators linking gut microbial activity to host immunity, metabolic regulation, and overall systemic homeostasis.

Butyrate also inhibits HDACs, promoting Treg differentiation and macrophage hyporesponsiveness [295, 296]. SCFAs reinforce epithelial integrity and modulate systemic T and B cell phenotypes [295, 297]. Reduced SCFA production or reduced abundance of SCFA‐producing taxa has been linked to inflammatory and autoimmune disorders [296, 298].

#### Tryptophan catabolites and AhR signaling

Microbial tryptophan metabolism generates several indole derivatives that can activate AhR [299]. AhR activation promotes IL‐22 production, reinforces epithelial barrier integrity, and supports regulatory immune responses [299]. Impaired production of AhR ligands has been observed in inflammatory diseases such as celiac disease, whereas AhR activation ameliorates experimental pathology [299]. For example, IPA has been reported to reduce IL‐17A expression in Th17 cells by suppressing mTOR activity and modulating ribosome‐related pathways [300], while indole‐3‐lactic acid has been implicated in the regulation of AhR/NRF2/NLRP3 inflammasome‐related pathways in experimental colitis [301]. In addition, microbiota‐derived indoles may extend AhR‐dependent effects beyond the intestine to the gut–brain axis. Experimental studies have shown that microbiota‐derived indole can promote adult hippocampal neurogenesis and enhance neuronal differentiation and synaptic maturation in vivo [207].

#### Secondary bile acids: Immune modulation via FXR and TGR5

Primary bile acids synthesized in the liver are transformed by gut microbiota into secondary bile acids such as DCA and LCA, which possess distinct signaling properties [302]. These metabolites regulate immunity and metabolism through bile acid‐sensitive receptors, including FXR, TGR5/GPBAR1, VDR, and PXR, with receptor engagement depending on bile acid species, concentration, and tissue context [302]. These receptors are expressed along the gut–liver axis and on immune cells including macrophages and dendritic cells.

Secondary bile acids influence macrophage polarization, T cell differentiation, and inflammatory tone [302]. Their effects are context‐dependent: high DCA levels associated with high‐fat diets can promote pro‐inflammatory macrophage programs and exacerbate colitis in experimental settings [303], whereas LCA or LCA‐derived metabolites have been reported to restrain pathogenic Th17 expansion and mitigate intestinal or hepatic injury through bile acid receptor‐dependent mechanisms [304]. Altered bile acid pools are associated with IBD, liver disease, and carcinogenesis, and depletion or modulation of secondary bile acid‐producing bacteria can alter liver tumor development in dysbiotic models [305].

#### Polyamines: Epithelial and immune homeostasis

Gut microbes and host cells metabolize amino acids such as arginine and ornithine to produce polyamines, including putrescine, spermidine, and spermine. These metabolites can enhance epithelial barrier integrity by promoting cell proliferation, strengthening tight junctions, and facilitating mucosal repair [306]. Polyamines also regulate autophagy and cellular stress responses. In the immune system, they influence T cell differentiation, suppress excessive inflammatory signaling, and modulate macrophage polarization. Additionally, microbial cadaverine, a polyamine produced by bacterial decarboxylation of lysine, influences local immune function in the gut. In a recent study, cadaverine was shown to reprogram macrophage immunometabolism in a context‑ and concentration‑dependent manner, such that at baseline levels it promotes oxidative phosphorylation and an immunoregulatory phenotype, while at higher concentrations it can drive glycolysis and pro‑inflammatory functions through engagement of the histamine 4 receptor [307]. Cadaverine thus acts as a microbiota‐derived metabolite that can directly modulate innate immune cell behavior and shape intestinal inflammatory responses.

#### Phenolic metabolites: Microbial modulators of immunity and metabolism

Microbial metabolism of dietary polyphenols and aromatic amino acids generates phenolic and aromatic co‐metabolites, including 4‐cresol, phenylacetylglutamine, catechol derivatives, and hydroxyphenylacetic acid. These molecules can modulate host physiology by influencing immune signaling, modulating hepatic and cardiovascular metabolism, and participating in antioxidant and redox pathways [308]. Phenolic and aromatic co‐metabolites may act as chemical intermediates linking microbial activity with host immune responses, oxidative stress control, and systemic metabolic homeostasis. For instance, 4‐cresol has been linked to pro‐inflammatory responses and ROS‐related oxidative stress [309], while phenylacetylglutamine has been shown to enhance platelet reactivity and contribute to thrombotic potential via adrenergic receptors, linking elevated phenylacetylglutamine to CVD risk [196].

Phenolic metabolites also contribute to metabolic homeostasis, including modulation of lipid metabolism and energy expenditure, and some, such as urolithins derived from ellagic acid, may improve mitochondrial function and reduce oxidative or inflammatory stress in host tissues [310, 311, 312]. Additionally, some of these molecules can act as antioxidants or redox modulators, scavenging reactive oxygen species or activating host antioxidant pathways, thereby influencing tissue repair and inflammation.

#### Microbial vitamins: Linking metabolism and immune function

Certain gut microbes synthesize essential vitamins, notably B‑group vitamins such as riboflavin (B2), niacin (B3), pyridoxine (B6), and cobalamin (B12), as well as menaquinone (vitamin K2), contributing to the intestinal vitamin pool, although the extent to which colonic microbial production supplements systemic host requirements varies by vitamin and absorption site [313, 314]. These vitamins serve as important cofactors for host enzymatic reactions, redox balance, nucleotide synthesis, and amino acid metabolism, underlining their importance in fundamental physiological processes [313]. Beyond these metabolic roles, microbial vitamin derivatives also influence immune function. Intermediates from the microbial riboflavin biosynthesis pathway are presented by the non‐polymorphic MHC class I‐related protein MR1 and activate mucosal‐associated invariant T (MAIT) cells, linking bacterial vitamin metabolism to mucosal immune surveillance [314, 315]. Additionally, niacin (vitamin B3) acts as a ligand for GPR109A/HCAR2 expressed on epithelial and immune cells; engagement of this receptor promotes regulatory T cell differentiation and exerts anti‑inflammatory effects in colitis models [316]. Collectively, microbial vitamin production integrates metabolic support with immune modulation, providing a direct biochemical link between the gut microbiota and host homeostasis.

#### Microbial extracellular vesicles (EVs): Long‐distance immunomodulation

Gut bacteria release EVs, including outer membrane vesicles from Gram‐negative species, which package proteins, lipids, and nucleic acids [289, 317, 318]. These vesicles can traverse the mucus layer and interact with epithelial and immune cells, delivering concentrated immunomodulatory signals. EV cargo can engage receptors such as TLR2 and TLR4 to induce tolerogenic dendritic cell phenotypes or stimulate epithelial and mucosal immune responses, including IgA‐related pathways, reinforcing mucosal defense [317, 319].

In experimental settings, bacterial EVs can enter systemic circulation. Microbial DNA carried within EVs activates the cGAS‐STING pathway in peripheral tissues, establishing tonic type I interferon responses that enhance antiviral readiness [289]. Functional outcomes depend on bacterial origin. EVs from probiotic species such as *Lactobacillus johnsonii* have been reported to restore Th17/Treg balance and ameliorate colitis in experimental models [253], whereas EVs from disease‐associated *Escherichia fergusonii* may contribute to neuroinflammation via vagus nerve‐related gut–brain signaling in mice [318]. EVs, therefore, represent a potentially important mechanism of both local and systemic microbiota–host communication.

### Immunometabolic reprogramming and microbiome‐driven microenvironmental heterogeneity

Beyond canonical immune signaling, an important mechanism linking the microbiome to host disease may lie in the immunometabolic reprogramming of immune cells within local tissue microenvironments. Microbial metabolites act not only as signaling molecules but also as metabolic substrates and regulators that reshape intracellular metabolic pathways in immune cells, thereby influencing their functional states [320]. For example, SCFAs can promote oxidative metabolism and regulatory phenotypes in immune cells, whereas inflammatory conditions associated with dysbiosis favor glycolytic reprogramming and pro‐inflammatory activation, particularly in macrophages [320, 321]. Similarly, tryptophan metabolites and bile acid derivatives can modulate immune‐metabolic signaling, metabolic checkpoints, and macrophage polarization, thereby coupling microbial metabolism to immune cell fate decisions and inflammatory outputs [320, 321]. These processes support a mechanistic link between microbial ecology and the metabolic wiring of the immune system, which is critical for both the initiation and persistence of chronic inflammation.

In the context of complex diseases such as cancer, microbial communities are increasingly recognized as components of some tumor microenvironments, where microbial communities may exhibit marked spatial heterogeneity and functional specialization [322, 323]. Intratumoral and peritumoral microbial communities can differ substantially in composition and metabolic activity, reflecting localized ecological constraints such as hypoxia, nutrient gradients, and immune pressure [323]. These spatially structured microbial populations interact with tumor‐infiltrating immune cells and stromal components, shaping local immunometabolic landscapes. For instance, microbial metabolites have been implicated in macrophage polarization, T cell dysfunction or activation, and antigen presentation. Conversely, tumor‐associated metabolic states, including hypoxia and altered lipid and amino acid metabolism, may help shape microbial niches and select for taxa adapted to these niches, reinforcing a bidirectional interaction between microbial ecology and tumor biology.

Importantly, this spatial and metabolic heterogeneity can be viewed as a manifestation of microbiome‐associated functions extending into diseased tissues. Disruption of normal compartmentalization allows microbes or their metabolites to access sites beyond the intestinal lumen, contributing to systemic and local pathophysiology. In inflammatory diseases, localized microbial encroachment and metabolite gradients can sustain immune activation through continuous metabolic reprogramming of resident immune cells. In tumors, microbiota‐associated metabolic circuits can either promote immune evasion or enhance immune‐mediated tumor control, depending on context [320, 321, 322, 323].

These insights have significant translational implications. Targeting microbiome‐driven immunometabolic pathways offers a strategy to modulate disease‐specific microenvironments. Approaches may include selective manipulation of microbial metabolites, spatially targeted microbiota modulation, or combination therapies that integrate microbiome interventions with immunotherapy or metabolic modulators. A deeper understanding of the spatial organization of microbiota within tissues, together with the metabolic states of host cells, will be essential for developing precision interventions that reshape local immune environments and improve clinical outcomes.

### When homeostasis fails: Microbiome‐immune dysregulation

#### IBD: Breakdown of mucosal tolerance

IBD, including Crohn's disease and ulcerative colitis, is associated with disruption of host–microbiota interactions [324]. In genetically susceptible individuals, impaired tolerance toward commensal microbes can contribute to chronic inflammation [287]. Dysbiosis, often characterized by reduced diversity, depletion of SCFA‐producing taxa such as *Faecalibacterium*, and expansion of pathobiont‐associated taxa including adherent‐invasive *Escherichia coli* and *Fusobacterium* species, is a recurrent feature [325].

Genetic risk loci frequently involve autophagy genes (ATG16L1, IRGM) and epithelial defense pathways essential for microbial clearance and barrier maintenance [287]. Barrier dysfunction may increase microbial exposure and contribute to activation of innate immune pathways; in this inflammatory context, dendritic‐cell activation and Th1 and Th17 responses are also implicated in IBD [326]. Inflammation can further disrupt microbial ecology, potentially establishing a self‐perpetuating cycle characteristic of IBD [287].

#### Autoimmunity and allergy: Microbiota‐driven immune dysregulation

Type 1 diabetes (T1D) is a T cell‐mediated autoimmune disease that can be influenced by microbial factors in genetically predisposed hosts [290]. Molecular mimicry represents one mechanism: commensal peptides, including those from *Fusobacteria*, can resemble islet autoantigens such as IGRP, potentially activating autoreactive CD8 + T cells that contribute to pancreatic β‐cell destruction [327].

In parallel, disruption of microbiota‑dependent immune tolerance can exacerbate disease; for example, reduced production of SCFAs may diminish protective pathways such as the expression of the cathelicidin‑related antimicrobial peptide (CRAMP) by β cells, which supports regulatory immune circuits. Loss of these regulatory mechanisms permits unchecked autoreactive T cell activity and accelerates progression [297].

In neuroinflammatory autoimmunity, experimental autoimmune encephalomyelitis (EAE), a model of multiple sclerosis, is attenuated under germ‐free conditions or after antibiotic treatment, implicating the microbiota in its pathogenesis [328]. Microbial products such as bacterial peptidoglycan may further amplify neuroinflammation in experimental contexts by activating innate immune pathways and promoting gut–brain inflammatory signaling [329].

Conversely, SCFAs limit central nervous system (CNS)‐directed T cell trafficking through GPR43 signaling, exerting protective effects [294]. The microbiota thus bidirectionally modulates neuroinflammatory disease.

In asthma and atopy, early‐life microbial exposure critically shapes immune tolerance [245]. Infants with altered gut microbial colonization profiles, often characterized by reduced richness and diversity, have been associated with increased risk of allergic sensitization, eczema, and asthma in later childhood. Birth cohort studies such as the Canadian Healthy Infant Longitudinal Development (CHILD) study have shown that infants who later develop wheeze and atopy exhibit lower abundances of key commensal genera early in life, suggesting that early microbial disparities may contribute to maladaptive immunity and atopic disease trajectories [330]. Disruptions such as Cesarean delivery, antibiotics, or reduced environmental microbial diversity can favor Th2‐skewed responses and allergic disease susceptibility [245, 331]. Dysbiosis in individuals with asthma, marked by depletion of immunomodulatory taxa such as *Bifidobacterium* and *Faecalibacterium*, correlates with impaired production of regulatory metabolites like SCFAs and altered tryptophan metabolites, skewing the balance toward type 2 (Th2)‑dominant inflammation and airway hyperresponsiveness [332]. Vertical transmission of maternal microbes influences neonatal immune development; specific *Lactobacillus* strains induce immunosuppressive phenotypes and protect against airway inflammation in models [333]. Environmental microbial richness, including soil exposure, can promote tolerogenic programming [331].

Host genetic factors, including TLR4 and CD14 variants, further modulate these interactions [334]. Failure to establish balanced host–microbe communication during this developmental window may predispose to long‐term allergic disease.

#### Metabolic inflammation

Metabolic syndrome is often accompanied by chronic low‐grade inflammation (metaflammation) [335]. Western dietary patterns can induce dysbiosis and compromise epithelial barrier integrity [265]. Increased permeability can permit LPS translocation, contributing to metabolic endotoxemia [336]. LPS‐driven TLR4 activation on macrophages sustains inflammatory cytokine production that disrupts insulin signaling and promotes insulin resistance [265, 336].

Experimental genetic models support a causal contribution of the microbiota in selected settings. TLR5‐deficient mice develop transmissible dysbiosis‐associated metabolic syndrome; transfer of gut microbiota from TLR5‐deficient mice to wild‐type germ‐free recipients confers many features of metabolic syndrome [274]. In this model, dysbiosis also promotes hepatic lipogenesis, further reinforcing metabolic pathology [337].

#### Anti‐tumor immunity and cancer immunotherapy

The gut microbiota is an important determinant or modifier of response to immune checkpoint inhibitors (ICIs) and susceptibility to ICI‐associated colitis [338, 339]. Distinct microbial signatures have been correlated with therapeutic efficacy, although signatures vary across cohorts and cancer types [340]. Certain commensals enhance dendritic‐cell chemokine production and CD8+ T cell recruitment; for example, *Alistipes finegoldii* activates TLR2 signaling to promote CXCL16 production within the tumor microenvironment [279].

Microbial metabolites also reshape immune tone. Products from *Limosilactobacillus reuteri* have been reported to induce macrophage reprogramming toward tumoricidal phenotypes in preclinical models, enhancing anti‐tumor immune responses [341]. Strategies including fecal microbiota transplantation, defined bacterial supplementation, and postbiotic metabolite administration are under investigation to overcome ICI resistance [340]. Some metabolites, such as indole‐3‐carboxaldehyde, may mitigate ICI‐induced colitis without compromising anti‐tumor efficacy in preclinical models [342].

### Section summary

The microbiome functions as an active regulator of host immunity within an integrated microbiome organ‐like framework. Through coordinated pattern‐recognition signaling and metabolite‐mediated communication, the microbiota calibrates immune development, maintains mucosal tolerance, and shapes systemic inflammatory tone. Dysbiosis can disrupt this dialog and may represent a shared mechanistic contributor across inflammatory, autoimmune, metabolic, allergic, and neoplastic diseases.

Advances in multi‐omics profiling offer opportunities to define microbiome‐derived biomarkers for disease prediction, patient stratification, and therapeutic response forecasting, particularly in respiratory diseases [245]. Therapeutic approaches are evolving toward mechanistically informed interventions, including probiotics, engineered bacteria, fecal microbiota transplantation, metabolite supplementation, bile acid pathway modulation, and selective antibiotic or phage‐based strategies [343].

Despite substantial progress, the relationship between the microbiome and disease remains complex and, in many cases, incompletely causal. Many reported associations are influenced by confounding factors such as diet, medication use, and host genetics, which can limit reproducibility across cohorts. Moreover, microbiome‐targeted interventions have yielded variable and sometimes inconsistent clinical outcomes, reflecting underlying heterogeneity in microbial composition and host responses. In some settings, modulation of the microbiome alone has proven insufficient to reverse established disease states, underscoring the multifactorial nature of these conditions. These challenges highlight the need for cautious interpretation of microbiome–disease relationships and reinforce the importance of mechanistic validation, longitudinal study design, and stratified therapeutic approaches.

Addressing these challenges will require integrating microbial‐ecosystem perspectives with immunology and clinical stratification to account for inter‐individual variability and dynamic host–microbe interactions [344]. Precision restoration of microbiome–immune homeostasis represents an important objective for next‐generation translational medicine.

## THE MICROBIOME–GUT–BRAIN AXIS WITHIN AN ORGAN‐LIKE FRAMEWORK

### Overview

**FIGURE 6:**
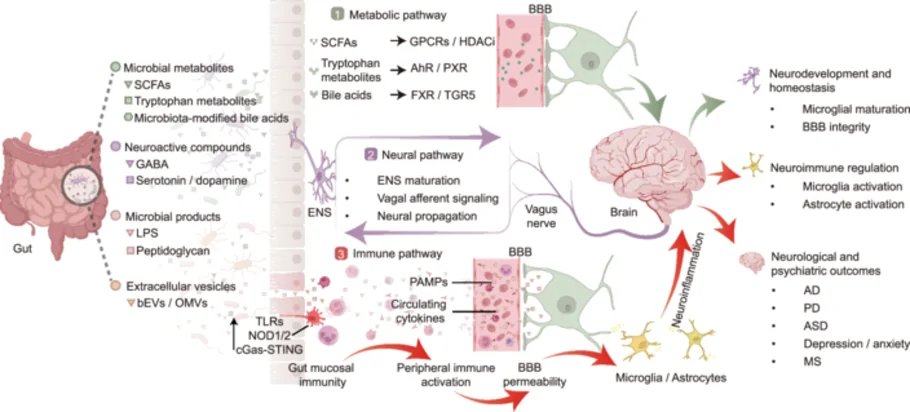
The microbiome–brain axis. Schematic overview of bidirectional communication between the gut microbiome and the brain through metabolic, neural, and immune pathways. Microbial metabolites, neuroactive compound precursors or modulators, immune mediators, microbial products, and extracellular vesicles may influence blood–brain barrier integrity, enteric and vagal signaling, neuroimmune cell responses, and brain homeostasis. These pathways have been associated with, and experimentally implicated in, neurodevelopmental, neuroimmune, neuropsychiatric, and neurodegenerative disease‐related phenotypes, particularly under conditions of dysbiosis, barrier disruption, or systemic inflammation. AhR, aryl hydrocarbon receptor; BBB, blood–brain barrier; bEVs, bacterial extracellular vesicles; CNS, central nervous system; ENS, enteric nervous system; FXR, farnesoid X receptor; GABA, gamma‐aminobutyric acid; GPCRs, G protein‐coupled receptors; HDACi, histone deacetylase inhibition; LPS, lipopolysaccharide; OMVs, outer membrane vesicles; PAMPs, pathogen‐associated molecular patterns; PXR, pregnane X receptor; SCFAs, short‐chain fatty acids; cGAS–STING, cyclic GMP‐AMP synthase–stimulator of interferon genes; TGR5, Takeda G protein‐coupled receptor 5; TLRs, Toll‐like receptors; VDR, vitamin D receptor.

The bidirectional communication network linking the gastrointestinal tract and the CNS, known as the gut–brain axis, contributes to host homeostasis [345]. In recent years, gut microbiota has emerged as an important regulator of this axis, leading to the establishment of the microbiota–gut–brain axis concept (Figure 6) [345, 346].

Accumulating evidence indicates that gut microbiota influences multiple host physiological processes, including neuroimmune development, brain homeostasis, and complex behavioral and cognitive processes such as anxiety‐like behavior, learning, and memory [347]. This dialog between microbes and the brain is mediated through diverse pathways, including the immune system, the vagus nerve, the enteric nervous system (ENS), and the production of neuroactive microbial metabolites such as SCFAs and tryptophan derivatives [345, 348].

Perturbations in the composition and function of the gut microbiota, collectively referred to as dysbiosis, are increasingly associated with, and may contribute to, the pathophysiology of a wide spectrum of CNS disorders [345]. These conditions span neurodevelopmental disorders such as autism spectrum disorder (ASD), neuropsychiatric disorders including depression, anxiety, and schizophrenia, and neurodegenerative diseases such as Alzheimer's disease (AD) and Parkinson's disease (PD) [345, 349]. Studies in germ‐free animals have revealed substantial effects of gut microbiota on neural processes [345, 350]. Recognition of this axis has opened new therapeutic avenues, suggesting that modulation of the gut microbiota could represent a potential adjunctive or preventive strategy for selected complex CNS disorders. Accordingly, interventions such as probiotics, prebiotics, and other psychobiotics, broadly defined as microbiome‐targeted interventions, including probiotics and in some definitions prebiotics or related substrates, that may confer mental health or brain‐related benefits through the microbiota–gut–brain axis, are being actively investigated as potential therapies for brain disorders [351, 352].

### The microbiome organ–brain axis: Mechanisms of bidirectional communication

#### Neural pathways: ENS and vagal routes of gut–brain communication

The ENS is a complex network of neurons and glia within the gut wall that provides intrinsic neural control of digestion and contributes to mucosal defense and barrier function [353]. Located in close proximity to the luminal environment, the ENS continuously interacts with the gut microbiota, which contributes to its development, maturation, and maintenance from early life into adulthood [280, 353]. This interaction is facilitated by specialized gut chemosensors, including serotonergic enterochromaffin (EC) cells that detect dietary, microbial, and inflammatory stimuli. EC cells are electrically excitable and form synaptic‐like contacts with primary afferent nerve fibers, enabling direct transduction of gut‐derived signals to the nervous system [354]. Experimental work has shown that microbiota‐induced enteric serotonin promotes maturation of the adult ENS through activation of the 5‐HT4 receptor, modifying ENS neuroanatomy and increasing intestinal transit rates [355].

Microbial influence may also extend to adult ENS maintenance and enteric neurogenesis. In the adult mouse colon, intestinal bacteria help maintain nitrergic neurons through Toll‐like receptor 2 (TLR2)‐dependent neurogenesis from Nestin‐positive enteric neural precursor cells [280]. Functionally, microbial–ENS interactions can shape metabolic physiology by regulating GLP‐1‐dependent gut–brain signaling. In type 2 diabetic mice, specific ileal dysbiosis has been reported to impair the GLP‐1‐activated gut–brain axis through enteric nitric oxide‐dependent mechanisms, thereby reducing GLP‐1 responsiveness in the control of insulin secretion and gastric emptying [356]. Microbial regulation of ENS development may begin before birth. In mice, antibiotic‐induced maternal gut dysbiosis before conception has been shown to disrupt embryonic ENS development in offspring and increase their susceptibility to water avoidance stress in adulthood, highlighting the preconception–gestational period as a sensitive developmental window [357]. Collectively, these findings position the ENS as an important interface through which microbial signals are translated into neural outputs regulating gut and systemic physiology.

The vagus nerve serves as one major anatomical and functional conduit through which gut microbial signals are relayed to the brain and influence behavior [345, 358]. This cranial nerve conveys gastrointestinal visceral and chemical signals, including nutrient‐related inputs, and is particularly important for transmitting gut l‐glutamate information to the brain [359]. Gut–brain communication can involve complex reflex circuits; for example, microbiota‐mediated modulation of gut‐extrinsic sympathetic neurons occurs via vagal afferents projecting from the distal intestine to the brainstem [360]. Similarly, the liver‐brain‐gut neural arc allows hepatic vagal sensory afferents to sense the gut microenvironment and to regulate intestinal immune homeostasis through parasympathetic efferent nerves [361].

Beyond physiological regulation, the vagus nerve may facilitate pathological propagation between the gut and brain. In Parkinson's disease, the gut‐first hypothesis proposes that misfolded α‐synuclein pathology may begin in the ENS and spread centrally via vagal pathways [362]. Experimental models further suggest that gut inflammation can promote vagal transmission of amyloid‐β (Aβ) and Tau fibril‐related pathology, while vagotomy attenuates this process [363]. Experimental evidence also supports possible reverse transmission, with tau pathology propagating from the brain to the colon via vagal efferent fibers [364]. Microbial metabolites contribute to vagal signaling; kynurenic acid can inhibit vagal afferents to regulate appetite [365], and altered tryptophan metabolism has been implicated in vagus nerve‐related inflammatory regulation [366]. These observations underscore the vagus nerve as a critical communication axis integrating physiological signaling and disease propagation.

#### Microbial metabolites as neuroactive messengers: SCFAs and tryptophan metabolism

SCFAs are produced through bacterial fermentation of dietary fiber and represent one major class of mediators through which the gut microbiota communicates with the brain [367, 368]. SCFAs can exert systemic effects by entering circulation and influencing CNS function directly or indirectly; some SCFAs or their downstream effects may involve transport across the blood–brain barrier (BBB) or modulation of neural, immune, and endocrine pathways [369]. Reduced abundance of SCFA‐producing bacteria and altered fecal SCFA levels have frequently been reported in Parkinson's disease, with experimental studies suggesting that SCFAs can modulate α‐synuclein‐related pathology and neuroinflammation [368]. Long‐term dietary fiber deficiency similarly reduces SCFA production and is linked to cognitive impairment and microglia‐mediated synaptic loss in mice, effects reversible with SCFA supplementation [370].

SCFAs may exert neuroprotective effects through immunomodulation, epigenetic regulation, barrier support, and neural or endocrine signaling. Acetate has been reported to suppress neuroinflammation in the rostral ventrolateral medulla in experimental settings and modulates glial morphology, contributing to antihypertensive effects [371]. Vancomycin‐induced depletion of acetate‐producing bacteria promotes cognitive impairment in diabetic mice via vagus‐dependent mechanisms [372]. Butyrate and valerate have been reported to alleviate stress‐induced depressive‐like behaviors in animal models by reducing hippocampal neuroinflammation [373]. Mechanistically, SCFAs signal through G protein‐coupled receptors and act as histone deacetylase inhibitors, enabling epigenetic regulation of gene expression [368, 374, 375]. Butyrate‐induced expression of neural CRAMP reduces EAE severity by regulating microglial and astrocyte activation [375]. SCFA‐producing *Lachnospira eligens* supplementation restored intestinal barrier and BBB integrity in an epilepsy model, accompanied by increased serum butyrate levels [376]. Strategies that modulate SCFA profiles or microbiota‐derived gut–brain metabolic signaling, including dietary interventions and selected probiotic or live biotherapeutic approaches, may improve brain‐related outcomes in specific experimental or clinical contexts by attenuating neuroinflammation and supporting neuronal integrity or neurogenesis [352, 377, 378, 379].

Beyond reducing inflammation, SCFAs contribute to the development and homeostatic maturation of microglia. Germ‐free mice display altered microglial morphology, gene expression, and immune responsiveness characterized by an immature phenotype with altered cell proportions and defective innate responses. Recolonization with a complex microbiota or supplementation with a mixture of major SCFAs, including acetate, propionate, and butyrate, partially restored microglial morphology and maturation‐associated features in vivo, supporting a role for microbiota‐derived SCFAs in microglial maturation and homeostasis [380].

Additionally, SCFAs, especially acetate, can be converted to acetyl‐CoA in neural cells, supporting energy metabolism and neurotransmitter synthesis [368, 381]. Butyrate may also influence serotonin and dopamine‐related pathways indirectly through epigenetic modulation of related genes.

Tryptophan, an essential dietary amino acid, serves as a precursor of serotonin and kynurenine‐pathway metabolites, forming a key metabolic interface in microbiota–gut–brain communication [139, 345]. The gut microbiota contributes to tryptophan metabolism, converting tryptophan into indole derivatives. Some indole derivatives signal through AhR, whereas IPA also acts through PXR‐dependent, antioxidant, and other pathways, thereby linking microbial metabolism to neural, immune, and metabolic pathways [139, 382]. Disruptions in this metabolic balance may contribute to disease; microbiota‐driven diurnal rhythms in tryptophan metabolism are susceptible to stress and influence gut barrier function [382].

Microbial tryptophan metabolites can modulate neurophysiology through direct and indirect mechanisms. IPA can cross the BBB and has been reported to exert neuroprotective effects in Alzheimer's disease models, at least in part through neuronal PXR activation, thereby reducing Aβ accumulation and attenuating neuroinflammation [383]. IPA has also been reported to improve social deficits and neural abnormalities in autism spectrum disorder models through ERK1‐related signaling [384]. On the kynurenine branch, dysregulation of the kynurenine pathway can alter the balance between kynurenic acid, which is often considered neuroprotective or NMDA receptor‐antagonistic, and quinolinic acid, which can be neurotoxic at elevated concentrations [385]. An obesogenic diet elevates hippocampal kynurenine and quinolinic acid, contributing to glutamic acid‐triggered neuroexcitotoxicity and hippocampus‐dependent cognitive impairment; supplementation with Eucommiae cortex polysaccharides mitigates these effects by reshaping the gut microbiota and modulating tryptophan metabolism [386]. These findings support microbial tryptophan metabolism as an important contributor to neurophysiology and a potential therapeutic target.

#### Gut‐immune‐brain crosstalk: A tripartite system in health and disease

At the barrier interface, the gut microbiota helps regulate blood–brain barrier integrity. The BBB is a highly selective interface that protects the CNS from peripheral insults, and its integrity can be influenced by the gut microbiota [387]. Early‐life microbiome establishment is particularly important for BBB development. Colonization of germ‐free mice with a mature microbiota derived from full‐term infants decreases BBB permeability compared with colonization by a prematurity‐associated microbiome, accompanied by transcriptional regulation of synaptic signaling genes within BBB cellular components [387]. In adulthood, SCFA‐producing gut bacteria may support BBB integrity, as *Lachnospira eligens* supplementation increased serum butyrate and restored cortical occludin expression in an adult epilepsy model [376, 388].

Gut dysbiosis can compromise BBB integrity through mechanisms often initiated by intestinal barrier disruption. Increased gut permeability permits microbial components, including LPS, to enter systemic circulation and induce peripheral inflammation [388]. Elevated circulating endotoxin may then promote neuroinflammation by disrupting BBB integrity, activating BBB‐associated inflammatory pathways, and facilitating cytokine release or immune‐cell recruitment into the brain [389]. Bacteria‐derived extracellular vesicles may provide an additional route of gut–brain communication by carrying inflammatory cargo, including LPS, to distant host tissues and the CNS, where they can modulate central immune responses [390]. This cascade linking intestinal barrier failure, systemic inflammation, and BBB disruption is implicated in neurological disorders; for example, Western‐style diet‐induced dysbiosis promotes BBB impairment and amyloid‐β pathology in Alzheimer's disease models [391]. Restoration of gut barrier function, including administration of *Akkermansia muciniphila*‐derived extracellular vesicles, can restore BBB integrity and alleviate cognitive deficits in experimental models, highlighting therapeutic potential [392].

At the level of CNS‐resident immune cells, the gut microbiota shapes microglial and astrocyte states. It contributes to CNS neuroimmune homeostasis by regulating microglial maturation, identity, and function through gut–brain axis signaling [393]. Microglia require microbiome‐derived signals for normal maturation and functional competence in experimental models. Germ‐free mouse studies demonstrate that the absence of microbiota leads to immature microglial phenotypes characterized by altered morphology, impaired immune responsiveness, and altered synaptic‐pruning programs [350, 393]. Single‐cell transcriptomic analyses further indicate that microbiome status can modulate the balance of microglial subpopulations, with shifts associated with susceptibility to Alzheimer's disease and major depressive disorder (MDD) [394].

Microglial modulation represents a key pathogenic mechanism across neurological disorders. In Parkinson's disease models, the gut microbiota has been shown to contribute to microglial activation, α‐synuclein pathology, and motor deficits [395]. In AD models, gut microbiota depletion or microbiome perturbation reduces pro‐inflammatory microglial phenotypes and attenuates amyloid plaque deposition [396]. Microbial metabolites also contribute to this crosstalk; gut microbiota‐derived histamine restores homeostatic spinal microglial states through intestinal H3R signaling, thereby promoting the resolution of peripheral inflammation [397]. Conversely, bacterial components such as LPS can prime microglia toward neurotoxic inflammatory responses [389]. Astrocytes are also influenced, with microbiome perturbation reducing reactive astrocytosis and shifting astrocytes toward homeostatic phenotypes through both microglia‐dependent and independent mechanisms [398].

Through peripheral immune signaling, gut‐derived PAMPs can propagate inflammatory signals to the CNS, particularly when intestinal barrier integrity is compromised. The gut microbiome can influence CNS function through peripheral immune signaling, particularly when PAMPs enter systemic circulation after intestinal barrier disruption [345, 399]. LPS, a major component of the outer membrane of Gram‐negative bacteria, is a representative bacterial PAMP/endotoxin [389]. Elevated circulating LPS can induce systemic inflammation that signals across or disrupts the BBB, leading to microglial activation and contributing to neurodegenerative processes, consistent with the endotoxin hypothesis of neurodegeneration [389].

Other PAMPs, including peptidoglycan (PGN), also participate in gut–immune–brain crosstalk. PGN fragments can enter circulation and be transported to the CNS by myeloid leukocytes, amplifying neuroinflammation and exacerbating autoimmune pathology in multiple sclerosis models [329]. Gut immune dysregulation can additionally alter peripheral immune cell trafficking. Chronic intestinal inflammation may promote expansion of commensal‐specific CD4^+^ T cells that infiltrate the CNS, where molecular mimicry‐driven reactivation leads to production of cytokines such as GM‐CSF, IFNγ, and IL‐17A, triggering microglial activation and neurological damage [400]. This pathway illustrates how gut‐derived immune responses may contribute to extraintestinal neuroinflammation.

#### Microbiome–gut–brain axis in neurodegenerative, neuropsychiatric, and neurodevelopmental disorders

Dysregulation of the microbiome–gut–brain axis has been reported in multiple neurodegenerative diseases. In Alzheimer's disease, gut dysbiosis has been associated with peripheral and central pathology and may contribute mechanistically in experimental models. Evidence from mouse models suggests that Th1 cell infiltration can drive M1 microglial activation and neuroinflammation, although the corresponding mechanisms in humans remain incompletely defined [401]. FMT studies in animal models provide causal evidence; microbiota from AD patients can exacerbate Aβ pathology, neurofibrillary tangles, and cognitive deficits in germ‐free AD mouse models [396]. Gut dysbiosis may also increase intestinal Aβ42 production, potentially expanding a peripheral Aβ source that could influence the brain via circulation‐dependent or immune‐mediated pathways [402].

In Parkinson's disease, frequently reported dysbiosis includes reduced abundance of SCFA‐producing bacteria and enrichment of pro‐inflammatory or mucin‐associated taxa [403]. Preclinical evidence suggests that the gut microbiota can contribute to α‐synuclein pathology, microglial activation, and motor impairment [395]. FMT from PD patients promotes increased motor dysfunction in α‐synuclein‐overexpressing mice compared with microbiota from healthy donors [395]. The gut‐first hypothesis proposes that misfolded α‐synuclein propagates from the ENS to the brain via the vagus nerve [362]. Microbiome influences extend to other neurodegenerative and neuroinflammatory disorders: *Akkermansia muciniphila* ameliorates amyotrophic lateral sclerosis progression via production of nicotinamide [404], and gut microbiota‐induced Notch3^+^ regulatory T cell subsets in multiple sclerosis can translocate to the CNS and paradoxically promote neuroinflammation [251].

The microbiome–gut–brain axis has also been implicated in neuropsychiatric and neurodevelopmental disorders. In depression‐related human cohorts, *Faecalibacterium* and *Coprococcus* are associated with higher quality‐of‐life indicators, whereas *Coprococcus* and *Dialister* are depleted in depression after adjustment for antidepressant use [405]. Causal involvement is supported by FMT experiments showing that transplantation of microbiota from MDD patients can induce depressive‐like behaviors in germ‐free mice [406]. Proposed mechanisms involve dysregulation of the hypothalamic–pituitary–adrenal axis, increased systemic and central inflammation, and metabolic alterations [407]. In a DSS‐induced colitis‐associated anxiety model, magnoflorine reshaped the gut microbiota and increased the microbiota‐associated bile acid HDCA, thereby alleviating anxiety‐like behaviors by suppressing microglia‐mediated neuroinflammation [408].

Microbial alterations have also been reported in schizophrenia (SCZ), where FMT from affected patients induces schizophrenia‐relevant behaviors and disrupts hippocampal neurochemistry, particularly the glutamate‐glutamine‐GABA cycle [409]. ASD models provide compelling experimental evidence for microbiome‐driven behavioral modulation; colonization of germ‐free mice with ASD‐associated microbiota induces social deficits and repetitive behaviors accompanied by altered microbial metabolite profiles and alternative splicing of ASD‐relevant genes in the brain [410]. Treatment with candidate neuroactive metabolites has been reported to ameliorate behavioral abnormalities in ASD models, indicating that microbial metabolites contribute to ASD‐like phenotypes [410].

### Section summary

The evidence reviewed supports the microbiome–gut–brain axis, within an organ‐like framework, as a dynamic bidirectional network that contributes to CNS homeostasis. Communication occurs through integrated neural signaling via the ENS and vagus nerve, microbial metabolites such as SCFAs and tryptophan metabolites, and immune‐mediated pathways that shape BBB integrity and neuroimmune cell function [345]. Dysregulation of the microbiota–gut–brain axis has been implicated in neurodegenerative diseases such as Alzheimer's disease and Parkinson's disease, as well as in psychiatric and neurodevelopmental conditions such as depression and autism spectrum disorder [351, 411].

Microbiota‐targeted interventions, including probiotics, prebiotics, synbiotics, and dietary strategies, represent promising but still developing therapeutic avenues [351]. Their clinical translation, however, requires deeper mechanistic understanding and identification of the key mediators that drive therapeutic effects, including specific microbial taxa, microbial functional capacities, metabolites, and host receptors [345, 412]. Integration of multi‐omics approaches with longitudinal human cohorts and mechanistic models will be critical to establishing causality and addressing inter‐individual variability. Development of personalized microbiome‐based interventions tailored to individual microbiome composition, metabolite profiles, immune status, and clinical phenotype represents an important future direction [412]. Continued exploration of the microbiome–gut–brain axis may ultimately yield novel biomarkers and adjunctive therapies, positioning gut health as a relevant component of neurological and psychiatric disease management.

## HOST FACTORS SHAPING THE MICROBIOME ORGAN

### Overview

**FIGURE 7:**
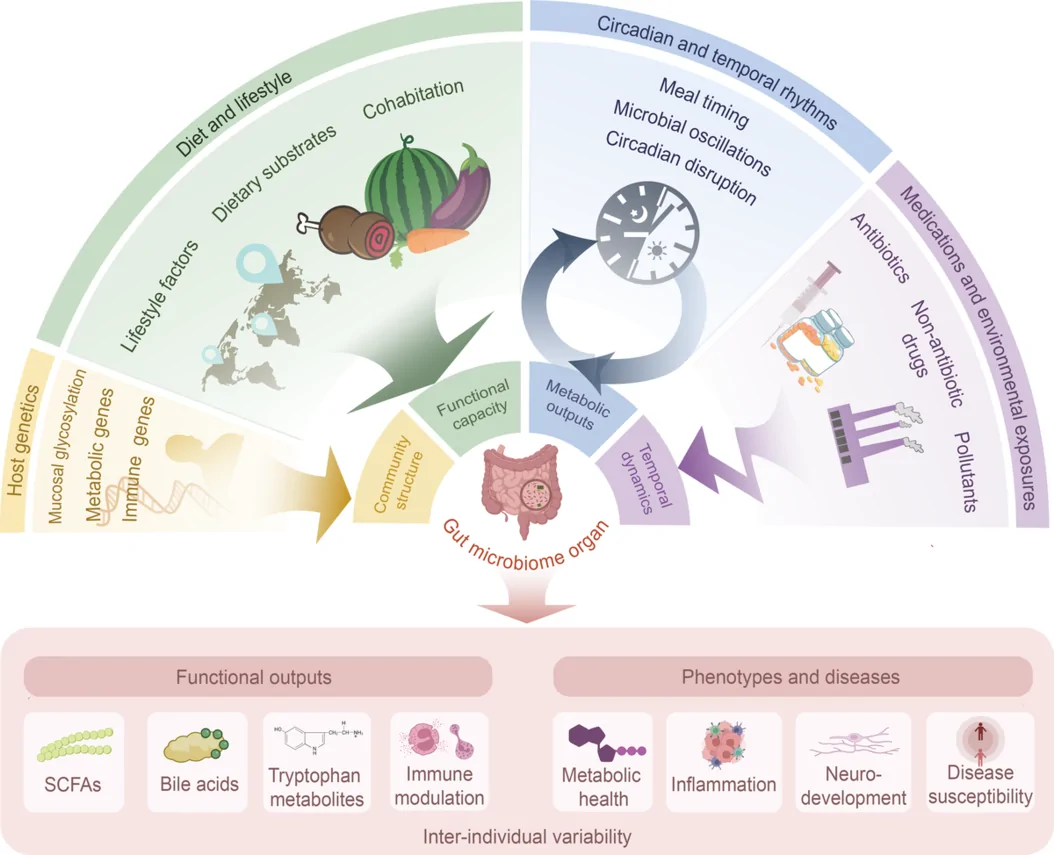
Host factors shaping the gut microbiome organ‐like system. Conceptual framework showing how host genetics, diet and lifestyle, circadian and temporal rhythms, medications, and environmental exposures shape the structure and function of the gut microbiome organ‐like system. These factors influence microbial community structure, spatial organization, functional capacity, and metabolic outputs, thereby contributing to inter‐individual variability in host physiology, disease susceptibility, and therapeutic response. The relative sizes of the sectors are conceptual and do not indicate effect size. SCFAs, short‐chain fatty acids.

The question of how host genetics and environmental factors shape microbial diversity between individuals and across time has been a central theme in microbiome research, echoing the classic nature‐versus‐nurture debate [413]. Early investigations yielded inconsistent answers; studies comparing the gut microbiota in human twins and across different inbred mouse lines often produced conflicting results regarding the extent of genetic control [20]. In contrast, candidate gene approaches in model organisms demonstrated that a single host gene can exert substantial effects on microbial diversity and population structure under defined experimental conditions [20]. Over the past decade, a clearer picture has emerged from large‐scale human cohort studies demonstrating that environmental factors, including cohabitation, diet, and medications, are major forces shaping the adult gut microbiome and often explain more variation than host genetics [414]. Genetically unrelated individuals sharing a household, as well as cohabiting relatives, harbor more similar microbiomes than non‐cohabiting individuals, strongly implicating shared environmental exposures as key drivers of microbial community structure [414]. Nevertheless, host genetics remain relevant; quantitative genetics have revealed that many microbial taxa are heritable, and specific host genetic variants have been linked to the abundance of particular microbial taxa or microbiome features [415]. A comprehensive understanding of the microbiome as a complex organ, therefore, requires moving beyond the simple host genetics–environment dichotomy to examine the dynamic interplay among host genetics, environmental inputs, and microbial ecology (Figure 7) [20]. This integrated view overlaps with the hologenome concept, which considers the combined genetic potential of the host and its associated microorganisms, and provides a conceptual framework for studying mechanisms underlying health and chronic disease [413].

### The host genetic contribution: Innate predispositions in microbiome assembly

While environmental exposures exert dominant effects, host genetic variation provides one layer of influence that can shape microbiome assembly and constrain aspects of its ecological configuration. Evidence from twin and family studies indicates that monozygotic twins can harbor more similar microbiomes than dizygotic twins, with host genetic effects varying across taxa [415]. However, microbiome heritability estimates are generally modest, and cohabitation and other shared environmental factors frequently explain a larger proportion of microbiome similarity than genetic relatedness [414]. These observations suggest that host genetics may establish a permissive framework within which environmental factors often act as stronger drivers.

Controlled studies in animal models reinforce the contribution of host genetics under defined conditions. Genetically diverse mouse populations, including the Collaborative Cross and advanced intercross lines, demonstrate that host genotype can account for substantial variation in microbial abundance under controlled environmental conditions. These systems have enabled mapping of host genetic loci that influence microbial traits, providing a basis for identifying candidate mechanistic links between host genotype and microbiome structure.

Genome‐wide association frameworks and microbial quantitative trait locus mapping have been used to associate host genetic variation with microbiome phenotypes, particularly the abundance of specific microbial taxa [20, 416]. Large‐scale efforts such as the MiBioGen consortium have improved statistical power by aggregating data across cohorts [417]. Nevertheless, progress in identifying robust and reproducible associations remains limited. Effect sizes are typically small, consistent with the polygenic nature of host genetic control, and many reported associations fail to replicate across populations due to differences in study design, microbiome‐profiling methods, sample size, and environmental context [418, 419]. In addition, the high dimensionality of microbiome data and the burden of multiple testing further constrain discovery. Emerging analytical approaches, including gene‐level and kernel‐based methods that capture beta‐diversity variation, aim to address these limitations by modeling more complex host–microbiome relationships [420].

Among the more consistently replicated findings are loci related to nutrient availability and mucosal interface biology. Variants at the lactase (*LCT*) locus, including lactase‐persistence variants, are associated with Bifidobacterium abundance, with dietary lactose intake acting as an important modifying factor [417, 419]. Similarly, *ABO* and *FUT2* loci regulate the expression of histo‐blood group antigens on mucosal surfaces, shaping microbial colonization by altering mucosal glycan availability as potential bacterial energy sources for taxa such as *Bifidobacterium bifidum and Collinsella* [419]. Mechanistic validation has been demonstrated in pigs, where a 2.3‐kb deletion in the porcine ABO ortholog reduced *N*‐acetyl‐galactosamine availability and consequently decreased the abundance of specific Erysipelotrichaceae taxa [421]. These examples illustrate how host genetics can influence microbiome assembly by controlling ecological resources and niche accessibility.

Host genetic effects also extend to immune regulation and barrier function. Several host variants associated with gut microbiota features occur in IBD‐related immune genes, particularly those involved in bacterial sensing and autophagy, such as *NOD2* and *ATG16L1* [422, 423]. These genes regulate microbial recognition, antimicrobial responses, and mucosal integrity, thereby shaping microbial community composition. For example, the protective *IL23R* rs11209026/R381Q variant has been associated with higher ileal microbial diversity and richness, as well as increased abundance of putatively health‐associated commensal taxa, particularly *Christensenellaceae* [424]. In addition, host pathways related to innate immunity, appetite control, and energy metabolism have been associated with gut microbiota composition, linking host physiological regulation to microbial ecology [425].

Despite these advances, several limitations complicate interpretation of host genetic influences on the microbiome. Replication of microbiome‐associated genetic loci remains inconsistent across studies, and identified variants typically explain only a small fraction of inter‐individual variation. Disentangling genetic effects from shared environmental exposures, such as diet and cohabitation, remains particularly challenging in human cohorts. Moreover, most studies rely on cross‐sectional designs, limiting the ability to assess temporal dynamics and causal relationships. These challenges highlight the need for integrative approaches that combine host genomics with longitudinal sampling, controlled environmental exposures, and functional validation.

Collectively, these findings indicate that host genetics contributes to microbiome assembly by shaping ecological niches, immune responses, and metabolic environments. However, its effects are context‐dependent and generally modest compared with environmental influences, reinforcing the need to consider genetic and environmental determinants within a unified framework when interpreting microbiome variation.

### The environmental input layer: Diet and lifestyle as major drivers

In contrast to host genetics, environmental exposures represent dynamic and modifiable drivers of microbiome composition and function. Large human cohort analyses indicate that environmental factors often account for a greater proportion of inter‐individual microbial variation than genetic background [426]. Cohabiting individuals often exhibit greater microbiome similarity, consistent with the influence of shared environmental exposures, while controlled mouse studies show that dietary interventions can override or reshape genotype‐dependent microbial differences [426, 427]. Across wild small mammals, shared habitat can promote partial convergence in gut microbiota composition, although species identity remains the dominant predictor, further indicating that the microbiome responds to external ecological inputs within host‐associated constraints [428].

Dietary intake represents an important and modifiable environmental determinant of microbial ecology [429]. Cooking of plant foods reshapes gut microbiome structure and function, partly through increased starch digestibility and degradation of heat‐sensitive plant‐derived compounds [430]. Microbial metabolism can also liberate bioactive molecules from dietary substrates, exemplified by *Bacteroides ovatus*‐associated liberation of N‐methylserotonin from orange fiber in experimental studies, which has been linked to adiposity, liver metabolism, and circadian regulation [431]. Similarly, tea polyphenol‐derived theabrownins can be shaped by microbial fermentation and may interact with gut microbiota‐derived metabolic pathways, thereby contributing to anti‐inflammatory and metabolic regulation [432].

Functional consequences of these interactions are captured through metabolomic profiling. The fecal metabolome reflects the metabolic interplay among microbial activity, host metabolism, and dietary substrates, providing a proximal functional readout of gut microbial and host co‐metabolic activity [433]. Gut microbial composition accounts for a substantial proportion of interindividual variation in fecal metabolite profiles in some cohorts. These profiles are linked to clinically relevant host phenotypes, such as visceral fat mass [433]. In IBD cohorts, they are also associated with fecal calprotectin‐defined intestinal inflammation [434]. Complementary to fecal metabolomics, urinary metabolomics captures absorbed and host‐processed host–microbial co‐metabolites, such as hippurate, phenylacetylglutamine, and indoxyl sulfate, providing a systemic readout of host exposure to microbiota‐derived metabolic products [4, 435]. Together, these approaches support mapping of metabolic relationships from dietary input to microbial transformation and host physiological response.

Importantly, metabolomic readouts reveal substantial inter‐individual variation in microbial functional capacity that is not fully captured by taxonomic composition alone. Even among individuals with similar microbial profiles, differences in gene expression, substrate utilization, and host–microbe co‐metabolic flux can lead to divergent metabolic outputs. These functional differences have been linked to clinically relevant phenotypes, including insulin resistance, chronic inflammation, and immune or neuroimmune regulation. Integration of microbiome sequencing with metabolomic profiling therefore provides a mechanistic framework linking environmental exposure to microbial function and host phenotype, with potential implications for personalized nutrition and microbiome‐targeted therapies.

Despite these advances, the relationship between lifestyle factors and the microbiome remains complex and often difficult to interpret. Dietary patterns, physical activity, medication use, and socioeconomic variables are frequently interrelated, introducing substantial confounding. In addition, most human studies remain cross‐sectional, limiting causal inference. These challenges highlight the need for controlled intervention studies, longitudinal cohort designs, and integrative analytical frameworks capable of disentangling environmental effects from host and microbial variability.

Collectively, these observations indicate that environmental factors shape the microbiome through dynamic, context‐dependent mechanisms that often exceed the influence of host genetics. This underscores the importance of integrating environmental and host determinants within a unified framework when interpreting microbiome variation and designing targeted interventions.

### The temporal dimension: Circadian rhythms and chronobiology

A bidirectional relationship exists between the host circadian system and the gut microbiota, introducing an important temporal dimension to host–microbe interactions [436]. Central and peripheral clocks, coordinated in part by the suprachiasmatic nucleus, generate approximately 24‐h rhythms in physiology and behavior [437]. These host rhythms coordinate metabolic, endocrine, and nutrient‐absorptive processes, thereby creating time‐dependent physiological conditions that shape daily oscillations in gut microbial composition and metabolite rhythmicity [436]. Reciprocally, rhythmic microbiota‐derived signals, including microbial metabolites, contribute to the temporal regulation of host circadian programs; homeostatic microbiome rhythmicity helps maintain normal transcriptional oscillations in peripheral tissues, including the intestine and liver [438]. This time‐dependent regulation forms an important component of the microbiota–gut–brain axis, through which microbial signals modulate hypothalamic–pituitary–adrenal (HPA)‐axis rhythmicity, stress responsiveness, and stress‐related brain pathways [439]. Disruption of this temporal dialog is increasingly implicated in diverse pathologies [436].

The gut microbiota can exhibit diurnal oscillations in bacterial load and taxonomic composition, with daily fluctuations evident at the levels of community diversity and genus‐level abundance [440]. These oscillations can involve daily fluctuations in numerous bacterial taxa and are paralleled by rhythmic changes in microbial metabolic pathways, including amino acid metabolism, bile acid transformation, and SCFA production [441]. SCFAs such as butyrate can act as epigenetic modulators that entrain circadian clock oscillations in IECs, thereby modulating local host circadian timekeeping [442]. Loss of microbial rhythmicity, marked by dampened or arrhythmic oscillations, has been linked to metabolic dysfunction, including type 2 diabetes [441]. Circadian–microbiota–immune dysregulation may also contribute to CVD, including atherosclerosis [436].

Among environmental zeitgebers, the timing of food intake represents a dominant synchronizing signal for both the host and the gut microbiota [443]. Time‐restricted feeding (TRF), which confines caloric intake to a defined daily window, can reinforce circadian oscillations and restore microbial rhythmicity in experimental and selected clinical settings [444]. In experimental colitis models, TRF has been reported to delay disease onset, attenuate intestinal inflammation, and improve rhythmicity in both the intestinal clock and the gut microbiota, highlighting the functional importance of rhythmic microbial states [445]. Meal timing therefore serves as a key regulator of peripheral circadian clocks and gut microbial rhythms, supporting metabolic homeostasis and host health.

Modern lifestyle‐related circadian disruption can arise when endogenous biological clocks become misaligned with environmental and behavioral schedules, including altered light exposure, food timing, jet lag, and shift work [436]. Such disruption may compromise gut barrier integrity and contribute to metabolic disease risk [446]. Chronodisruption has been associated with increased risk of metabolic, cardiovascular, neurodegenerative, and cancer‐related disorders. Abnormal food timing may promote metabolic dysfunction partly through gut microbial dysbiosis and impaired intestinal barrier integrity [447]. Increased intestinal permeability facilitates systemic exposure to gut‐derived microbial products, thereby promoting inflammation and metabolic dysregulation [447]. Dietary factors may exacerbate these effects; for example, high‐fat diets disrupt rhythmic REG3γ expression, dampen gut microbial oscillations, and promote metabolic dysfunction [448]. Collectively, these observations position the microbiome as a potential intermediary linking chronobiological disruption to disease‐relevant vulnerability [449].

### Exogenous perturbations: Pharmacological and environmental reshaping

Medication exposure represents an important exogenous determinant of microbiome variation, with antibiotics among the best‐characterized perturbants of gut microbial diversity and composition [450]. Antibiotic exposure can induce marked reductions in microbial diversity and substantial shifts in community composition, with recovery often incomplete and ecological networks sometimes remaining disrupted long after treatment cessation [451]. Early‐life antibiotic exposure may be particularly consequential, as sustained perinatal penicillin exposure in mice induces lasting gut microbiota alterations together with changes in brain cytokine expression, blood–brain barrier integrity, and behavior [452]. Antibiotic‐induced disruption of the gut microbiota can impair colonization resistance and increase susceptibility to enteric infection, particularly in experimental and high‐risk clinical contexts [453].

Beyond antibiotics, numerous non‐antibiotic medications influence microbial communities. Observational studies integrating microbiome data with electronic health records have identified associations between gut microbiome variation and several medication classes, including proton pump inhibitors, biguanides, beta‐blockers, antidepressants, and psycholeptics/benzodiazepine derivatives [454, 455]. Some medication‐associated effects may persist after treatment and may accumulate with repeated exposures [455]. These interactions have clinical relevance; in patients with psoriasis, antibiotic exposure has been associated with antibiotic‐induced gut dysbiosis proposed as one possible contributing mechanism [456]. Recognition of drug‐microbiome interactions underscores the importance of accounting for medication history as a potential confounder in microbiome studies and in the interpretation of microbiome data [455].

Environmental chemicals and pollutants further shape microbial ecology. Ingested particulate matter can alter microbial composition and immune responses in experimental studies, providing possible mechanistic links between air pollution and inflammatory diseases such as IBD [457]. Long‐term arsenic exposure has been associated with, and in experimental models can disrupt, intestinal barrier integrity, inflammation, gut microbial composition, and hepatic lipid metabolism [458]. Micro‐ and nanoplastics have emerged as additional concerns; animal studies indicate that ingestion can induce metabolic and neurobehavioral effects associated with microbiome disruption and altered circadian pathways [459]. Agricultural insecticides can reshape the gut symbiont communities of exposed insect pests and other model organisms, altering microbiota assembly and thereby influencing adaptive host phenotypes such as insecticide resistance [460].

Epidemiological evidence indicates that selected environmental toxicants in breast milk are associated with alterations in the developing infant gut microbiome, particularly during the critical early months of life. In the Norwegian Microbiota Cohort, breast milk PFOS and PBDE‐28 concentrations were associated with lower infant gut microbial diversity at 1 month, with PFOS also linked to β‐diversity differences and PBDE exposure associated with altered fecal SCFA profiles, suggesting potential associations with microbial composition and function [461]. Together with broader evidence linking early‐life nutritional exposure to environmental contaminants with gut microbiota development, immune responses, and metabolic regulation, these findings suggest that the infant gut microbiome may serve as a potential mediator or sensitive biosensor of environmental exposures [461, 462].

### The integrated response: Gene–environment–microbiome interactions

The composition of the gut microbiome reflects the integrated effects of host genetics and environmental exposures, particularly diet [463]. Although dietary interventions exert strong effects, their outcomes vary depending on host genotype. Studies in genetically diverse mice demonstrate that high‐fat/high‐sucrose diets induce broad microbiome shifts, yet strain‐specific microbial changes and obesity‐related metabolic phenotypes, particularly body fat gain, can be genotype‐dependent [463]. Similarly, microbial interventions may exhibit context‐dependent efficacy; therapeutic effects of *Eubacterium* species in osteosarcopenia differ across mouse strains, illustrating genotype‐dependent responsiveness [464]. Prominent taxa such as *Akkermansia muciniphila* may exert context‐dependent effects shaped by host genetics, diet, disease status, strain‐level variation, and surrounding microbial communities [465]. These findings emphasize the importance of gene–environment interactions in determining individual responses to dietary and microbial interventions.

Convergence of genetic susceptibility and dysbiosis can contribute to the pathogenesis of complex disorders. In IBD, many genetic risk loci influence host responses to the microbiome, including pathways involved in microbial sensing, autophagy, and immune cell differentiation [466]. Genetic predisposition may facilitate dysbiotic states, with reduced abundance of the butyrate‐producing genus *Roseburia* observed in healthy individuals carrying high IBD genetic risk [422]. Comparable principles apply in neurodevelopmental disorders; in *Cntnap2* (contactin‐associated protein‐like 2, an autism spectrum disorder‐associated gene) deficient mice, hyperactivity appears primarily driven by the genetic mutation, whereas social deficits are microbiome‐modulated and reversible through microbial intervention in this model [467]. These examples illustrate disease mechanisms arising from gene‐microbiome interplay rather than isolated genetic effects alone [468].

The intertwined relationship between host and microbiota underlies the concept of the holobiont, in which the host and associated microbial communities are considered an integrated ecological unit [469]. The hologenome encompasses the closely interacting genomes of the host and microbiome, framing host–microbe interactions as an integrated genomic and metagenomic system [413]. Health and disease can therefore be viewed as emerging, in part, from interactions within this host–microbiome system [470]. Experimental observations suggesting host genetic influences on stable microbiome community states support the broader view that host–microbiome interactions can have ecological and potentially evolutionary consequences [469]. This perspective may inform future microbiome‐based interventions by highlighting the need to consider host genetic, environmental, and microbial ecological context when interpreting host–microbe interactions [413].

### Section summary

Accumulating evidence has shifted understanding of microbiome determinants from reductionist models toward integrative systems biology frameworks that capture host genetic, environmental, and microbial complexity [471]. The field is progressing beyond descriptive associations toward mechanistic insights, yet key challenges remain, including establishing causality and translating findings from experimental models to clinical applications [471]. Methodological standardization and longitudinal cohort studies are required to better characterize host–microbiome interactions shaped by genetics, diet, medications, and environmental exposures [418]. Integration of multi‐omics data through advanced computational approaches will be important for modeling functional host–microbiome interactions and generating predictive frameworks [471].

Microbiome research is increasingly informing precision‐medicine approaches, as microbiome data can improve phenotype prediction in some settings and may support microbiome‐targeted interventions across diverse genetic backgrounds [426]. Future therapeutic strategies, including individualized dietary interventions, NGPs, rationally designed prebiotics, and fecal microbiota transplantation, will need to account for the unique biological context of each individual [471]. Careful use of the holobiont framework may provide a conceptual foundation for this transition, positioning the host and microbiome as an interacting biological system whose coordinated consideration may help assess and predict disease risks [469].

## MULTI‐OMICS MAPPING OF THE MICROBIOME ORGAN

### Overview

**FIGURE 8:**
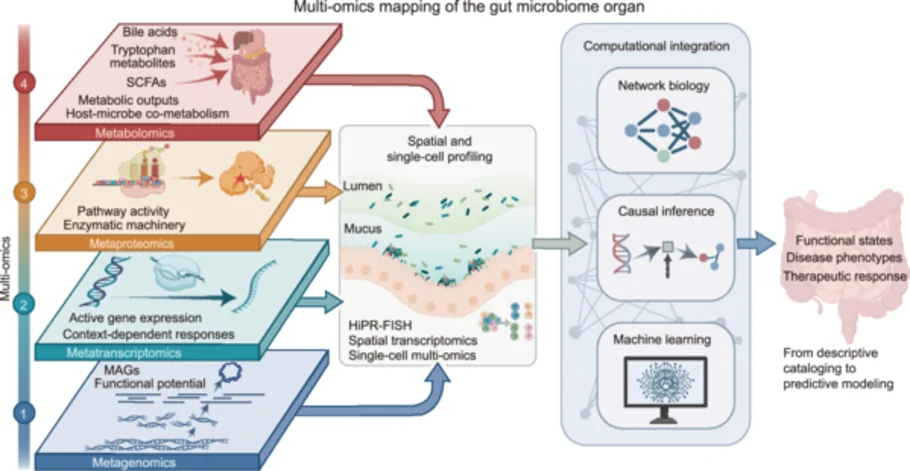
Multi‐omics and spatial mapping of the gut microbiome organ‐like system. Schematic showing how metagenomics, metatranscriptomics, metaproteomics, and metabolomics can be integrated with spatial host–microbiome profiling and host single‐cell approaches to map the functional architecture of the gut microbiome organ‐like system. These complementary layers capture functional potential, transcriptional activity, expressed proteins and enzymatic machinery, metabolic outputs, and host–microbe co‐metabolism. Computational integration through network biology, machine learning, and appropriately designed causal‐inference frameworks can support functional‐state inference, disease phenotype stratification, and therapeutic‐response prediction. Causal inference requires suitable study designs, such as longitudinal, perturbational, interventional, or genetically informed approaches, rather than cross‐sectional multi‐omics alone. HiPR‐FISH, high‐phylogenetic‐resolution fluorescence in situ hybridization; MAGs, metagenome‐assembled genomes; SCFAs, short‐chain fatty acids.

The conceptualization of the human microbiome as an organ‐like functional system supports a shift in research focus from taxonomic cataloging to a mechanistic understanding of its collective activities. While foundational studies based on 16S rRNA gene sequencing provided critical insights into the composition of microbial communities, this approach offers limited information about functional capabilities, strain‐level variation, or real‐time contributions to host physiology [472]. To move from descriptive community profiling toward predictive models of complex biological systems, the field has increasingly adopted a multi‐omics strategy that layers complementary molecular readouts (Figure 8).

Shotgun metagenomics provides the genomic layer, profiling microbial taxonomy and functional genes or pathways that outline the community's inferred functional potential [473]. Metatranscriptomics captures the transcriptional layer, revealing which genes are transcribed at a given time and in response to environmental or dietary perturbations [108, 474]. Metaproteomics offers closer evidence of functional machinery by identifying and quantifying microbial proteins, including enzymes and proteins related to metabolic pathways, that are actively expressed by microbial communities [475]. Metabolomics measures small‐molecule outputs shaped by microbial metabolism, host metabolism, diet, and host–microbe co‐metabolism that interface with host physiology [476]. The integration of these complementary data types, often described as systems biology or integrative omics, enables the construction of comprehensive and potentially predictive frameworks that may inform targeted therapeutic strategies [477].

### The genomic blueprint: Cataloging functional potential with metagenomics

Shotgun metagenomic sequencing represents a major advance beyond 16S‐based taxonomic profiling by providing a broad survey of the gene repertoire present within a microbial community. This approach estimates the functional potential of the gut microbiome and details the metabolic pathways represented by microbial functional genes [473]. Such analyses have revealed associations between disease contexts and alterations in microbial functional genes or pathways. In children with autism spectrum disorder, metagenomic profiling identified altered abundances of microbial functional genes related to folate biosynthesis, sulfur metabolism, and oxidative stress [473], whereas studies of metabolic syndrome have uncovered enrichment of pathways involved in carbohydrate and fatty acid biosynthesis [478]. Additional work has linked microbe‐mediated vitamin metabolism pathways to host health and disease [479], and associated pathway enrichments, such as amine/polyamine biosynthesis, glycerol degradation, and fermentation‐related pathways, with metabolic syndrome accompanied by elevated gamma‐glutamyl transferase levels [480]. These findings provide a functional context that is often absent from purely taxonomic surveys.

A longstanding challenge is the assignment of functional capabilities to specific microbial members or strains within complex communities. The development of metagenome‐assembled genomes (MAGs) has partly addressed this limitation by enabling the reconstruction of microbial genomes from metagenomic reads through computational assembly and binning [481]. MAG‐based analyses have provided strain‐resolved insights into disease processes; for example, in coronary artery disease, genome‐resolved MAG analysis revealed strain‐specific distributions of trimethylamine‐related genes, including TMA‐producing genes in *Anaerostipes hadrus* and *Megamonas funiformis* MAGs, linking microbial genomic variation to TMA/TMAO‐related metabolic potential [482]. Similarly, a MAG catalog generated from pig fecal samples helped attribute antimicrobial resistance genes to MAG‐level genomic sources, although ARG detection at the assembly or MAG level was lower than in read‐level analyses [481].

A recurrent theme in metagenomic studies is functional redundancy, wherein diverse taxa share genetic capacity to perform similar metabolic functions. This redundancy may help preserve aspects of microbiome function, as shifts in taxonomic composition do not necessarily translate into major alterations in predicted functional gene content [483]. In murine gut inflammation models, dramatic changes in the relative abundance of *Firmicutes*, *Bacteroidetes*, and *Proteobacteria* occurred without significant differences in inferred aggregate coding capacity, suggesting compensation among taxa with redundant genetic complements [483]. A longitudinal study in Crohn's disease remission patients similarly demonstrated persistence of core metabolic functions despite temporal variability in microbial composition and protein expression [484]. Together, these observations indicate that while taxonomic composition may fluctuate, the collective functional repertoire of the microbiome organ‐like system can remain comparatively conserved in some contexts.

### The transcriptional pulse: Capturing real‐time activity with metatranscriptomics

Whereas metagenomics estimates functional potential, metatranscriptomics provides a dynamic snapshot of community transcriptional activity by quantifying gene expression across microbial populations [474, 483]. The distinction between potential and expressed function is critical because gene presence does not imply transcriptional engagement. Comparative analyses show that metatranscriptomics captures transcriptional activity more directly than metagenomics alone and can provide a closer proxy for real‐time microbial metabolic activity [108, 483]. For instance, antibiotic resistance studies revealed that although metagenomes harbor a broad reservoir of resistance genes, metatranscriptomic profiling identified more targeted expression patterns in response to antibiotic treatment [485], underscoring its ability to reveal transcriptionally active biological processes [486].

This real‐time perspective makes metatranscriptomics particularly suitable for studying responses to dietary and environmental perturbations. In healthy individuals, shifts between high‐ and low‐glycemic‐load diets produced distinct transcriptional signatures, including altered hexitol fermentation pathways and differential expression of carbohydrate‐active enzymes reflecting dietary substrates [487]. In mice fed a Total Western Diet, broccoli powder supplementation induced dose‐dependent increases in transcripts linked to butyrate production and plant cell wall degradation [488]. Additional studies documented transcriptional responses to antimicrobials, such as phage‐related gene induction following carbadox exposure in pigs [489], and functional adaptations during pregnancy, where carbohydrate‐related gene expression was associated with the increased glucose availability characteristic of late pregnancy [490].

Metatranscriptomic profiling can also facilitate inference of regulatory networks and active metabolic interactions. In Parkinson's disease, gene co‐expression network analysis revealed depletion of hub genes and disruption of pathways including secondary bile acid biosynthesis and flagellar assembly, partly driven by reduced transcriptional activity attributed to *Blautia* and *Faecalibacterium* [491]. A study of defined *in vitro* gut communities identified active cross‐feeding interactions, such as acetate‐ and lactate‐driven butyrate production, that supported species coexistence and community‐level SCFA production [492]. Additional applications include characterization of stable transcriptional signatures associated with clinical outcomes following *S. Typhi* challenge [493] and discovery of widespread antisense transcription across gut bacterial species, revealing potential regulatory layers [494]. Collectively, these findings highlight metatranscriptomics as an important approach for mapping the active transcriptional landscape of the microbiome organ‐like system.

### The functional machinery: Direct evidence of microbial activity via metaproteomics

Metaproteomics provides protein‐level evidence of microbial function by identifying and quantifying microbial proteins and related metabolic pathways [475, 495]. By measuring microbial proteins, including enzymes and transport‐related proteins, this approach complements metagenomic and metatranscriptomic profiling, provides a closer measure of microbial functional status, and enables enzyme‐level characterization of microbial activity [496]. Analyses of healthy gut communities have revealed a stable core metaproteome enriched in proteins involved in central metabolic pathways including glucose and pyruvate metabolism, with butanoate metabolism and butyrate production also prominently represented [475].

Metaproteomic studies have revealed microbiota responses to dietary perturbations. For example, fecal metaproteomics from a controlled refined‐grain, high‐glycemic‐load dietary intervention showed enrichment of enzymes associated with human mucin degradation, suggesting increased microbial mucus‐glycan utilization under this dietary pattern in that study [496]. Investigations of dietary protein sources similarly reported changes in proteins involved in amino acid degradation and breakdown of protein‐conjugated glycans [495].

A notable strength of metaproteomics is the capacity to profile functional enzyme classes central to microbiome–environment interactions. Analyses of CAZymes have identified genus‐associated enzyme repertoires, with *Prevotella*‐rich communities expressing plant polysaccharide‐degrading CAZymes and *Bacteroides*‐rich communities showing a partially distinct CAZyme profile [497]. An in vitro synthetic‐community study of mucin degradation revealed cooperative enzyme contributions from specialists such as *Akkermansia muciniphila* and generalists, including *Bacteroides* spp., illustrating ecological interactions driven by enzyme secretion and metabolite cross‐feeding [498]. Metaproteomics has also shown enrichment of mucin‐degrading enzyme activity from the *Ruminococcus torques* group in individuals with chronic *Strongyloides stercoralis* infection, suggesting infection‐associated modulation of microbial activity [499].

To refine functional interrogation, activity‐based protein profiling (ABPP) and related chemoproteomic approaches have been introduced for targeted identification of enzyme activities. These methods employ activity‐based probes to enrich and characterize functionally active enzymes within complex metaproteomes [500]. ABPP has identified soluble‐fiber‐responsive sugar‐metabolizing enzymes in the mouse gut and linked activity of the *Bifidobacterium* shunt pathway to soluble fiber intake [500]. Activity‐based metaproteomics has further facilitated the discovery and characterization of gut microbial enzyme activities, including novel α‐galactosidases with potential industrial applications [501]. Chemoproteomic profiling using activity‐based probes has also characterized bile salt hydrolases across microbial taxa and delineated substrate preferences that contribute to host bile acid pool diversity [502].

### The chemical dialog: Decoding host–microbiome crosstalk through metabolomics

Metabolomics provides a proximal functional readout of the host–microbiome ecosystem by capturing small‐molecule metabolites involved in microbiome‐metabolome crosstalk and microbial‐host interactions [476]. Some of these metabolites function as effectors of microbial or host–microbial metabolism and mediate downstream physiological effects on the host. Profiling circulating metabolomes, particularly plasma metabolites, in combination with gut microbiome composition and clinical traits enables identification of metabolic signatures associated with physiological and pathological states and facilitates exploration of microbiome–host interactions that may underlie gut–organ communication axes [503]. Integrative analyses have linked coordinated changes in the gut microbiome and plasma metabolome to malignant pleural effusion, implicating dysregulated sphingolipid metabolism and steroid hormone biosynthesis [504]. Exploration of the gut–brain axis has suggested that microbiota‐derived extracellular vesicles can transport neuroactive molecules, including glutamate and GABA, potentially contributing to gut–brain axis signaling [505]. Similarly, microbiome‐metabolome alterations have been reported in the gut–kidney axis in IgA nephropathy [506] whereas gut microbiome dysbiosis and altered microbial metabolic pathways have been implicated in the gut–bone axis in Modic changes [507].

A central analytical challenge in metabolomics is resolving the origin of metabolites detected in systemic biofluids. The measured metabolome reflects a composite of host‐derived compounds, microbial products, dietary metabolites, and molecules generated through host‐microbial co‐metabolism [508]. Untargeted metabolomics platforms capture broad chemical diversity, detecting thousands of endogenous features as well as xenobiotics and their biotransformation products in a single analysis [509]. Mechanistic dissection of metabolite origins is therefore important. A representative study combining stable isotope tracing with gnotobiotic mouse models demonstrated a host–microbe co‐metabolic pathway for hippuric acid production, in which gut bacteria convert dietary phenylalanine into phenylpropionic acid, which undergoes host β‐oxidation via medium‐chain acyl‐CoA dehydrogenase (MCAD, an enzyme involved in fatty acid β‐oxidation) before glycine conjugation and urinary excretion [508]. Such examples highlight how microbial metabolism can generate bioactive intermediates that impact pathogenesis; production of tyramine by *Enterococcus faecium*, for instance, has been implicated in intestinal injury and MASLD‐related pathology in experimental or disease‐associated contexts [510].

The fecal metabolome thus serves as an integrated readout associated with gut microbial functions, host inputs, diet, and disease‐related metabolic alterations [511]. Comparative analyses in cerebral ischemic stroke revealed that fecal metabolite profiles correlated more strongly with gut microbial composition than plasma or urine metabolomes, suggesting fecal metabolites may provide a closer proxy for gut‐local microbial activity in that context [511]. However, urinary metabolomics has been shown in other contexts to provide a valuable non‐invasive functional readout of gut microbial activity [512], particularly because some gut‐derived metabolites are systemically absorbed, host‐modified, and excreted in urine. In certain infections, urine can provide a stronger readout of microbial metabolic activity [513]. Urinary metabolites such as hippurate, phenylacetylglutamine, and other microbial‑derived compounds are often used as biomarkers of microbiome‑host co‑metabolism because they integrate microbial activity with host absorption and processing, and urinary profiles can be more stable or less variable across sampling conditions than fecal metabolite profiles in some study designs. Emerging causal inference frameworks further evaluate metabolites as potential intermediaries linking environmental exposures to host phenotypes. Serum metabolite profiling has demonstrated mediation of lifestyle effects on cardiometabolic health through compounds such as cinnamoylglycine and secondary bile acids influenced by microbial taxa [514]. Similar causal analyses in anorexia nervosa indicate that serum bacterial metabolites may mediate the effects of altered gut microbiota on AN‐related behavioral traits [515]. Collectively, these observations support metabolomics as an important modality for translating microbial community features into measurable physiological outcomes.

### Resolving microbiome architecture through spatial and single‐cell approaches

The functional output of the microbiome organ is shaped by its spatial organization, a dimension obscured by bulk sequencing of homogenized samples. The gastrointestinal tract contains heterogeneous microenvironments spanning the lumen, mucus layers, and epithelial surfaces, each characterized by distinct physicochemical conditions and microbial assemblages. High‐resolution mapping approaches are therefore required to resolve these spatial relationships. A key advance has been high‐phylogenetic‐resolution microbiome mapping by fluorescence in situ hybridization (HiPR‐FISH), which uses binary encoding, spectral imaging, and machine learning‐based decoding to generate micrometer‐scale, single‐cell maps of the locations and identities of hundreds of microbial taxa in situ in selected communities [516]. Application of HiPR‐FISH has enabled visualization of structured microbial biogeography in settings such as the mouse gut and human oral plaque, revealing organized spatial networks at host‐microbe interfaces.

Spatial mapping studies indicate that microbiome architecture can be dynamic and responsive to perturbations. Antibiotic exposure disrupts established microbial spatial networks in the mouse gut, altering spatial associations among genera and community structure [516]. Prior to multiplexed imaging approaches, spatial heterogeneity was inferred through compartmental sampling. For example, metaproteomic comparisons of luminal and mucus‐associated communities in the rat intestine demonstrated distinct species distributions and location‐specific enzyme repertoires, including variation in carbohydrate transport and energy conservation pathways [517]. These findings emphasize that fecal samples may not fully capture metabolic processes occurring at mucosal surfaces where host–microbe interactions are often most pronounced.

To integrate spatially resolved molecular layers, computational frameworks such as Multi‐modal Alignment of Genes and Peaks for Integrated Exploration have been developed to co‐register spatial transcriptomics with mass spectrometry imaging‐based spatial metabolomics data [518]. This integration enables simultaneous mapping of gene expression, metabolite distributions, and tissue morphology, providing a multi‐modal representation of tissue‐level processes, although it is not yet microbiome‐specific [518]. Advances toward single‐cell resolution are further extending analytical depth. Single‐cell simultaneous metabolome and transcriptome profiling (scMeT‐seq) allows correlation of metabolite levels with gene expression within individual cells by sub‐picoliter cytoplasmic sampling followed by single‐cell RNA sequencing [519]. Although currently applied primarily to host or cultured cells, such approaches establish conceptual and methodological foundations for future microbial or host–microbe single‐cell analyses, potentially offering higher‐resolution views of functional heterogeneity within microbial communities.

### Synthesizing the system: Integrative computational frameworks for a holistic view

The breadth and complexity of microbiome‐inclusive multi‐omics datasets require appropriate analytical and integration strategies to generate coherent systems‐level interpretations [477]. Central to this effort is the construction of multi‐omic interaction networks that model relationships among microbial taxa, genes, proteins, metabolites, host transcripts, and clinical traits [520, 521]. These networks can be derived using diverse integration frameworks, including correlation‐based methods, Bayesian network inference, and multi‐layer (multiplex) network models that explicitly capture cross‐omic dependencies. In particular, approaches such as similarity network fusion, canonical correlation analysis, and matrix factorization enable the integration of heterogeneous data types into unified representations, while graph‐based methods facilitate the identification of candidate hubs, modules, and cross‐domain interactions.

Network‐based analyses can provide a systems‐level view of how perturbations may propagate across biological layers, revealing coordinated changes rather than isolated effects. For example, integrative profiling in schizophrenia linked gut microbial alterations to serum metabolomic shifts and inflammatory cytokine alterations, highlighting multi‐layered dysregulation [522]. Similarly, integrative studies have identified age‐related changes in oral–gut microbial associations and linked gut microbial signatures to host lipid profiles and fecal metabolite features [523]. Analytical platforms such as MicrobiomeAnalyst and Analysis of Pan‐Omics Data in Human Interactome Network can facilitate these analyses by providing integrated pipelines for microbiome or metabolome association analysis and pan‐omics network/pathway interpretation, respectively [520, 521]. Functional annotation through databases such as KEGG and GO can link network‐derived features or modules to biological pathways and functional processes [524].

A major interpretative challenge is distinguishing correlation from causation within complex microbiome–metabolome interaction networks [525]. To address this, causal inference methodologies are increasingly applied. Mendelian randomization leverages germline genetic variants as instrumental variables to estimate potential causal effects of modifiable exposures, including microbial taxa or metabolites, on disease outcomes while reducing confounding under specific assumptions [526]. This approach has been used to examine putative causal relationships among host genetics, microbial features, metabolites, and allergic diseases [527]. Metabolic modeling approaches integrated with multi‐omics data offer another route for testing mechanistic hypotheses and, in constraint‐based frameworks, generating causal or mechanistic predictions in microbiome–metabolome relationships [528, 529]. Emerging machine‐learning approaches for multi‐omics integration further enable the discovery of candidate molecular signatures and disease‐relevant patterns in complex microbiome data sets [530].

Predictive modeling using machine learning and artificial intelligence constitutes an additional component of integrative microbiome systems biology and precision medicine [530, 531]. Training algorithms on multi‐omics datasets can enable the construction of models for disease classification and outcome prediction. Metatranscriptomic signatures evaluated using Random Forest, Support Vector Machine, and XGBoost models predicted immunotherapy outcomes in non‐small cell lung cancer with promising predictive performance in the studied cohort [532]. Integrative AI models incorporating plasma proteomics, metabolomics, and gut metagenomics have also been applied to predict Alzheimer's disease severity [533]. Beyond biomarker discovery, such models can highlight informative features and generate hypotheses for biological validation; for example, tree‐based machine learning approaches identified candidate microbial strains and metabolites with therapeutic potential in ulcerative colitis, with these candidates subsequently validated in an experimental UC mouse model [534]. Together, network modeling, causal inference, and predictive analytics are supporting increasingly mechanistic interpretations of microbiome multi‐omics data.

### Section summary

The study of the microbiome is transitioning from descriptive taxonomic characterization toward systems‐level investigation, driven by the integration of high‐throughput omics technologies [472]. Metagenomics estimates the genetic repertoire underlying microbial functional potential, while metatranscriptomics and metaproteomics provide complementary insights into actively expressed functions. Metabolomics captures small‐molecule outputs that mediate host–microbiome crosstalk, linking microbial activity to host physiology [476]. Spatial and emerging single‐cell approaches further extend this framework by revealing how physical organization within the gut niche may shape community function [516].

The principal strength of this multi‐omics paradigm lies in its capacity for integrative analysis. Computational frameworks combining network biology, causal inference approaches such as Mendelian randomization, and machine learning can help construct models that move beyond association toward functional interpretation, provided that assumptions are tested and findings are experimentally validated [477, 525, 530]. Together, these computational and machine‐learning approaches facilitate identification of candidate biomarkers and pathways linking microbiome perturbations to disease phenotypes or treatment outcomes, although their predictive and causal value remains context‐dependent [532, 534]. By incorporating responses to perturbations such as diet, therapeutic interventions, and disease states, multi‐omics integration can provide a foundation for developing strategies aimed at rational modulation of microbiome function [472, 535].

Despite these advances, significant challenges remain. Technical variability related to sample collection, processing, transport, storage, and methodological choices can affect comparability across microbiome studies [536]. Translating high‐dimensional datasets into coherent biological insight remains a major bottleneck, and many reported associations lack functional validation. These limitations highlight a persistent gap between data generation and mechanistic understanding. Addressing this gap will require standardized methodologies, improved computational integration, transparent reporting, external validation, and systematic experimental validation.

## THE MICROBIOME ORGAN IN HUMAN DISEASE

### Overview

**FIGURE 9:**
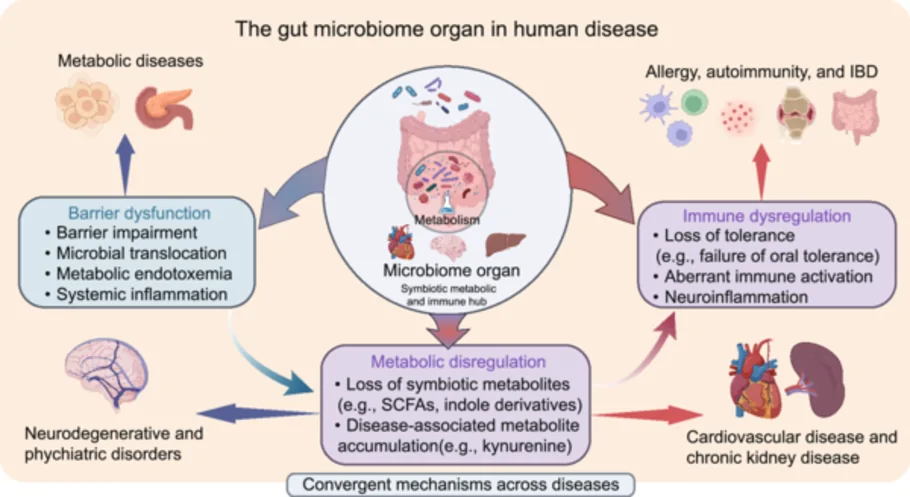
Convergent microbiome‐related mechanisms across human diseases. Conceptual model showing how perturbation of microbiome structure or function may be associated with disease‐related phenotypes through three convergent mechanisms: intestinal barrier dysfunction, metabolic dysregulation, and immune dysregulation. These processes may involve impaired epithelial barrier integrity, microbial translocation, systemic inflammatory tone, loss of symbiotic metabolites, altered host–microbe co‐metabolism, dysregulated bile acid and tryptophan metabolism, impaired immune tolerance, and aberrant immune activation. Together, these shared mechanisms provide a framework for linking microbiome perturbation with metabolic, cardiovascular, kidney, neurological, psychiatric, autoimmune, and tumor‐associated phenotypes. Th1/Th17: T helper 1/T helper 17 cells.

The gut microbiome contributes to systemic homeostasis through bidirectional interactions with the host, collectively referred to as gut–X axes [346, 537]. Disruption of this system, commonly termed dysbiosis, is increasingly recognized as a potential contributor or disease‐associated feature across a wide range of human diseases, including metabolic, inflammatory, immune‐related, and neurological disorders (Figure 9) [537].

Through the production of bioactive metabolites, modulation of immune responses, and regulation of host metabolic pathways, the microbiome exerts systemic effects that extend beyond the gastrointestinal tract [346, 538]. Perturbations of these functions contribute to disease pathogenesis by altering energy balance, immune homeostasis, and neuroendocrine signaling [537, 539]. Elucidating the mechanisms by which microbiome dysfunction contributes to disease is therefore important for the development of targeted diagnostic and therapeutic strategies.

### Breaching the boundary: Intestinal barrier dysfunction as a common pathogenic feature and mediator

The intestinal epithelial barrier serves as a critical interface, separating the host's internal milieu from the dense microbial and antigenic contents of the gut lumen. Its integrity is important for supporting systemic homeostasis, and its compromise is implicated in the pathogenesis of numerous diseases [540]. Dysfunction of this barrier has been reported in disorders of gut–brain interactions, such as irritable bowel syndrome (IBS), as well as in IBD and colorectal cancer (CRC) [541, 542]. Pathogenic mechanisms that undermine this barrier are multifaceted. In IBD, for instance, altered intestinal epithelial glycosylation, characterized by an increase in truncated O‐glycans, disrupts the protective mucus layer, which normally helps segregate microbes from the epithelial surface [543]. This structural failure is often compounded by the formation of dense mucosal biofilms, particularly in subsets of IBS and ulcerative colitis patients; these biofilms are associated with dysbiosis and overgrowth of taxa such as *Escherichia coli* and *Ruminococcus gnavus* [544]. Furthermore, genetic disruption or reduced expression of *SLC26A3* (DRA) can impair epithelial tight junction protein expression, leading to increased paracellular permeability [545]. Pathobiont‐associated taxa, such as adherent‐invasive *Escherichia coli* (AIEC) and, in specific contexts, *Bacteroides vulgatus*, can exploit or exacerbate these weaknesses, thereby promoting epithelial barrier dysfunction, microbial or microbial‐product translocation, and inflammatory mucosal responses [546, 547]. High‐fat diets can also promote tumorigenesis in experimental CRC models by impairing barrier function and promoting the growth of pro‐tumorigenic or pathobiont‐associated bacteria [548]. These convergent mechanisms of barrier failure, which span genetic susceptibility, altered mucus architecture, biofilm formation, and pathobiont activity, result in the inappropriate exposure of the host immune system to microbial components, potentially triggering local and, in some contexts, systemic inflammation.

The consequences of a compromised intestinal barrier extend far beyond the gastrointestinal tract, potentially promoting systemic inflammation that has been linked to a wide array of non‐communicable diseases. A key mechanism driving this systemic effect is the translocation of microbial components, most notably LPS, an outer‐membrane component of Gram‐negative bacteria also known as endotoxin, from the gut lumen into circulation [389]. This gut‐derived LPS exposure can contribute to chronic low‐grade inflammatory signaling and metabolic dysfunction, including obesity‐related adiposity and insulin resistance [549]. Elevated circulating LPS can trigger inflammatory pathways in metabolic organs such as the liver and adipose tissue, impairing insulin signaling and promoting adiposity [550]. This pathway is not restricted to metabolic diseases; the endotoxin hypothesis of neurodegeneration proposes that chronic systemic inflammation fueled by gut‐derived LPS may contribute to the pathogenesis of Alzheimer's disease, Parkinson's disease, and amyotrophic lateral sclerosis [389]. Gut‐derived endotoxins can activate peripheral immune cells and, through blood‐brain barrier‐related inflammatory signaling, promote the activation of microglia [389, 551]. This neuroinflammatory response is a common feature of many neurodegenerative conditions, where microglial activation is linked to synaptic loss, neuronal damage, and pathological protein aggregation, including α‐synuclein pathology in PD [389, 552]. Therapeutic strategies that restore barrier integrity, such as IL‐22‐based approaches, have been shown in experimental contexts to decrease endotoxemia and ameliorate metabolic dysfunction and mucosal immune deficits, supporting a mechanistic link between impaired gut barrier function, endotoxemia, and systemic metabolic disease [191].

### Metabolic dysregulation: Altered microbial bioenergetics and signaling pathways

The gut microbiome functions as a dynamic metabolic and signaling organ, producing and modifying a broad array of metabolites, some of which enter systemic circulation and act as signaling molecules to regulate host physiology [151, 537]. Dysregulation of these microbial metabolic pathways is a common feature of many chronic diseases and may contribute to disease progression. Among the most extensively studied microbial products are SCFAs produced through the fermentation of dietary fibers [187]. These molecules are key regulators of energy homeostasis and insulin sensitivity [187, 553]. SCFAs signal through GPCRs such as GPR43/FFAR2 and GPR109A/HCAR2 on various host cells, including enteroendocrine cells, immune cells, and adipocytes, thereby influencing gut hormone secretion, reducing inflammation, and modulating adipose tissue function [554]. Dysbiosis in metabolic syndrome can be characterized by reduced abundance or activity of SCFA‐producing taxa, which may contribute to impaired glucose homeostasis and insulin resistance [555]. Butyrate, for instance, enhances mitochondrial activity and can improve insulin sensitivity, while propionate and acetate also contribute to metabolic regulation [553, 555].

Microbial transformation of primary into secondary bile acids represents another key component of bile acid homeostasis [556]. These metabolites signal through receptors such as FXR and TGR5 to regulate systemic glucose, lipid, and energy metabolism [105, 556]. Perturbation of this pathway can have metabolic consequences; for example, oral vancomycin reduces Gram‐positive bacteria, decreases secondary bile acid levels, and has been associated with decreased peripheral insulin sensitivity in subjects with metabolic syndrome, supporting a mechanistic link between microbiota‐dependent bile acid metabolism and host metabolic regulation [557].

Emerging evidence implicates gut dysbiosis in blood pressure regulation and hypertension‐related phenotypes through multiple interconnected pathways. SCFAs can modulate vascular tone and renal function via receptors such as GPR41/FFAR3, GPR43/FFAR2, and OLFR78 [558], and their depletion may contribute to blood pressure dysregulation. In parallel, dysbiosis may promote pro‐inflammatory immune responses, including altered Th17/Treg balance, contributing to vascular inflammation and endothelial dysfunction [559]. Microbial metabolites and circulating endotoxins may further interact with the renin–angiotensin–aldosterone system, potentially influencing vasoconstriction and sodium retention. Together, impaired SCFA production and disrupted bile acid signaling represent metabolic disturbances that may contribute to cardiometabolic risk, while interacting with immune and vascular mechanisms linking gut microbial imbalance to hypertension.

Beyond their role in energy homeostasis, gut microbes are pivotal in the metabolism of amino acids, with tryptophan catabolism representing an important nexus for neuroimmune and metabolic regulation [560]. Tryptophan is metabolized through three major routes: the serotonin pathway, the kynurenine pathway, and the microbial indole pathway [561]. Gut dysbiosis can alter the balance among these pathways, with important consequences for host physiology. In obesity and chronic inflammatory states, for example, indoleamine 2,3‐dioxygenase (IDO), the rate‐limiting enzyme of the kynurenine pathway, is often upregulated [192]. This may shift tryptophan availability and metabolism away from microbial indole derivative production [192]. This IDO‐driven shift in tryptophan metabolism may contribute to metabolic dysfunction, while increased IDO activity is also linked to immunosuppressive effects and adverse cardiovascular outcomes [192]. Conversely, inhibition of IDO activity or its genetic deletion has been reported in experimental settings to improve metabolic health by rewiring tryptophan metabolism toward microbiota‐dependent indole/AhR–IL‐22 signaling, thereby preserving gut barrier integrity and reducing systemic inflammation [192, 562]. The gut–brain axis can also be affected by aberrant tryptophan metabolism. In depression, alterations in microbial tryptophan catabolism have been linked to an imbalance between indole derivatives such as IAld and ILA, which may differentially modulate neuroplasticity in the nucleus accumbens, a brain region linked to depression [563]. Specific gut microbiota‐derived tryptophan metabolites, such as IPA, have also been shown to exert protective effects beyond intestinal and neuroimmune contexts; for example, IPA alleviates atherosclerosis by promoting reverse cholesterol transport [138]. This divergence of tryptophan catabolism may therefore represent one mechanism of immune miscommunication and metabolic dysregulation in the host that contributes to chronic disease.

The metabolic output of the gut microbiome is not always beneficial; under dysbiotic conditions, it can generate metabolites that may promote disease progression, a phenomenon particularly evident in CVD [564]. Beyond the extensively studied TMAO, other microbiota‐derived co‐metabolites contribute to these diseases. PAGln is a microbial–host co‐metabolite derived from dietary phenylalanine, in which microbial species convert phenylalanine to phenylacetic acid, subsequently conjugated with glutamine by the host. PAGln has been shown in experimental and clinical association studies to promote or associate with adverse cardiovascular phenotypes through adrenergic receptor signaling, enhancing platelet reactivity and thrombosis, and correlates with increased risk of major adverse cardiovascular events in humans [196]. Beyond cardiovascular outcomes, microbial amino acid metabolites including PAGln may influence metabolic and neuroendocrine pathways, potentially contributing to insulin resistance, obesity, and hypertension via sympathetic activation, inflammation, and vascular dysfunction [565]. This positions PAGln alongside other microbial or microbial–host co‐metabolites, such as TMAO, SCFAs, and secondary bile acids, which translate microbial metabolism into systemic physiological effects, highlighting the broader role of the gut microbiota in cardio‐metabolic and neuroendocrine disease. Similarly, imidazole propionate, a microbial metabolite of histidine, has been linked to insulin resistance and has more recently been associated with atherosclerosis and Parkinson's disease‐related pathology [566, 567]. The generation of these potentially detrimental metabolites may represent one metabolic signature of microbiome dysfunction that contributes to the pathogenesis or progression of cardiometabolic and CKDs.

### Immune miscommunication: From local dysregulation to systemic and neuro‐inflammation

The intestinal mucosa is a site of constant, dynamic interaction between the host immune system and the gut microbiome, where a regulated balance must be maintained to promote tolerance to commensals while retaining the ability to fight pathogens. Dysbiosis disrupts this equilibrium, leading to immune dysregulation that begins locally but can escalate to systemic pathology [537]. An important cell type in this process is the ILC3, which contributes to intestinal homeostasis. ILC3s help maintain the epithelial barrier and regulate host‐microbiota symbiosis through the production of cytokines like IL‐22 [191, 568]. The function and plasticity of ILC3s are shaped by signals from the microbiota and are dysregulated in inflammatory conditions such as IBD [569]. In colitis, disruption of the ILC3/ILC1 balance can be associated with plasticity from ILC3s toward pro‐inflammatory ILC1‐like states contributing to disease pathology [569]. This local dysregulation extends to the adaptive immune system, disrupting the balance between pro‐inflammatory Th17 cells and regulatory T cells, which is essential for maintaining mucosal tolerance [253]. The failure of mucosal tolerance, a fundamental process whereby the mucosal immune system learns to ignore harmless antigens from food and commensal bacteria, is a feature of this immune miscommunication and may contribute to the development of IBD and systemic autoimmune diseases [570, 571]. This breakdown allows for aberrant immune responses against gut bacteria, leading to chronic mucosal inflammation, as seen in IBD, and potentially seeding systemic autoimmune reactions [466, 570]. CTLA‐4, an immune checkpoint expressed on regulatory lymphocyte populations and reported in some ILC contexts, plays a key role in maintaining intestinal homeostasis in a microbiota‐dependent manner, highlighting how immune regulatory pathways are intertwined with microbial signals to prevent inflammatory disease [572].

The consequences of immune dysregulation originating in the gut can extend to the central nervous system, establishing a gut–brain inflammatory axis that is increasingly implicated in neurodegenerative and psychiatric disorders [539]. In Parkinson's disease models, alterations in the gut microbiome appear to be functionally linked to selected disease‐related pathologies [395]. Preclinical studies using animal models that overexpress α‐synuclein, the protein that aggregates in PD, have shown that the gut microbiota can contribute to motor deficits, microglial activation, and α‐synuclein pathology [395].

Mechanistically, this process may involve systemic inflammation arising from impaired intestinal barrier integrity, which permits translocation of microbial components such as LPS into circulation and subsequent activation of immune pathways that influence brain function [573]. In addition, microbiota‐derived metabolites, including SCFAs (e.g., acetate and butyrate), have been demonstrated to drive microglial activation and worsen motor phenotypes in α‐synuclein‐overexpressing mouse models, supporting a metabolite‐mediated link between the gut microbiome and neuroinflammation [395].

The gut–brain immune axis is also implicated in neurodevelopmental disorders such as ASD. Individuals with ASD have frequently been reported to exhibit gut dysbiosis, altered intestinal permeability, and altered immune profiles, including elevated pro‐inflammatory cytokines. These changes are associated with impaired mucosal barrier function and enhanced translocation of microbial components, which may contribute to systemic immune activation and influence neurodevelopmental processes. Microbiota‐derived metabolites further contribute to this axis; for example, alterations in SCFAs and tryptophan metabolism have been linked in experimental models to synaptic function, microglial activity, and behavioral phenotypes. Experimental studies provide mechanistic support; microbiota modulation can ameliorate selected social and behavioral deficits in animal models of ASD. Collectively, these findings suggest that immune and metabolic dysregulation originating in the gut may contribute to aberrant neurodevelopment through sustained neuroimmune signaling [345, 410].

The gut–brain inflammatory axis may also be relevant to mood disorders, including depression and anxiety. Dysbiosis has been associated with increased intestinal permeability, chronic low‐grade systemic inflammation, and elevated pro‐inflammatory cytokines, all of which have been linked to depressive phenotypes in subsets of patients or experimental models [574]. These peripheral inflammatory signals may influence the CNS and are associated with microglial activation and synaptic dysfunction [551, 575]. For instance, microbial imbalances can alter tryptophan metabolism, shifting tryptophan availability away from serotonin synthesis and toward the kynurenine pathway, which generates neuroactive metabolites linked to depressive symptoms [576]. Collectively, these findings suggest that immune miscommunication involving the gut microbiome may propagate neuroinflammatory processes that contribute to depressive phenotypes in specific contexts.

### Impaired host–microbiome symbiosis in allergic and autoimmune disease

The establishment of host–microbiome symbiosis during early life is an important determinant of immune system maturation and the development of tolerance. Disruptions to this process, particularly within the first few years of life, are increasingly linked to higher risk of allergic diseases such as atopy and asthma [577]. The hygiene hypothesis and its modern iterations posit that reduced exposure to a diverse range of environmental microbes impairs the proper education of the developing immune system, predisposing individuals to inappropriate inflammatory responses later in life [578]. This is particularly evident in allergic sensitization and asthma, where neonatal gut dysbiosis is a recurrent finding in at‐risk infants [577]. Specific metabolic signatures of this dysbiosis have been identified; for example, elevated fecal concentrations of the lipid metabolite 12,13‐dihydroxy‐9Z‐octadecenoic acid (12,13‐diHOME), produced by bacterial epoxide hydrolases in the neonatal gut, have been shown to impair Treg cell development and promote pulmonary inflammation in preclinical models [577]. This provides mechanistic support for a link between an altered early‐life microbiome, its metabolic output, and the failure of immune tolerance that underlies allergic disease. The maternal microbiome may also play an important role in this process. Vertically transmitted bacteria, such as specific *Lactobacillus* species from the vaginal microbiome, may influence the infant's immune development and IgE status, suggesting a microbial component to the intergenerational transmission of atopy risk [333]. Conversely, exposure to diverse microbial communities, such as those found in soil, can support immune tolerance and alleviate allergic‐type inflammation in mouse models, reinforcing the importance of robust microbial inputs for healthy immune development [331].

The breakdown of a symbiotic host‐microbiome relationship is also a potential contributing factor in the pathogenesis of autoimmune diseases, where immune tolerance to self‐antigens is impaired, leading to an attack on the body's own tissues [570]. This process, known as the breakdown of peripheral tolerance, can be exacerbated and, in some contexts, triggered by alterations in the gut microbiota. Commensal microbes produce a range of symbiosis factors, including metabolites like SCFAs and secondary bile acids, which contribute to immune homeostasis and tolerance [571, 579]. A reduction in these potentially beneficial microbes and their metabolic products can create a pro‐inflammatory environment that facilitates the activation of self‐reactive immune cells [578]. This mechanism is thought to contribute to a variety of autoimmune conditions, including IBD, multiple sclerosis, and rheumatoid arthritis, many of which show patterns of gut dysbiosis and intestinal barrier dysfunction [570]. Furthermore, genetic predisposition plays a critical role, as autoimmune risk alleles often function in pathways that regulate lymphocyte activation and immune tolerance [580]. For example, certain genetic variants can disrupt inhibitory signaling pathways in B lymphocytes, making them more susceptible to activation by environmental or inflammatory cues, thereby contributing to the breakdown of immune tolerance [580]. Emerging evidence has also raised the possibility that autoimmune mechanisms contribute to Parkinson's disease, where the gut microbiome may modulate the activity of autoreactive T cells that are hypothesized to drive neurodegeneration, further highlighting the potential systemic and organ‐specific immune consequences of lost symbiosis [403].

### Microbiome‐associated carcinogenesis: From dysbiosis to tumorigenesis

The microbiome organ‐like system may contribute to cancer development, particularly in CRC, where dysbiosis has been linked to tumor initiation and progression through interconnected mechanisms involving barrier dysfunction, chronic inflammation, and metabolic reprogramming [542, 548]. Disruption of the intestinal barrier can permit sustained exposure of the epithelium to microbial products and genotoxins, promoting DNA damage and pro‐tumorigenic signaling pathways [542]. Specific pathobionts have been implicated in CRC pathogenesis; for example, Fusobacterium nucleatum promotes tumor progression through activation of β‐catenin signaling and modulation of anti‐tumor immunity, while certain pks‐positive strains of Escherichia coli produce colibactin, a genotoxin that can induce DNA damage, including double‐strand breaks [542, 543].

Microbial metabolites further shape the tumor microenvironment. While SCFAs such as butyrate can exert anti‐tumor effects through epigenetic regulation and maintenance of epithelial integrity, dysbiotic states may reduce their production and favor the accumulation of pro‐inflammatory, genotoxic, or pro‐tumorigenic metabolites, thereby shifting the balance toward tumorigenesis [496, 548, 555]. In parallel, chronic microbiota‐driven inflammation, characterized by persistent activation of innate and adaptive immune pathways, can create a microenvironment that supports tumor growth, angiogenesis, and immune evasion [542, 565].

A prototypical example of microbe‐driven carcinogenesis is provided by *Helicobacter pylori*, which is causally linked to gastric cancer. Persistent colonization induces chronic inflammation, epithelial damage, and progressive histological changes culminating in malignancy. This paradigm illustrates how long‐term host‐microbe interactions can drive cancer development through combined inflammatory, metabolic, and genomic mechanisms [578].

Collectively, these findings position the microbiome as a potential participant in carcinogenesis, integrating environmental exposures, host genetics, and immune responses to influence cancer risk and progression. Targeting microbiome composition and function, therefore, represents a promising but still developing avenue for cancer prevention, risk modification, and therapy.

### Section summary

The diverse pathologies discussed throughout this section, though manifesting in different organ systems, are frequently associated with a common set of core dysfunctions involving the microbiome organ‐like system. Three convergent mechanisms repeatedly emerge across these conditions. First, breakdown of intestinal barrier integrity can allow translocation of microbial products such as LPS, contributing to chronic, low‐grade systemic inflammation that has been linked to metabolic, cardiovascular, neurodegenerative, and neoplastic diseases [191, 389]. Second, metabolic dysregulation may occur, characterized by the depletion of beneficial symbiosis factors, such as SCFAs and tryptophan‐derived AhR ligands, alongside increased production of potentially detrimental metabolites such as TMA, TMAO‐related co‐metabolites, PAGln, imidazole propionate, and pro‐inflammatory kynurenine pathway products [187, 192]. This metabolic imbalance may impair host energy homeostasis, vascular health, and neuroimmune communication. Third, these dysfunctions can culminate in immune miscommunication, where the loss of microbial cues for tolerance can contribute to dysregulated mucosal immunity, impaired education of the developing immune system, and the emergence of local and systemic inflammatory, allergic, and autoimmune phenotypes [539, 570, 577]. These three pillars, namely, breached barriers, metabolic dissonance, and immune miscommunication, are not independent but are deeply interconnected, creating feed‐forward cycles that may perpetuate disease. Adopting a systems‐level perspective is therefore essential for understanding human health and disease [537, 581]. This view positions the microbiome organ‐like system as an integrative interface that helps translate environmental inputs, such as diet, and host genetic factors into physiological responses that can affect multiple organ systems. Recognizing these convergent mechanisms of dysfunction opens new avenues for developing trans‐disease therapeutic strategies aimed at restoring symbiotic microbiome functions to support systemic homeostasis.

## THERAPEUTIC TARGETING OF THE MICROBIOME ORGAN

### Overview

**FIGURE 10:**
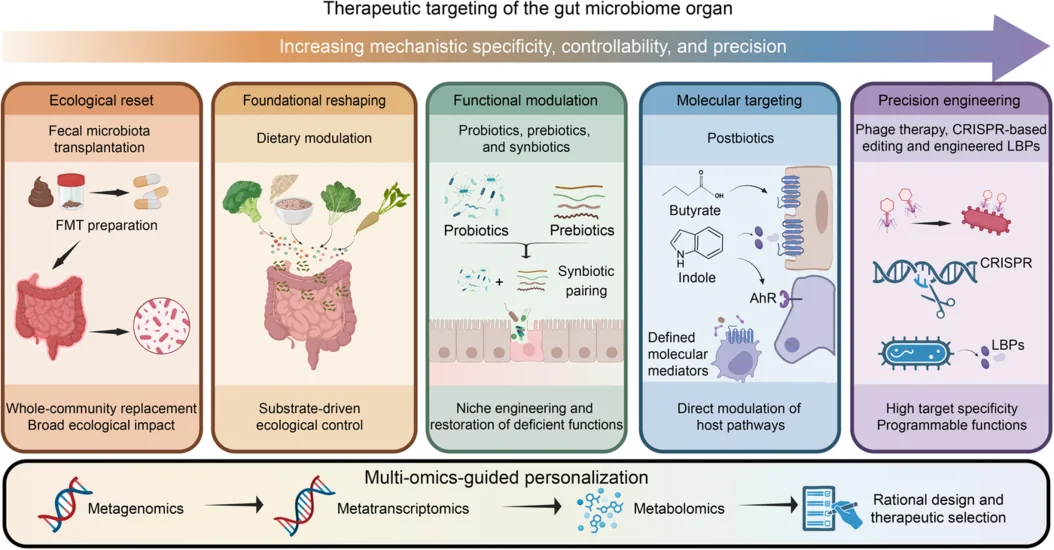
Therapeutic strategies targeting microbiome function. This schematic illustrates a conceptual continuum of microbiome‐directed interventions, ranging from broad ecosystem‐level approaches to increasingly defined and potentially programmable strategies. Therapeutic modalities include broad ecological restructuring through fecal microbiota transplantation, substrate‐driven modulation through diet, functional modulation using probiotics, prebiotics, and synbiotics, molecular targeting with postbiotics or microbial‐derived metabolites, and emerging precision approaches such as phage therapy, microbiome‐targeted CRISPR‐based editing, and engineered live biotherapeutic products. Across this continuum, interventions generally move toward greater mechanistic specificity, controllability, and personalization, whereas reliance on broad ecosystem perturbation tends to decrease. This gradient denotes conceptual specificity and controllability, not clinical maturity or proven efficacy. Multi‐omics‐ and phenotype‐guided personalization may support patient stratification, rational therapeutic selection, response monitoring, and iterative optimization of microbiome‐directed therapies. AhR, aryl hydrocarbon receptor; CRISPR, clustered regularly interspaced short palindromic repeats; FMT, fecal microbiota transplantation; LBPs, live biotherapeutic products; SCFAs, short‐chain fatty acids.

The gut microbiome possesses extensive genetic and functional capacity and plays key roles in digestion, immune maturation, and systemic metabolism, positioning it as a complex but potentially druggable therapeutic target [582, 583]. The development of microbiome‐targeted therapies reflects a gradual shift from empirical, broad‐spectrum interventions toward more mechanism‐informed strategies. Early approaches, such as FMT, operate on the principle of whole‐community transfer, an ecologically broad intervention that is highly effective for preventing recurrence of Clostridioides difficile infection but less predictable in other indications [584, 585]. However, the inherent variability and undefined nature of such treatments have catalyzed the development of more refined therapeutic modalities [584, 586]. This has led to a tiered framework of interventions that may offer increasing levels of definition and precision. These strategies range from ecological resets via whole‐community transplantation and foundational reshaping through diet, to functional modulation using probiotics, prebiotics, and synbiotics to support specific microbial guilds [587, 588]. At a more granular level, molecular targeting with defined microbial metabolites, or postbiotics, allows for direct engagement with host signaling pathways, bypassing the need for microbial engraftment [587, 589]. At the most targeted end of this therapeutic spectrum is precision editing, which employs tools like bacteriophages and engineered live biotherapeutics to selectively deplete detrimental strains or introduce defined functions into the gut ecosystem (Figure 10) [590, 591, 592]. This progression from broad ecological intervention toward rational design underscores a maturing field that may support more personalized and programmable microbiome therapeutics.

### Ecological reset and community‐level reshaping

#### Whole‐ecosystem transplantation: The case of FMT

FMT represents a broad community‐level approach to ecological reset, transferring a complex donor microbial community with the aim of restoring ecological functions disrupted by dysbiosis [584, 593]. Its clinical success in preventing recurrence of *Clostridioides difficile* infection (rCDI) exemplifies its therapeutic potential, acting as a broad microbiota‐restoration strategy that replaces a compromised ecosystem with a healthy one [584, 594]. Mechanistically, FMT's efficacy is thought to involve engraftment of donor microbiota, which restores critical ecological functions such as colonization resistance against pathogens [594, 595]. This is achieved through multiple synergistic actions, including the restoration of secondary bile acid metabolism, which can suppress *Clostridioides difficile* germination and growth, and the replenishment of SCFA‐producing taxa, which may support gut barrier repair and modulate host immunity [594, 596, 597]. For instance, FMT can restore levels of valerate, a metabolite depleted by antibiotics that has been reported to inhibit *Clostridioides difficile* growth [598]. Despite its clinical efficacy in rCDI and exploratory or mixed results in other conditions such as IBD and graft‐versus‐host disease (GvHD), FMT is characterized by low predictability and significant mechanistic uncertainty [593, 599, 600]. The therapeutic outcome is highly dependent on donor variability, recipient niche factors, and the undefined nature of its active constituents, making it challenging to standardize [585, 601]. Donor strain engraftment is variable and can be influenced by the recipient's pre‐FMT microbial diversity, with more severe dysbiosis sometimes permitting greater donor‐strain engraftment [595, 602]. Concerns regarding the transfer of pathogens or undesirable traits, alongside the incompletely defined nature of whole‐stool transplants, have highlighted the need for more defined and safer alternatives [584, 585, 586]. While FMT has been a critical proof‐of‐concept for microbiome therapy, its limitations underscore the necessity of moving toward strategies with greater control and predictability.

#### Foundational reshaping through dietary intervention

Dietary intervention offers a foundational and generally non‐invasive approach for reshaping the gut microbiome by directly targeting substrate availability and modulating microbial community structure and metabolic output [603, 604]. Unlike the abrupt replacement characteristic of FMT, diet can induce gradual regulation of community structure and metabolic output, influencing SCFA production, bile acid metabolism, and tryptophan metabolism [605, 606]. For example, consumption of grain proteins from sources such as highland barley and oats improved glucose metabolism in HFD‐fed mice by enriching *Lactobacillus* and *Bifidobacterium* and increasing gut microbiota‐derived indole metabolites such as indole‐3‐ethanol, which can activate host AhR signaling [604]. Conversely, a high‐fat, low‐fiber Western‐style diet promotes dysbiosis, reducing taxonomic diversity and impairing the microbiome's resilience to perturbations like antibiotic treatment [606, 607]. This diet‐induced dysbiosis can promote tumorigenesis in experimental contexts, partly through altered butyrate metabolism, a key fuel for colonocytes and a modulator of macrophage polarization [607]. Evidence from murine models suggests that an appropriate dietary environment can be sufficient in some settings for microbiome recovery after antibiotics, and may outperform microbial transplants in driving this process in specific experimental settings [606]. This research challenges the assumption that FMT is a universal solution for dysbiosis, demonstrating that a regular, fiber‐rich diet can promote syntrophic cross‐feeding interactions that support community recovery, whereas a Western diet allows dominant taxa to monopolize resources without supporting the broader ecosystem [606]. While the impact of diet is high, its controllability is dependent on host adherence and the baseline microbiota, making it a potentially powerful but personalized tool for foundational microbiome reshaping.

### Modulating key functional guilds and metabolic output

#### Restoring deficient functions with probiotics and NGPs

Probiotics and NGPs represent a potentially more targeted approach to functional modulation, aiming to restore or augment specific beneficial taxa or functional guilds rather than replacing the whole microbial community [608, 609]. This strategy moves beyond broad ecological resets to introduce organisms with proposed or demonstrated strain‐specific health‐promoting capabilities. Classical probiotics, such as certain strains historically assigned to *Lactobacillus* and *Bifidobacterium*, have been shown in selected contexts to modulate host health by enhancing gut barrier integrity, producing antimicrobial compounds, and modulating immune responses [610, 611]. The field is now advancing toward NGPs, which include commensal organisms whose therapeutic potential has been more recently identified, such as the mucin‐degrader *Akkermansia muciniphila* and the major butyrate‐producing commensal *Faecalibacterium prausnitzii* [612, 613]. Low levels of Faecalibacterium are frequently associated with inflammatory conditions such as IBD, while *Akkermansia muciniphila* has been associated with improved metabolic health and enhanced responses to cancer immunotherapy in selected cohorts and experimental models [612, 614]. These NGPs may augment specific functions, such as barrier enhancement, immune modulation via TLR2 activation, and production of important metabolites such as SCFAs [613, 615]. However, the controllability and predictability of these interventions remain limited. The efficacy of probiotics can be highly context‐dependent, influenced by successful engraftment, which is often transient, and by complex interactions with the recipient's resident ecosystem [616, 617]. The therapeutic benefits of a probiotic strain can be highly specific and are not always generalizable to the entire species, necessitating rigorous strain‐level characterization to realize their full potential as LBPs [609, 612].

#### Niche engineering with prebiotics and synbiotics

Prebiotics and synbiotics offer a more refined strategy for modulating microbial function by modifying the metabolic environment of the gut to selectively favor the growth and activity of desired microbes [589, 618]. Prebiotics are substrates, including dietary fibers and oligosaccharides, which are selectively utilized by host microorganisms and confer health benefits [619]. This approach creates a competitive advantage for specific endogenous or exogenously administered bacteria, thereby potentially achieving targeted modulation of microbial populations and their metabolic functions. A well‐studied example of this niche modulation is the use of human milk oligosaccharides (HMOs), which create a selective niche for *Bifidobacterium longum* subsp. *infantis* [616, 620]. This specific pairing forms the basis of a rational synbiotic, defined as a mixture comprising live microorganisms and substrate(s) selectively utilized by host microorganisms that confers a health benefit on the host. Studies have demonstrated that co‐administering *Bifidobacterium longum* subsp. *infantis* with HMOs can lead to predictable and high‐level engraftment in some adult microbiomes, a feat that is difficult to achieve with the probiotic alone and without antibiotic pre‐treatment [616]. The dose of prebiotic can be used in model systems to achieve tunable control over the steady‐state abundance of the probiotic and some metabolic outputs, while related in vivo and in vitro models also show augmented butyrate levels through cross‐feeding interactions with resident *Firmicutes* [616, 620]. This synbiotic approach can improve the predictability of probiotic engraftment and function in selected contexts, moving the field closer to precision engineering of the microbiome [620]. By providing a selective metabolic advantage, synbiotics may help mitigate colonization resistance and support the establishment and functional activity of administered biotherapeutics in selected contexts [618, 621, 622].

### Targeting microbial–host signaling with defined molecules

#### Postbiotics: Bypassing the ecosystem to directly modulate host pathways

Postbiotics are preparations of inanimate microorganisms and/or their components that confer a health benefit; purified microbial metabolites are a distinct intervention class [587, 589]. This approach may reduce some complexities associated with live microbial administration and colonization, offering a way to more directly modulate host physiology [587]. The core targets of postbiotic and metabolite‐based interventions are often host cellular receptors and signaling pathways responsive to microbial cues. These include GPCRs, nuclear receptors such as FXR, and transcription factors such as the AhR [604, 623]. By delivering defined microbial components or metabolites that mediate selected beneficial effects of microbiota, postbiotics may provide better‐defined composition and greater controllability than live microbial approaches [587]. For example, rather than administering a butyrate‐producing bacterium and relying on successful colonization and metabolic activity, one can directly supply butyrate or derivatives to engage host GPCRs and HDAC‐related pathways [589, 624]. This approach may reduce variability related to the recipient's resident microbial community and support more standardized dosing. The use of postbiotics and metabolite‐based interventions may transform some microbiome‐inspired therapies from complex ecological interventions into more controllable pharmacological approaches, providing a direct means to influence host immune and metabolic responses [589, 625].

#### Key metabolite classes as therapeutic targets

Several classes of microbial metabolites have emerged as therapeutic candidates or targets because they can exert local or systemic effects on host physiology [624]. Produced from the fermentation of dietary fibers, SCFAs serve as an important link between diet, the microbiome, and host health, influencing inflammation, barrier function, and gut–brain communication [626, 627]. Butyrate, for instance, not only provides energy for colonocytes but also strengthens the intestinal barrier and promotes the differentiation of regulatory T cells, thereby maintaining immune homeostasis [607, 626]. Secondary bile acids, such as deoxycholic acid and lithocholic acid, are another important class of signaling molecules produced through microbial transformation of primary bile acids from the liver. These metabolites can signal through bile acid‐sensitive receptors such as TGR5/GPBAR1, VDR, PXR, and, depending on bile acid species and concentration, FXR, regulating lipid metabolism, glucose homeostasis, and inflammation, and can contribute to colonization resistance against pathogens such as *Clostridioides difficile* [594, 596]. Furthermore, microbial metabolism of dietary amino acids generates a diverse array of bioactive molecules. Tryptophan‐ and phenylalanine‐derived metabolites are particularly important for modulating the gut–brain axis and immune responses [604, 628]. Selected indole derivatives produced from tryptophan can activate AhR, which contributes to intestinal barrier integrity and regulation of neuroinflammation [604, 623, 628]. Conversely, metabolites such as phenylethylamine, derived from phenylalanine by specific bacteria, such as *Ruminococcus gnavus*, can accumulate in pathological states such as liver cirrhosis and contribute to neurotoxicity, highlighting the dual role of these pathways in health and disease [629].

### Precision editing of the microbiome organ for therapeutic function

#### Strain‐specific depletion via phage therapy and CRISPR‐Cas systems

Precision editing of the gut microbiome aims to target specific pathogenic or detrimental bacterial strains, or even their virulence genes, with minimal disruption to the broader commensal community [591]. This approach contrasts with broad‐spectrum antibiotics, which cause widespread collateral damage to the ecosystem. Bacteriophages (phages), viruses that specifically infect bacteria, are natural predators that can be harnessed for this purpose. Phage therapy can offer high specificity, as phages typically target a narrow range of bacterial hosts, allowing for the selective removal of undesirable elements such as pathobionts or antibiotic‐resistant organisms [591]. This strategy may help address some donor variability and safety concerns associated with FMT by creating more defined phage‐ or virome‐based preparations [586]. The precision of this approach can be further enhanced by combining phages with CRISPR‐Cas systems. Engineered phages can be used as delivery vehicles to introduce programmable CRISPR‐Cas machinery into target bacteria in situ [591, 630]. This technology may support strain‐specific depletion of target cells or targeted genomic editing, with potential future applications aimed at genes encoding virulence factors or antibiotic resistance determinants [630]. While the theoretical precision of these methods is attractive, significant challenges remain, including efficient delivery to the complex gut environment, the potential for off‐target effects, and the rapid evolution of bacterial resistance mechanisms [591, 630].

#### In situ functional augmentation with engineered LBPs

Beyond depletion, targeted microbiome engineering also encompasses augmentation of functional capacity through engineered LBPs. This strategy involves genetically modifying well‐characterized commensal bacteria, such as Escherichia coli Nissle 1917 (EcN), to perform novel and specific therapeutic functions directly within the gut [592, 631, 632]. Rather than replacing a missing function with a natural probiotic, this approach adds a controlled, programmable activity to the existing ecosystem. For example, engineered EcN has been developed to treat metabolic diseases by expressing pathways that degrade toxic metabolites. In a mouse model of hereditary tyrosinemia type 1 (HT1), an engineered EcN strain capable of degrading tyrosine prevented lethal liver injury in a mouse model by reducing the accumulation of toxic metabolic byproducts [631]. Similarly, for Parkinson's disease, EcN has been engineered to synthesize l‐DOPA from l‐tyrosine, providing a programmable gut‐based drug delivery platform that improved motor performance in animal models [632]. This approach offers the potential for controlled, localized therapeutic delivery, as the engineered bacteria can be designed to respond to specific environmental cues or be regulated by orally administered inducers [592, 633]. The development of tools like insulated genomic landing pads and genetic circuits allows for stable integration and tunable control of therapeutic gene expression, with the aim of reducing metabolic burden and improving strain performance [592]. Despite its substantial potential, this field faces significant hurdles, including ensuring the genetic stability and fitness of engineered strains in the competitive gut environment, achieving controlled payload release or colonization, and navigating standardization and regulatory pathways for clinical translation [590, 592].

### Section summary

The therapeutic targeting of the microbiome has undergone substantial development, progressing from broad ecological interventions toward more defined molecular and genetic engineering approaches. This trajectory began with whole‐ecosystem resets like fecal microbiota transplantation, a broad but incompletely predictable method that demonstrated the therapeutic potential of modifying microbial communities [593, 594]. The recognition of its limitations, such as donor variability and safety concerns, spurred the development of more defined and controlled approaches. Foundational reshaping through dietary interventions highlighted the critical role of substrate availability in sculpting the microbiome, in some experimental settings proving more effective than transplantation for microbiome recovery [606]. The subsequent focus on functional modulation via probiotics, prebiotics, and synbiotics allowed for the targeted support of specific beneficial microbial guilds, with synbiotics in particular offering improved control over probiotic engraftment by engineering the metabolic niche [616, 620]. A further increase in definition came with the use of defined microbial products, inanimate microbial preparations, or metabolite‐based interventions, which can partly bypass reliance on live microbial engraftment and directly engage host signaling pathways, potentially improving product consistency [587]. At the forefront of this evolution lies the capacity for precision editing, where technologies such as phage therapy and CRISPR‐Cas systems may enable selective depletion or editing of detrimental strains, and engineered live biotherapeutics introduce novel, programmable functions directly into the gut [630, 631, 632]. This layered toolkit underscores the value of a multi‐level targeting strategy, where the choice of intervention is guided by a mechanistic understanding of the specific dysbiosis and host pathology in question [588]. The future of microbiome therapeutics lies in integrating these diverse strategies within a framework of personalized medicine. By leveraging multi‐omics diagnostics, it may become possible to profile a patient's microbial and metabolic state with greater accuracy to guide the rational design of tailored interventions [585, 590]. This data‐driven approach may enable improved selection of FMT donors, the formulation of individualized probiotic, prebiotic, or synbiotic combinations, or the deployment of precisely engineered biotherapeutics, supporting the development of more defined, programmable, and predictable microbiome‐based medicine.

## THE TRANSLATIONAL FRAMEWORK: BUILDING CAUSAL EVIDENCE BEYOND ASSOCIATION

### Overview

**FIGURE 11:**
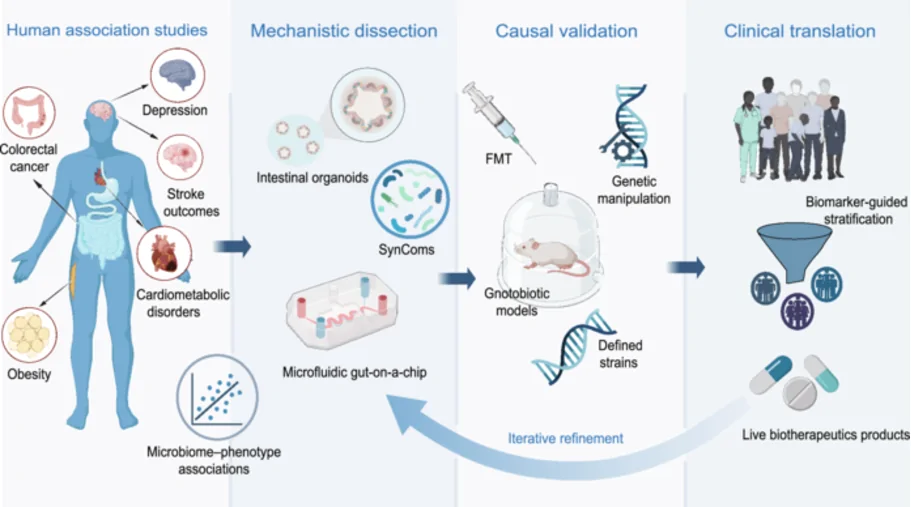
Translational framework for microbiome research. This framework illustrates the progression from human discovery and association studies to mechanistic analysis in controlled experimental systems, followed by causal validation in animal or gnotobiotic models and biomarker‐guided clinical translation. Mechanistic dissection can involve intestinal organoids, synthetic microbial communities, microfluidic gut‐on‐a‐chip systems, metabolite or receptor assays, and immune or epithelial co‐culture models. Causal validation may include human‐to‐mouse microbiota transfer, defined strains or SynComs, gnotobiotic models, and host or microbial genetic manipulation. Clinical translation requires biomarker‐guided patient stratification, clinical testing, safety assessment, and longitudinal response monitoring. SynComs, synthetic microbial communities.

A central challenge in microbiome research is the transition from identifying correlations to establishing causation [634]. The scientific literature contains numerous associations linking alterations in the gut microbiota, commonly described as dysbiosis, to a wide spectrum of human diseases, including obesity, CRC, and cardiometabolic disorders [634, 635]. However, these associative studies, while important for hypothesis generation, are limited in their ability to establish mechanistic or causal links. They cannot, on their own, determine whether observed microbial changes are a cause of disease, a consequence of underlying pathophysiology, a mediator, or a bystander effect [635]. This ambiguity represents an important bottleneck, hindering the translation of promising findings into reliable clinical applications [635, 636]. The use of human microbiota transplantation into gnotobiotic rodents has become an important approach for probing causality, yet the high rate of reported phenotype transfer in these models may overstate the microbiome's causal role in complex human disease and warrants cautious interpretation [635]. To navigate these complexities and ensure the scientific credibility of the field, there is a need for a structured, multi‐tiered translational framework. Such a framework should systematically guide research from initial clinical observations through mechanistic dissection in controlled systems, rigorous causal validation in preclinical models, and ultimately, to well‐designed human interventional studies (Figure 11) [634, 636]. Adopting this approach is important for avoiding unrealistic expectations and supporting the development of effective microbiome‐based diagnostics and therapeutics [635].

### Hypothesis generation and mechanistic dissection in controlled in vitro systems

The initial deconstruction of complex host–microbiome interactions can be supported by sophisticated in vitro systems that bridge the gap between clinical observation and in vivo validation. Patient‐derived organoids have emerged as a useful tool, creating three‐dimensional epithelial models that preserve individual‐specific genetics and aspects of epithelial architecture, thereby enabling personalized research [637]. These systems allow controlled testing of interactions between patient‐specific microbiota or microbial products and intestinal epithelial or organoid‐based host models, providing a platform to screen individualized epithelial responses to microbiota–drug interactions in cancer therapy [638]. Complementing organoids, microfluidic gut‐on‐chip platforms offer a dynamic environment that can more closely approximate selected aspects of intestinal physiology, including peristalsis‐like mechanical forces, host–microbiome interactions, and selected mucosal immune‐related readouts [639]. Advanced gut‐on‐chip models can integrate different cell types and model selected aspects of inter‐organ communication, such as the gut–liver axis, allowing researchers to study how gut microbiota‐derived metabolites and extracellular vesicles affect hepatocyte function in a connected liver chamber [640]. These platforms are particularly valuable for co‐culturing the host epithelium with complex communities or highly oxygen‐sensitive anaerobic bacteria, such as *Faecalibacterium prausnitzii*, under physiologically relevant low‐oxygen or anoxic luminal conditions, which are difficult to achieve in conventional cell culture [641]. Dynamic systems such as the Simulator of the Human Intestinal Microbial Ecosystem (SHIME) model can also simulate the digestive process and gut microenvironment across the stomach, small intestine, and different colon regions, enabling the identification of microbially transformed food‐derived metabolites under controlled conditions [642]. These controlled environments facilitate mechanistic screening of specific microbial metabolites and their effects on epithelial barrier‐related and immune‐related readouts [637].

Parallel to modeling the host, understanding the intricate ecological dynamics within the microbial community itself is critical. Synthetic microbial communities (SynComs) provide a reductionist, bottom‐up approach to dissect microbe–microbe interactions [643]. By assembling defined consortia of sequenced gut isolates, researchers can systematically dissect phenomena such as competition for dietary substrates (e.g., fructose), or cooperative cross‐feeding where the metabolic byproducts of one species, such as lactate or formate, serve as nutrients for another [644]. Such strain‐resolved approaches have helped map the contributions of individual strains to selected microbiota‐dependent metabolites [645]. For instance, co‐culture experiments have shown that *Streptococcus thermophilus* can degrade lactose to produce lactate, which is subsequently utilized by *Megasphaera elsdenii* to generate valerate [646]. High‐throughput metabolic profiling of large collections of cultured strains further enables the mapping of metabolic capabilities to specific microbes [645]. While simple SynComs are invaluable for mechanistic clarity, efforts are also underway to build more complex yet still defined communities, such as hCom1 and hCom2, which comprise defined collections of common human gut species [647]. Studying these communities in vitro can help characterize interaction networks and community dynamics, offering a knowledge base for future mechanistic in vivo studies [648]. However, it is important to recognize that emergent behaviors can arise in co‐culture that are not apparent from monoculture studies, underscoring the complexity of microbial ecosystems [644].

### Causal validation of host phenotypes in gnotobiotic animal models

Gnotobiotic animal models, particularly germ‐free mice raised in sterile conditions, represent a key experimental system for testing causal roles of the gut microbiota in host physiology and disease [634]. A foundational experiment in this domain is microbiota transfer, where a microbial community from a donor is transferred to a germ‐free recipient to determine whether a disease‐associated phenotype is transmissible under controlled experimental conditions [649]. This approach has provided experimental support for causal contributions of the microbiota across a range of conditions. Seminal studies showed that features of the malnutrition phenotype of kwashiorkor could be transferred from discordant human twins to recipient mice, which subsequently lost weight on a representative Malawian diet [650]. Similarly, microbiota transfer has transmitted selected phenotypes related to obesity, inflammatory conditions, and neurological or behavioral traits in experimental models, such as depressive‐like behaviors and stroke severity [634, 649, 651]. The use of these humanized mice, colonized with human‐derived microbial communities, allows for the functional interrogation of the human microbiome in a controlled in vivo context, supporting experimental causal inference about microbial communities or pathways involved in specific host phenotypes [635, 651]. Therapeutic effects can also be transmissible in experimental settings; for example, the benefits of Ganjie Decoction against RSV infection were transferable via FMT [652]. While highly informative, this methodology requires critical evaluation, as the strikingly high rate of successful phenotype transfer reported in the literature may risk overstating the microbiome's causal influence in complex human diseases, underscoring the need for rigorous experimental design and interpretation [635].

Once a causal contribution of a microbial community is supported, the subsequent challenge is to deconstruct its complexity and identify the specific strains, genes, or metabolites that contribute to the observed effects. This can be achieved by moving from whole‐community transfer to colonization of gnotobiotic animals with defined consortia and targeted strain‐ or gene‐level manipulations [647]. This reductionist in vivo approach enables prioritization of functional actors and testing of mechanistic hypotheses. Integration of microbial genetics further strengthens causal inference. For example, colonization of germ‐free mice with a human commensal engineered to lack the *cutC* gene demonstrated that this microbial pathway is required for trimethylamine production in that model, linking microbial metabolism to host TMAO levels and stroke severity [651].

Similar strategies have been applied to identify strain‐ and gene‐specific functions, although the physiological relevance of certain models, particularly those assessing gastrointestinal motility phenotypes, may not fully recapitulate human disease. More broadly, these approaches show that discrete microbial genes and pathways can be sufficient to modulate selected host phenotypes in vivo. At a high level of causal resolution, engineered microbes can be used as experimental therapeutic platforms. For instance, an engineered strain of *Escherichia coli* Nissle capable of synthesizing l‐DOPA, when co‐administered with benserazide, maintained therapeutic plasma levels and alleviated motor deficits in models of Parkinson's disease, providing experimental evidence of microbially mediated physiological modulation [632]. In parallel, reductionist approaches have identified specific effector molecules; for example, the outer membrane protein Amuc_1100 from Akkermansia muciniphila partially recapitulates selected metabolic effects attributed to the bacterium, pinpointing a defined molecular mediator [278].

### Translating preclinical causal evidence into human interventional studies

The translation of preclinical causal evidence into human therapies begins with proof‐of‐concept interventions designed to manipulate the gut microbiome. FMT stands as a primary example, demonstrating that restoring microbial community function can be an effective therapeutic strategy in selected indications [653]. Its clinical success in preventing recurrence of *Clostridioides difficile infection* provides strong human evidence that microbiota restoration can resolve disease in this specific setting [653]. However, the variable success of FMT in other conditions, such as IBD and metabolic disorders, highlights the limitations of this broad approach and underscores the need for more defined and targeted interventions [653]. This has spurred the development of next‐generation biotherapeutics, which move beyond the undefined nature of FMT. These include rationally selected LBPs comprising single strains or defined consortia of bacteria with proposed or partially validated mechanisms, such as *Akkermansia muciniphila* for metabolic health or *Faecalibacterium prausnitzii* for anti‐inflammatory functions [278, 654]. The therapeutic paradigm also extends to synbiotics, which combine probiotics with prebiotic fibers to support their engraftment and function, and postbiotics or metabolite‐based interventions. Postbiotics refer to inanimate microorganisms and/or their components, whereas purified metabolites or proteins, such as Amuc_1100, are better described as defined microbial components or metabolite‐based therapeutics [278]. An emerging component of this translational pipeline is engineered bacteria, envisioned as programmable living medicines. These microbes can be designed for controlled production and delivery of therapeutic molecules, as exemplified by engineered probiotics developed to synthesize l‐DOPA in Parkinson's disease models [632].

A major hurdle in the clinical translation of microbiome‐based therapies is inter‐individual heterogeneity, which contributes to variable treatment responses [655]. A one‐size‐fits‐all strategy is therefore unlikely to be sufficient for many complex diseases. A path forward lies in precision medicine, using biomarker‐guided approaches to stratify patient populations and personalize interventions [590]. By analyzing baseline microbiome composition or metabolite profiles, it may be possible to identify signatures that predict a patient's response to a given therapy, thereby helping distinguish likely responders from non‐responders before treatment begins [656]. For example, the baseline abundance of saccharolytic taxa such as *Bacteroides* and *Ruminococcaceae* has been associated with response to a low‐FODMAP diet in children with IBS [657]. Similarly, baseline gut microbial profiles, including the presence of specific species like *Eubacterium rectale*, may predict response to the herbal medicine JCM‐16021 for IBS‐D [658]. Microbial gene abundance, such as for oxalate‐degrading enzymes, can also predict the efficacy of a probiotic intervention for hyperoxaluria [659]. Such stratification may enhance the likelihood of detecting therapeutic effects and improve clinical trial design by reducing population heterogeneity [656]. This paradigm supports the design of more sophisticated studies in which microbial diversity or key metabolite‐related features are evaluated as predictive biomarkers and potential targets for personalized microbiome medicine [660].

### Overarching challenges in the translational pipeline

While gnotobiotic animal models are valuable for testing causality, the translation of findings from these systems to humans faces important challenges [661]. A primary concern is the risk of overstating the causal role of the microbiome in human disease based on rodent studies [635]. Human microbiota‐associated mice often exhibit a surprisingly high rate of phenotype transfer, which may not accurately reflect the more complex, multifactorial nature of human disorders [635]. Factors such as diet and host genetic background can affect model interpretation, and even subtle differences in housing conditions can substantially influence gut microbiota composition and function, sometimes leading to contradictory or non‐reproducible results [635, 662]. For example, one study demonstrated that simply housing colonized gnotobiotic mice in a specific pathogen‐free sector versus a gnotobiotic‐axenic sector of the same facility led to opposing liver phenotypes, highlighting the substantial impact of the environment [662]. These models also have inherent biological limitations. Differences in host physiology between animal models and humans make it difficult to fully recapitulate the complexity of the human gut environment and its systemic interactions [663]. This gap between animal models and human clinical reality requires a cautious approach to interpreting preclinical data and underscores the need for validation in human cohorts [635].

Beyond the scientific challenges of interspecies translation, the development of microbiome‐based therapeutics faces regulatory, ethical, and safety hurdles. Interventions involving the transfer or administration of live organisms, such as FMT and next‐generation LBPs, present unique complexities for manufacturing and clinical approval [590]. For FMT, ensuring safety and reproducibility requires rigorous donor screening, standardized material preparation, and quality control, yet protocols can vary across jurisdictions, products, and clinical settings [653]. LBPs, including engineered bacteria, must navigate complex regulatory pathways that require robust evidence of safety, purity, potency, stability, and clinical benefit, supported by well‐designed multicenter clinical trials [590]. The long‐term safety of introducing novel or engineered microbes into the human gut ecosystem remains a major concern, as the potential for unintended persistence, horizontal gene transfer, off‐target colonization, or unintended ecological effects requires careful characterization [588]. Furthermore, the intentional manipulation of an individual's microbiome raises important ethical questions regarding privacy, data governance, consent, ownership of microbiome‐derived data, and equitable access to these emerging technologies [636]. Addressing these regulatory and ethical considerations is as critical as overcoming the scientific barriers and will require close collaboration between researchers, clinicians, regulatory agencies, and other stakeholders to establish a clear and responsible path for translating microbiome‐based therapeutics into safe and effective clinical practice [656].

### Section summary

Translating microbiome discoveries into clinical practice requires a systematic and integrated framework designed to build causal evidence beyond initial associations [634]. This section has outlined such a multi‐step process, beginning with the deconstruction of clinical observations through microbiome‐wide association and multi‐omics analyses to generate mechanistic hypotheses. These hypotheses can then be tested for causal relevance in vivo using gnotobiotic animal models, progressing from whole‐community transfers to prioritize and validate functional actors. Finally, mechanistically supported findings can inform the development of microbiome‐based biomarkers, targets, and interventional strategies. This structured pipeline can help ensure that therapeutic development is grounded in mechanistic evidence, moving the field beyond correlation [664].

Importantly, this translational framework should not be viewed as a linear path but rather as an iterative, bidirectional loop. Findings from longitudinal human multi‐omics studies can provide feedback to refine hypotheses and guide further investigation in preclinical models [663]. Longitudinal human studies that integrate multiple data layers can identify novel host‐microbial pathways, such as purine metabolism in IBS, which can then be further functionally interrogated in experimental systems to prioritize new therapeutic targets [663]. This iterative cycle between the clinic and the laboratory, from bedside to bench and back again, is important for refining mechanistic understanding and optimizing therapeutic strategies.

The future of microbiome‐based medicine will likely depend on the convergence of advanced technologies and standardized methodologies. The integration of multi‐omics approaches will be important for capturing a systems‐level view of host‐microbe interactions [665]. Artificial intelligence and machine learning may play an important role in analyzing these vast and complex datasets, supporting the identification of predictive biomarkers, the modeling of microbial community dynamics, and the design of more personalized intervention strategies [636]. To realize this potential, the field should prioritize the development of harmonized standards for sample collection, data analysis, and experimental protocols to ensure reproducibility and facilitate cross‐study comparisons [636]. By adopting an integrated and iterative framework, the microbiome research community can better progress from associative findings to validated causal mechanisms and, ultimately, to more evidence‐based microbiome‐informed precision medicines.

## FUTURE DIRECTIONS AND KEY CHALLENGES

### Overview

The conceptualization of the gut microbiome as a complex, functionally integrated ecosystem has contributed to a shift in how host physiology and disease mechanisms are understood, positioning it as an important regulator of host physiology and a potential target for therapeutic intervention [666, 667]. This complex ecosystem dynamically integrates signals from host genetics, diet, and environment, and can influence host metabolism, immunity, and neurobiology [666, 668]. However, translating this extensive correlational knowledge into robust clinical applications remains challenging, hindered by fundamental unresolved challenges. To advance microbiome‐informed precision medicine, the field should address five key challenges: establishing a functional definition of health, achieving methodological standardization for cross‐study comparability, constructing a multi‐scale atlas of the microbiome organ, transitioning from correlation to mechanism‐based interventions, and bridging the bench‐to‐bedside gap through coherent clinical and regulatory pathways. Addressing these hurdles may shape the next decade of microbiome research and support its eventual integration into selected areas of clinical practice.

### Establishing a functional definition of a healthy microbiome organ

#### Beyond taxonomic lists: Toward a functional core framework

A major obstacle in microbiome science is the lack of a universally accepted definition of a healthy gut microbiome [669, 670]. Early research focused on cataloging microbial taxa, but it is now clear that simple taxonomic lists are insufficient to capture the complexity of a healthy state. Such lists can obscure the substantial functional redundancy within microbial communities, where different species can perform similar metabolic roles [670]. Consequently, there is no single microbiome configuration that defines health across all individuals [669, 670]. The field should move from compositional surveys toward functional frameworks that emphasize core metabolic and signaling pathways important for host–microbe homeostasis. Summary indices such as alpha diversity, while useful, are often poor correlates of health, as they can mask critical shifts in the abundance of disease‐associated or health‐promoting taxa [671]. A more robust definition of health will require improved understanding of the microbiome's functional output, including its capacity to produce key metabolites like SCFAs, modulate bile acid pools, and interact with the host immune system. Achieving strain‐level resolution and integrating multi‐omics data will be important to characterizing this functional core and moving beyond simplistic and often misleading definitions of dysbiosis [667, 669, 670].

#### The challenge of context: accounting for host and environmental heterogeneity

The concept of a healthy microbiome cannot be defined in isolation; it is context‐dependent, shaped by a complex interplay of host and environmental factors [670]. Substantial inter‐individual variability exists even among apparently healthy people, driven by factors such as host genetics, age, sex, diet, medications, ethnicity, and geography [669, 672]. Studies in animal models corroborate this, demonstrating that microbial composition is heavily influenced by vendor source, production facility, food, bedding, and caging, highlighting the substantial impact of environmental conditions [673, 674]. This heterogeneity complicates the search for universal biomarkers and underscores the need for population‐specific reference ranges [672]. For instance, a household‐paired study design in multiple sclerosis patients revealed that the participant's house and recruitment site were the largest sources of microbial variance, even greater than disease status itself [675]. Therefore, a functional definition of health should account for this heterogeneity, moving toward a personalized understanding where a healthy microbiome may be considered one that supports homeostasis within the specific context of its host.

#### The temporal dimension: Defining healthy trajectories versus static states

The microbiome is not a static entity but a dynamic ecosystem that evolves throughout the host's lifespan, from initial colonization in infancy to age‐related shifts in the elderly [671, 676]. Early life represents an important developmental window where the host's intestinal environment may help select for an age‐appropriate microbiome, potentially helping establish trajectories relevant to later health [676]. Studies in developing zebrafish show that bacterial communities undergo predictable assembly and turnover in tandem with host development, initially driven by deterministic processes that later become more stochastic [677]. In later life, aging is associated with transitions to different microbiome states, which may include loss of core microbes and increased abundance of disease‐associated taxa [671]. This temporal dynamism means that a single, static snapshot is insufficient to define health. Instead, a healthy microbiome may be better conceptualized as a resilient and functionally adequate system that follows a favorable developmental trajectory and can effectively resist or recover from perturbations. Future research should adopt longitudinal study designs to better map health‐related microbial trajectories, distinguish them from paths that lead toward disease, and support a more nuanced, life‐course‐aware definition of microbial well‐being [667, 671].

### Achieving cross‐study comparability through methodological standardization

#### The reproducibility challenge: Quantifying key sources of technical variation

The translational promise of microbiome research is hampered by reproducibility challenges, where findings are difficult to compare or validate across studies [678]. Methodological variations introduced at every stage of the research pipeline, from sample collection and storage to DNA extraction, sequencing, and bioinformatics, can introduce substantial biases that obscure biological signals [679, 680, 681]. Numerous studies have demonstrated that the choice of DNA extraction method alone significantly alters DNA yield, microbial diversity metrics, and the relative abundance of key taxa, particularly in the recovery of Gram‐positive bacteria [682, 683, 684, 685]. Multicenter quality control studies have quantified this problem on a larger scale, revealing high inter‐laboratory deviations in reported microbial compositions from identical samples [686, 687, 688]. The Microbiome Quality Control (MBQC) project identified biospecimen type and origin, DNA extraction, sample handling environment, and bioinformatics as major sources of technical variation [689]. This lack of standardization not only stalls clinical translation but also leads to conflicting results in the literature, making it important to systematically identify, quantify, and mitigate these sources of bias [686, 690].

#### A roadmap to harmonization: The role of reference materials and consensus protocols

Addressing reproducibility challenges requires coordinated efforts to harmonize methodologies across laboratories. A crucial first step is the development and widespread adoption of well‐characterized reference materials, including DNA and whole‐cell mock communities [691, 692]. These standards serve as important positive controls, enabling laboratories to benchmark their workflows, identify protocol‐specific biases, and ensure the accuracy of taxonomic profiling [686]. Alongside reference materials, the field needs standardized operating procedures (SOPs) for key steps of the microbiome analysis pipeline [693]. Field‐specific protocol resources have also begun to establish practical benchmarks for future investigations, including clinical human microbiome and oral microbiome studies [679, 694]. Such SOPs should cover sample collection, preservation, and DNA extraction, as these steps are major sources of variability [695]. Initiatives promoting open data and detailed protocol sharing are also vital for increasing transparency and allowing for more robust meta‐analyses [696, 697]. In this direction, the Organization for Economic Co‐operation and Development (OECD) has published the Omics Reporting Framework for regulatory toxicology studies, providing reporting elements that could inform broader omics [698]. By combining reference standards with consensus protocols, the research community may build a stronger foundation for generating reliable, comparable data, thereby supporting progress from discovery to clinical application [678, 694].

#### Beyond composition: Standardizing functional multi‐omics readouts

As microbiome research moves beyond 16S rRNA gene profiling to investigate the functional activities of the microbial community, the need for standardization extends to multi‐omics technologies such as shotgun metagenomics, metatranscriptomics, metaproteomics, and metabolomics. Each of these layers presents unique methodological challenges that can impact data quality and comparability. For instance, fecal metaproteomics requires effective sample stabilization methods to minimize metaproteome alterations during storage and shipment, as well as optimized protein cleanup and digestion protocols to ensure comprehensive and reproducible analysis [699, 700]. Similarly, metabolomics studies require validated, high‐throughput extraction and analytical workflows to measure the broad array of microbial, host‐derived, dietary, and xenobiotic small molecules in biological samples [701, 702]. Without standardized approaches for these functional readouts, the field risks repeating the reproducibility issues that have plagued compositional studies. Therefore, benchmarking, developing, and validating standardized protocols for these advanced omics platforms is an important next step to ensure that functional data are robust, reliable, and comparable across large‐scale cohort studies.

#### Data integration and standardization for multi‐center multi‐omics studies

Beyond experimental standardization, a major bottleneck in microbiome translation lies in the integration of heterogeneous multi‐omics datasets generated across centers, platforms, and populations. Addressing this challenge requires harmonization at the levels of metadata, data structure, and computational analysis [636, 694]. Standardized metadata frameworks should systematically capture key covariates, including diet, medication exposure, clinical phenotypes, and environmental factors, using controlled vocabularies and unified ontologies [415, 636, 694]. Such harmonization is essential for enabling cross‐cohort comparability and large‐scale meta‐analysis [636, 694].

At the computational level, robust strategies are required to mitigate batch effects and platform‐specific biases [521, 663]. Methods such as compositional data transformation, cross‐study normalization, and empirical Bayes‐based correction approaches can reduce technical variability, while dimension‐reduction, latent‐variable, alignment approaches, and latent‐space embedding can enable integration across datasets with partially overlapping features. Importantly, integration should move beyond simple concatenation toward model‐based frameworks that preserve modality‐specific information while enabling joint inference [521, 525].

Integrative analysis of multi‐omics data further requires computational frameworks capable of capturing the multi‐layered architecture of host–microbiome interactions [471, 525]. Multi‐view learning approaches, network‐based models, and latent variable frameworks provide scalable solutions for linking microbial taxa, metabolites, gene expression, and clinical phenotypes into unified analytical structures [471, 525, 636]. These approaches enable identification of reproducible cross‐omic signatures and may improve the robustness of predictive biomarkers across populations [535, 636].

In parallel, longitudinal cohort design is important for strengthening causal inference and enhancing translational relevance [636, 663]. Repeated sampling across defined temporal windows, combined with controlled perturbations such as dietary interventions or therapeutic exposures, allows characterization of dynamic microbiome states and host responses [636, 663]. Standardization of sampling protocols, storage conditions, and sequencing workflows is essential to minimize technical variability across time points and study sites [694, 695]. Integration of longitudinal multi‐omics data with clinical endpoints facilitates identification of temporal trajectories associated with disease onset, progression, and treatment response [636, 663].

Finally, ensuring generalizability requires systematic validation across diverse populations [414, 636]. Given substantial geographic and inter‐individual variability, microbiome‐based models should undergo external validation and, where necessary, population‐specific recalibration to enable cross‐population prediction [414, 636]. Together, these experimental and computational strategies can establish a practical framework for translating microbiome research into reproducible and clinically actionable insights.

### Constructing a multi‐scale microbiome organ atlas

#### Spatiotemporal mapping: From gut biogeography to developmental dynamics

The gut is not a homogeneous environment; it is a complex landscape with distinct anatomical niches, each harboring specialized microbial communities. A comprehensive atlas of the microbiome organ‐like system should therefore capture its spatiotemporal organization. Studies sampling multiple gastrointestinal sites in humans and other mammals have revealed substantial biogeographical structuring, with microbial composition, diversity, and function varying markedly among anatomical regions [703]. High‐resolution analyses using colonoscopy aspirates have shown that genetically distinct yet related bacterial strains can colonize different sites within the same individual, indicating site‐associated strain‐level differentiation [704]. This spatial map must also be integrated with a temporal dimension that charts the microbiome's development across the host's lifespan. The microbiome undergoes developmentally patterned changes from early life toward adulthood, a process closely linked to healthy host development [676, 677]. Mapping these dynamic developmental trajectories alongside the spatial organization of the gut will provide a four‐dimensional atlas that will support understanding how perturbations in specific locations or at critical life stages contribute to disease.

#### Systems‐level integration: A multi‐omics framework for decoding the gut–organ axis

The influence of the gut microbiome can extend beyond the intestine, communicating with distant organs through complex signaling networks known as gut–organ axes [705]. Decoding these systemic interactions requires a systems‐level approach that integrates multi‐omics data from the microbiome with corresponding data from host tissues. For example, integrating gut metagenomic data with host transcriptomic, metabolomic, and epigenomic data has begun to reveal associations among microbiome‐related alterations, systemic inflammation, and cognitive decline in aging [668], while integrating gut microbiome and host transcriptomic/epigenomic data can help characterize host–microbiome alterations in IBD [706]. Such integrative frameworks can identify molecular signatures that connect microbial functions to host pathways, providing a more integrated understanding of disease pathogenesis [707]. This systems biology approach is essential for mapping the intricate crosstalk along axes like the gut–brain, gut–liver, and gut–skin, ultimately revealing how the gut microbiome organ‐like system may modulate host health and disease [707].

#### The role of mechanistic models in deconstructing complexity

Constructing a multi‐scale atlas generates vast and complex datasets that require advanced models for interpretation. Computational and in vitro models are valuable tools for deconstructing this complexity and moving from correlational observations to mechanistic understanding [707]. Mathematical and ecological network models can be used to simulate system behavior, analyze large multi‐omics datasets, and identify candidate microbial interactions that shape community dynamics [707, 708]. In parallel, advanced *in vitro* systems like gut‐on‐a‐chip platforms provide a means to experimentally validate these interactions in a physiologically relevant microenvironment. These microfluidic devices can replicate key aspects of the intestinal niche, including 3D epithelial structure and mechanical forces like gut motility, allowing researchers to study host‐microbe and inter‐organ signaling under controlled conditions [709, 710]. By integrating computational modeling with these sophisticated experimental platforms, researchers can test molecular mechanisms underlying gut–organ axes and build more predictive, mechanistic models of the microbiome organ‐like system.

### Transitioning to mechanism‐based precision microbiome medicine

#### From correlation to causality: A framework for stratifying heterogeneous responses

A significant barrier to the clinical application of microbiome‐based therapies is the substantial heterogeneity observed in patient responses [711]. Interventions such as prebiotics or dietary changes often yield beneficial effects in only a subset of individuals, highlighting the need to move beyond a one‐size‐fits‐all approach [712]. Recent studies suggest that this variability is not entirely random and is influenced by the recipient's baseline gut microbiome composition and function [713]. The efficacy of resistant starch in improving MASLD‐related phenotypes may be influenced by specific microbial taxa involved in its utilization and degradation [713]. Microbiome‐related changes have also been associated with clinical improvement in metabolic liver disease [714]. These findings provide a framework for stratifying patients into responder and non‐responder groups based on their microbial profiles before treatment initiation, an approach conceptually aligned with pharmaco‐metabonomic phenotyping [715]. Such microbiome‐based stratification may complement established clinical risk‐prediction approaches and support the development of mechanism‐informed precision nutrition and medicine [716].

Beyond stratification, a key unresolved issue is the biological basis of this variability across individuals [471]. Differences in microbial functional capacity, ecological stability, and host physiological context create distinct response landscapes in which identical interventions can produce divergent outcomes [471, 665]. For example, the metabolic response to dietary substrates depends not only on the presence of specific taxa but also on the activity of relevant enzymatic pathways and the surrounding microbial network [611, 665]. Similarly, probiotic engraftment is constrained by ecological resistance within the resident microbiome and by host immune regulation [616, 617]. These factors indicate that variability arises from systems‐level properties rather than single microbial features [471, 665]. Understanding these determinants will be important for designing interventions that are not only stratified but also mechanistically informed, thereby advancing the development of precision microbiome medicine [471].

#### The synergy of multi‐omics and machine learning for developing predictive biomarkers

Identifying the microbial features that predict therapeutic response requires the analysis of high‐dimensional data, a task for which machine learning (ML) can be useful [717]. The integration of multi‐omics datasets, including metagenomics, metatranscriptomics, and metabolomics, with advanced ML algorithms provides a useful framework for biomarker discovery [718]. These approaches can identify candidate microbial signatures, metabolic pathways, and host–microbe interaction networks that are associated with clinical outcomes, such as disease subtypes in IBD or response to ICIs in cancer [717, 718]. For example, ML‐based analyses of microbial and clinical data have been used to stratify patients across the Alzheimer's disease continuum and to predict clinical outcomes in Helicobacter pylori infections [719, 720]. By leveraging the synergy between deep molecular profiling and sophisticated computational analysis, researchers may develop more robust and externally validated biomarkers that support patient stratification and inform intervention selection. These models should be trained and validated across harmonized multi‐center datasets to ensure robustness and clinical generalizability.

#### Designing next‐generation, mechanism‐informed interventions

A mechanistic understanding of host–microbe interactions should inform the design of more targeted microbiome therapies. Many probiotic and psychobiotic interventions lack a clear mechanistic rationale, which may contribute to their variable efficacy [666, 721]. A precision medicine approach requires moving toward mechanism‐informed interventions, where specific microbes or microbial consortia are selected based on their known functional capabilities and their ability to modulate specific host pathways [666]. For example, decoding the cellular and genetic pathways regulated by the microbiome during acute liver failure revealed a MYC‐dependent program that could be pharmacologically targeted in experimental models to attenuate acute liver failure [722]. Similarly, identifying specific microbial strains that produce or modulate gut–brain‐relevant metabolites is important for the rational selection of psychobiotics to target the gut–brain axis [721]. This targeted, mechanism‐informed approach may support the development of more reliable interventions, from engineered microbes to personalized nutritional plans, designed to modulate microbiome functions for therapeutic benefit.

### Bridging the bench‐to‐bedside gap through clinical and regulatory pathways

#### Establishing clinical utility: The need for rigorous trial designs and endpoints

The translation of promising preclinical findings into clinically validated therapies requires rigorously designed and adequately powered clinical trials. Many current microbiome studies are limited by small sample sizes, methodological heterogeneity, and a lack of independent replication or longitudinal validation, which limits the ability to establish robust clinical utility [723]. Future trials should increasingly use precision‐medicine frameworks that incorporate biomarker‐guided patient stratification to account for the heterogeneous responses to interventions such as FMT [711]. Large, prospective, multicenter studies will be important to develop and validate predictive models of treatment response across diverse populations [723]. In addition, standardized protocols for intervention design, including donor selection, preparation methods, and delivery routes, are required to minimize confounding and enable cross‐study comparability [711]. Integration of multi‐omics data into trial design will further support the linkage of microbial function to clinical endpoints and facilitate biomarker‐driven stratification.

Despite these efforts, the clinical efficacy of microbiome‐based interventions remains highly variable outside of recurrent *Clostridioides difficile* infection, where FMT and approved microbiota‐based products have demonstrated clear benefit for preventing recurrence after standard antibacterial therapy [593]. In other conditions, including inflammatory, metabolic, and selected neuropsychiatric or gut–brain axis‐related disorders, outcomes have been less consistent, with many studies reporting lower or less durable effects [585, 593, 654, 724]. These challenges reflect multiple factors, including donor variability, inconsistent engraftment, recipient‐specific microbiome and immune contexts, and disease heterogeneity. The limited availability of approved microbiome‐based or LBPs beyond recurrent *C. difficile* infection, despite extensive preclinical and clinical investigation, further highlights the difficulty of translating microbiome science into reproducible therapies. Collectively, these observations suggest that current limitations may not only be technical but may also reflect fundamental challenges inherent to targeting a complex and context‐dependent microbial ecosystem [585, 593, 654].

#### Navigating the regulatory landscape: Frameworks for microbiome‐based therapeutics

As microbiome‐based interventions such as fecal microbiota transplantation advance, navigating the regulatory landscape has become a major challenge [725]. Regulatory frameworks are evolving in response to increasing recognition of microbiome‐based products as therapeutic candidates and the microbiome as a consideration in risk assessment [165, 725]. For example, regulatory discussions around fecal microbiota transplantation highlight unresolved issues in classification, safety, quality control, and standardization [725]. Similarly, the European Food Safety Authority has proposed incorporating microbiome considerations into the risk assessment of food‐related compounds [726].

Despite these developments, conventional FMT and emerging microbiome‐based therapies still face complex and jurisdiction‐specific regulatory pathways, with standards varying substantially across jurisdictions [727]. In the United States, FDA enforcement discretion applies under limited circumstances to conventional FMT for Clostridioides difficile infection not responding to standard therapies and does not apply to stool bank‐derived FMT; use for other indications generally requires an investigational new drug application, creating uncertainty for broader clinical translation [727, 728]. The absence of standardized protocols for donor screening, material processing, and long‐term safety monitoring presents significant barriers to both clinical implementation and regulatory approval [727, 728]. Additional challenges include a lack of harmonized analytical methods, insufficiently validated microbiome‐related biomarkers, and limited understanding of causal host–microbiome interactions [165, 729]. Developing more coordinated regulatory frameworks that address safety, reproducibility, efficacy, and innovation will be important for advancing microbiome‐based therapeutics.

#### A call for prudent clinical integration and evidence‐based guidelines

The integration of microbiome science into clinical practice requires a cautious and evidence‐based approach. Clinicians will need clear, validated guidelines to inform decisions related to antibiotic stewardship, dietary interventions, and the use of emerging microbiome‐targeted therapies. Premature or unregulated application of microbiome‐targeted therapies risks outpacing scientific validation and may expose patients to unintended harm [728]. Coordinated efforts among researchers, clinicians, and policymakers will therefore be necessary to translate robust scientific evidence into actionable clinical strategies that ensure safe, effective, and equitable implementation of microbiome‐based medicine.

### Section summary

Advancing microbiome‐informed systems medicine will require a coordinated research agenda [636, 718]. The next decade should prioritize collective efforts to address the grand challenges outlined herein, forming a consensus roadmap for the field [636]. This roadmap begins with establishing a functional, context‐aware definition of a healthy microbiome that embraces its dynamic nature across the lifespan [669, 670]. It requires sustained commitment to methodological standardization, through reference materials and consensus protocols, to build a foundation of reproducible and comparable data across all omics layers [636, 687, 694]. Building on this foundation, the field should construct a multi‐scale, spatiotemporal atlas of the microbiome organ, integrating multi‐omics data with mechanistic models to decode its systemic influence [636, 718]. This may support the transition toward mechanism‐based precision medicine, where interventions are rationally designed and targeted to individuals based on predictive biomarkers [636, 708, 718]. Finally, clearer clinical and regulatory pathways will help translate scientific advances into safe, effective, and accessible therapies, supporting the responsible development of microbiome‐informed approaches to improve human health.

## CONCLUSION

Conceptualizing the gut microbiome as an organ‐like functional system provides a unifying framework for integrating its structural organization, metabolic capacity, and systemic signaling roles in human physiology and disease. The gut microbiome organ operates through interconnected metabolic, immune, and neuroendocrine circuits, translating environmental inputs into local and systemic host effects via molecular mediators and feedback mechanisms. Advances in multi‐omics profiling, spatial and single‐cell approaches, and causal modeling are beginning to resolve the functional modules and regulatory logic of this organ, helping move the field beyond associative descriptions toward mechanistic understanding. Importantly, this framework also clarifies the translational path forward, emphasizing the need to define healthy microbiome functional states, standardize functional readouts, and align experimental models with clinically relevant endpoints. As these challenges are progressively addressed, targeting microbiome‐derived functional circuits, rather than individual taxa alone, has the potential to advance precision medicine by supporting rational, durable, and systems‐level interventions for selected metabolic, inflammatory, and gut–brain axis‐related disorders.

## AUTHOR CONTRIBUTIONS

**Yang Bi:** Conceptualization; investigation; data curation; writing—original draft; visualization. **Weibin Song:** Investigation; data curation; writing—review and editing. **Maria Glymenaki:** Investigation; writing—review and editing. **Jie Hu:** Investigation; writing—review and editing. **Xiru Li:** Data curation; visualization. **Heng Huang:** Data curation; visualization. **Yousong Peng:** Investigation; writing—review and editing. **Shuaishuai Ni:** Investigation; writing—review and editing. **Ornuma Haonon:** Writing—review and editing. **Zhu Liu:** Visualization. **Krai Daowtak:** Investigation; writing—review and editing. **Xiaotao Shen:** Investigation; writing—review and editing. **Huadong Peng:** Investigation; writing—review and editing. **Jutarop Phetcharaburanin:** Investigation; writing—review and editing. **Huiru Tang:** Methodology; formal analysis; writing—review and editing. **Yulan Wang:** Methodology; formal analysis; writing—review and editing. **Jia V. Li:** Methodology; formal analysis; writing—review and editing. **Mizu Jiang:** Supervision; writing—review and editing. **Nick Powell:** Supervision; writing—review and editing. **Victor W. Zhong:** Supervision; writing—review and editing. **Elaine Holmes:** Conceptualization; supervision; writing—review and editing. **Yunlei Xianyu:** Investigation; writing—review and editing. **Zhigang Liu:** Conceptualization; supervision; project administration; funding acquisition; writing—original draft; writing—review and editing.

## CONFLICT OF INTEREST STATEMENT

Maria Glymenaki is employed with the European Food Safety Authority (EFSA) in the Nutrition and Food Innovation Unit that provides scientific and administrative support to the Panel on Nutrition, Novel Foods, and Food Allergens, in the area of Novel Foods. However, the present work is published under the sole responsibility of the authors and may not be considered as an EFSA scientific output. The positions and opinions presented in this article are those of the authors alone and do not necessarily represent the views or scientific work of EFSA. Elaine Holmes is a director of Melico Sciences. The remaining authors declare no conflicts of interest.

## ETHICS STATEMENT

No animals or humans were involved in this study.

## Data Availability

Data sharing is not applicable to this article as no new data sets were created or analyzed in this study. Supplementary materials (graphical abstract, slides, videos, Chinese translated version, and updated materials) may be found in the online DOI or *iMeta* Science http://www.imeta.science/.
